# The Genome of the Myxosporean *Thelohanellus kitauei* Shows Adaptations to Nutrient Acquisition within Its Fish Host

**DOI:** 10.1093/gbe/evu247

**Published:** 2014-11-08

**Authors:** Yalin Yang, Jie Xiong, Zhigang Zhou, Fengmin Huo, Wei Miao, Chao Ran, Yuchun Liu, Jinyong Zhang, Jinmei Feng, Meng Wang, Min Wang, Lei Wang, Bin Yao

**Affiliations:** ^1^Key Laboratory for Feed Biotechnology of the Ministry of Agriculture, Feed Research Institute, Chinese Academy of Agricultural Sciences, Beijing, People’s Republic of China; ^2^Key Laboratory of Aquatic Biodiversity and Conservation, Institute of Hydrobiology, Chinese Academy of Sciences, Wuhan, People’s Republic of China; ^3^Tianjin Biochip Corporation, Tianjin, People’s Republic of China; ^4^TEDA School of Biological Sciences and Biotechnology, Nankai University, Tianjin, People’s Republic of China

**Keywords:** Myxozoa, genome and transcriptome, nutrient deprivation, food-borne illness

## Abstract

Members of Myxozoa, a parasitic metazoan taxon, have considerable detrimental effects on fish hosts and also have been associated with human food-borne illness. Little is known about their biology and metabolism. Analysis of the genome of *Thelohanellus kitauei* and comparative analysis with genomes of its two free-living cnidarian relatives revealed that *T. kitauei* has adapted to parasitism, as indicated by the streamlined metabolic repertoire and the tendency toward anabolism rather than catabolism. *Thelohanellus kitauei* mainly secretes proteases and protease inhibitors for nutrient digestion (parasite invasion), and depends on endocytosis (mainly low-density lipoprotein receptors-mediated type) and secondary carriers for nutrient absorption. Absence of both classic and complementary anaerobic pathways and gluconeogenesis, the lack of de novo synthesis and reduced activity in hydrolysis of fatty acids, amino acids, and nucleotides indicated that *T. kitauei* in this vertebrate host–parasite system has adapted to inhabit a physiological environment extremely rich in both oxygen and nutrients (especially glucose), which is consistent with its preferred parasitic site, that is, the host gut submucosa. Taking advantage of the genomic and transcriptomic information, 23 potential nutrition-related *T. kitauei*-specific chemotherapeutic targets were identified. This first genome sequence of a myxozoan will facilitate development of potential therapeutics for efficient control of myxozoan parasites and ultimately prevent myxozoan-induced fish-borne illnesses in humans.

## Introduction

The international market for fish and fish products has grown remarkably, and global fish consumption was estimated to be 140.5 million tons in 2013 (http://www.thefishsite.com/reports/?id=2253&country=WW). Along with the rapid growth in fish consumption, incidence of fish-borne parasitic zoonoses also increased (Dos Santos and Howgate 2011). Of the big family of fish parasites, myxozoans constitute an important group including more than 2,100 species in 58 genera, most of which are coelozoic or histozoic parasites in fish. The life cycles of myxosporeans involve two distinct spore stages, the actinospore in an invertebrate host (usually an annelid), and the myxospore in the vertebrate host (Lom and Dykova 2006). Although most myxozoans are not highly pathogenic for fish hosts, some cause disease and impact upon wild and farmed fish populations (Gomez et al. 2014). The impact of myxozoans has further exacerbated, as indicated by the observed increase in fish-to-fish transmission rate as well as the emergence of fish-to-human transmission (i.e., the recent outbreaks of food poisoning in Japan associated with *Kudoa Septempunctata*; Kent et al. 2001; Lom and Dykova 2006; Sitja-Bobadilla 2008; Kawai et al. 2012).

Myxozoa has been confirmed as Metazoa rather than Protozoa by the detected metazoan characteristics, multicellularity of some life stages, tight junctions, collagen production (Siddall et al. 1995; Kent et al. 2001; Canning and Okamura 2004), and phylogenetic analyses (Smothers et al. 1994). However, the placement of Myxozoa within Metazoa, either as a sister taxon to Bilateria or within Cnidaria, was still controversial (Jimenez-Guri et al. 2007; Evans et al. 2010; Nesnidal et al. 2013). Recent phylogenomic data supported to place Myxozoa within Cnidaria. However, these phylogenomic analyses either only used expressed sequence tag data which could not reject a bilaterian origin of Myxozoa with the topology test (Jimenez-Guri et al. 2007) or was very weakly supported by a Bayesian inference analysis with the CAT model (Nesnidal et al. 2013). Therefore, the relative phylogenetic position of Myxozoa within Metazoa remains unresolved and is compelling for further study.

In China, more than 600 species of myxozoan parasites have been reported to infest freshwater and marine fish, and some of them cause severe economic losses in the aquaculture industry (Chen and Ma 1998). Particularly, intestinal giant-cystic disease, caused by the myxozoan species *Thelohanellus kitauei*, has been recognized as the most detrimental disease of farmed carp (Egusa and Nakajima 1981; Chun et al. 1988; Shin et al. 2011), the most commonly farmed fish species with the highest production in China. Around 20% of farmed carp were killed annually due to this disease, leading to an economic loss of approximately 50 million dollars in 2010. Infected carps suffer from profound intestinal swelling and malabsorption (Egusa and Nakajima 1981; Chun et al. 1988; Shin et al. 2011). In addition to an increased erythrocyte sedimentation rate, other hematological parameters such as red blood cells, hemoglobin, hematocrit, albumin, total protein, triglycerides, phosphorus, calcium, and biological mass all decreased significantly (*P* < 0.05) in *T. kitauei*-infected carp (Wang et al. 2005). These changes, associated with severe anemia and cacotrophia, lead to secondary enteritis and ultimately death of infected fish (Egusa and Nakajima 1981; Chun et al. 1988; Wang et al. 2005).

In contrast to their evolutionary and economic significance, knowledge of the general biology of myxozoans is very limited, largely due to the inability to culture these parasites in vitro. Genomic studies represent a promising strategy to investigate the biology and metabolism of myxozoans. However, no genome sequence of any myxozoan has been available. Here, we present the genome sequence of *T. kitauei*. Genomic analysis was conducted to provide insights into the biology of this myxozoan parasite, focusing on the nutrition and metabolism aspect. Phylogenomic analyses using new genomic data of *T. kitauei* and comparative analyses of minicollagen and mesodermal genes robustly confirmed Myxozoa to be a highly derived Cnidarian taxon, a sister group of Medusozoa (Feng et al. 2014).Taking advantage of this information, we conducted comparative genomic analysis between the parasitic *T. kitauei* and its two free-living cnidarian relatives, that is, *Hydra magnipapillata* (Chapman et al. 2010) and *Nematostella vectensis* (Putnam et al. 2007) to investigate the genomic evolution associated with parasitic lifestyle. Also, aided by transcriptome sequencing of the myxospore stage, we identified a range of potential *T. kitauei* chemotherapeutic targets, which may facilitate the development of novel therapeutic agents against myxozoan parasites and relieve the potential threat of myxozoan zoonoses to human consumers.

## Materials and Methods

### Parasite Collection

Infected common carp were collected from a approximately 100-acre aquaculture pond (∼1.5 m deep, 30 °C, pH 7.5–8.0) in Wuqing, Tianjin, China in July 2007. After euthanasia, the intestines of infected fish were obtained by dissection. Cysts found on the surface of the intestines were carefully collected from the intestinal tissue and rinsed with sterile water. The cysts were cut open and spores were washed out with sterile phosphate-buffered saline (pH 7.0). The myxospore suspensions were further filtered with a 300 -µm mesh sieve to remove residual host fish tissue. The filtrates were centrifuged at 6,000 × g for 10 min, and the myxospore pellets were resuspended in 9.5 ml of sterile phosphate-buffered saline (pH 7.0). Host cells were lysed by addition of 0.5 ml of 20% sodium dodecyl sulfate and incubated at 25 °C for 10 min. After centrifugation at 8,000 × g for 10 min, the supernatants were discarded and spores were resuspended in 10 ml of sterile phosphate-buffered saline. The centrifugation and resuspension were repeated once. Finally, the myxospores were microscopically with care examined to ensure that host fish tissue and cells had been removed.

### DNA and RNA Extraction

Spore samples (2 g wet weight) were frozen in liquid nitrogen and then ground with a mortar and pestle. DNA was extracted as described previously (Yap and Thompson 1987). The purity and yield of the DNA was assessed spectrophotometrically. The samples were checked by polymerase chain reaction (PCR) with host-specific primers to further ensure that the samples did not contain host fish DNA (Rag1-R: 5′-GACACTATGGAGAAAGGGAGGTGGAGTT-3′; Rag1-F: 5′-GGGAAGCAGAGGTCGCAGTTGGAGG-3′) (Fan et al. 2009).

For RNA extraction, purified spores (1 g wet weight) were frozen in liquid nitrogen and ground with a mortar and pestle. The lysate was transferred to a 1.5-ml RNase-free microcentrifuge tube and homogenized in 350 μl Buffer RLT Plus (Qiagen) with a vortex mixer (MVS-1, Beijing Beide Science Co.). RNA was purified using the RNeasy Plus Mini kit (Qiagen). RNA concentration and purity were assessed with a Nanodrop spectrophotometer (Thermo Scientific).

### Species Identification

The species of the spores was determined by morphological examination via light and scanning electron microscopy (supplementary material and fig. S1, Supplementary Material online) and by phylogenetic analysis of 18S rDNA sequences focusing only on *Thelohanellus* spp. by RAxML version 7.2.6 (Stamatakis 2006) with the best-fitted model GTRGAMMAI.

### Genome Sequencing

For Roche 454 library construction and sequencing, two libraries (8- and 20-kb fragments) were prepared using the GS FLX Titanium Library Preparation kit according to the manufacturer’s protocols (Roche Applied Science, Mannheim, Germany). The purified genomic DNA is fragmented by hydrodynamic shearing to generate 8 kb and 20 kb span paired-end libraries. Quantitated DNA fragments from the paired-end library, flanked with proper amplification and sequencing adaptors, were immobilized onto microspheres (beads) and the entire bead-bound library was then emulsified with the amplification reagents (GS FLX Titanium LV emPCR kit). After the emPCR step, the DNA-carrying beads were cleaned and enriched before loaded into the wells of a PicoTiterPlate device (GS FLX Titanium PicoTiterPlate Kit [70 × 75]) at the density of no more than a single DNA bead per well. The loaded PTP device was then inserted into the Genome Sequencer FLX Instrument, and sequencing reagents were sequentially flowed over the plate. The sequencing was conducted using the GS FLX Titanium Sequencing Kit and the Genome Sequencer FLX Instrument strictly following the recommendations of the manufacturer (Roche Applied Science, Mannheim, Germany). The raw output comprising a set of digital images (PIF files) was processed to obtain the sequence of the DNA library fragments/reads and then subjected to downstream analysis. The quality of the data obtained from the sequencing was verified using the Roche 454 Sequencing System Software version 2.6 (Roche 454 Life Sciences, Branford, CT, USA), including normalization, correction, and quality filtering steps, to generate Standard Flowgram Format files containing the base called read sequences and per base quality scores as described in the Software manual.

For short-insert (∼500 bp) DNA libraries, that is, standard DNA libraries, we followed the manufacture’s protocol (Illumina). Briefly, 5 μg of genomic DNA was fragmented by nebulization with compressed nitrogen gas. The DNA ends were polished and an “A” base was added to each end of the fragments. Next, the DNA adaptors (Illumina) with a single “T” overhang at the 3′-end were ligated to the products above. The ligation products were separated on a 2% agarose gel and an approximately 2-mm wide gel slice containing DNA of the desired size was excised (Qiagen Gel Extraction Kit). In this step, DNA fragments in a narrow size range were selected to facilitate the downstream assembly process. For long (∼3 Kb) mate-paired libraries, the manufacture’s mate pair library kit (Illumina) was used. Around 10–30 μg genomic DNA was fragmented by HydroShear and polished with biotin labeled dNTPs. The fragments in the size range of 2–5 Kb were selected by gel excision. The size-selected DNA fragments were circularized by intramolecular ligation of the blunt ends, and the remaining linear DNA fragments from the circularization reaction were digested by DNA exonuclease. Then, the circularized DNA was randomly fragmented to lengths of approximately 400 bp, and the fragments that contain the biotinylated ends of the original size-selected fragment were purified using streptavidin-coated magnetic beads. The fragments bound to the streptavidin beads were end-repaired and A-tailed, followed by attachment of the illumine paired-end oligo adapters by ligation. After the adapter ligation, PCR was carried out to simultaneously enrich and amplify those DNA fragments with adapter molecules on both ends. The product of the PCR amplication is a DNA smear of different fragment sizes. Fragments in the range of 350-650 bp were excised from the gel for cluster formation and sequencing. For quality control of Illumina sequence data, the adaptors were stripped with SeqPrep (http://omictools.com/seqprep-s1982.html), and ConDe Tri_v2.1.pl (Smeds and Kunstner 2011) was used to trim low-quality end sequences of the reads with a Phred quality score lower than 20, as well as non-A/T/G/C bases. Reads with a length lower than 25 bp after trimming were discarded. Paired reads with overlap were merged into single reads by SeqPrep.

### Transcriptome Sequencing

mRNA was isolated from total RNA using Oligo-dT beads (Invitrogen) and then fragmented by heating at 94 °C. First-strand complementary DNA (cDNA) was synthesized with random hexamer primers and second-strand cDNA synthesized with DNA polymerase. Double-stranded cDNA was end-repaired/blunt-ended with Klenow polymerase, T4 DNA polymerase, and T4 polynucleotide kinase. A single adenosine moiety was added to the cDNA using Klenow exo^-^ and dATP. All the enzymes used above were included in the kit (mRNA-Seq Sample Pre Kit) from Illumina. Illumina adapters (containing primer sites for sequencing and flowcell surface annealing) were ligated onto the blunt-ended cDNA, and the library of DNA fragments (200–250 bp) was separated from unligated adapters by gel excision. Libraries were amplified by PCR with Phusion polymerase, denatured with 100 mM sodium hydroxide, and diluted in hybridization buffer before they were loaded onto a single lane of an Illumina GA flowcell. Cluster formation, primer hybridization, and paired-end sequencing (101 × 2 cycles) were performed using Illumina reagents. Expression level was determined using the mapped reads per kilobase of exon per million mapped reads (RPKM) in the spore cells.

### Genome Assembly

The paired-end reads and mate-paired reads from Solexa sequencing were assembled using SOAPdenovo software, which uses the de Bruijn graph data structure to construct contigs (Li et al. 2010). SOAPdenovo was used to assemble short reads generated by the Illumina Genome Analyzer. Contigs produced by SOAPdenovo were split into 2-kb windows with 1-kb overlap with the EMBOSS splitter program (Rice et al. 2000). The Roche 454 reads and the split Illumina sequences were then processed with the Newbler sequence assembler (version 2.3) to produce super-reads in a hybrid assembly. Finally, gaps between contigs were filled by mapping and local assembly of the Solexa sequencing reads using GapCloser (http://soap.genomics.org.cn/soapdenovo.html) in iterative mode. The GC content of each assembled scaffold was calculated. The mean GC content was 28.8% with a standard deviation of 5.9%. Only sequences with GC content within three standard deviations of the mean were included in the genome assembly, and 70 scaffolds with GC content higher than 47% were discarded (0.11 Mb total).

Repetitive sequences were identified by searching Repbase using RepeatMasker (http://www.repeatmasker.org/) with default parameter settings and by de novo repetitive sequence search using Repeat Modeler (http://www.repeatmasker.org/RepeatModeler.html).

### Gene Prediction

A strategy combining RNA-Seq and de novo approaches was used to predict protein-coding genes. First, the RNA-Seq reads were assembled to generate the training set for the de novo algorithms. Generally, a combination of mapping-based assembly (RNA-Seq reads were mapped to reference genome and assembled) and de novo methods were used. For mapping-based assembly, RNA-Seq reads were mapped to the assembled genome using SOAPsplice (Huang et al. 2011), allowing up to two mismatches, and assembled using the Cufflinks software (http://cufflinks.cbcb.umd.edu/). For de novo assembly, transcripts were constructed using Trinity software (Grabherr et al. 2011). The assembled transcripts obtained by the two methods were pooled and validated by aligning them to the assembled genome with PASA (http://pasa.sourceforge.net/) and GMAP (Wu and Watanabe 2005). The PASA-recommended best transcript candidates were used to train the gene prediction programs. Four programs were utilized for de novo gene prediction, that is, GeneMark (Lukashin and Borodovsky 1998), Augustus (Stanke et al. 2008), GlimmerHMM (Majoros et al. 2004), and SNAP (Korf 2004). The final gene set was created by merging all of the gene sets using Evidence Modeler (http://evidencemodeler.sourceforge.net/).

### Functional Annotation

Functions were assigned and classified by searching for sequence similarity with BLAST (http://blast.ncbi.nlm.nih.gov/Blast.cgi) against the eggNOG database (http://eggnog.embl.de), Kyoto Encyclopedia of Genes and Genomes (KEGG) reference database (http://www.genome.jp/kegg/), and the National Center for Biotechnology Information (NCBI) nonredundant protein sequence database (ftp://ftp.ncbi.nlm.nih.gov/blast/db/). Gene Ontology (http://www.geneontology.org/) was used to classify and functionally annotate genes. Protein domains were predicted using the Pfam database (http://pfam.sanger.ac.uk/) for gene function assignment. The tRNA and rRNA genes were predicted with tRNASCAN-SE (http://lowelab.ucsc.edu/tRNAscan-SE/). Potential secreted proteins were predicted by SignalP v4.1 (http://www.cbs.dtu.dk/services/SignalP/) (Petersen et al. 2011) and PrediSi (http://www.predisi.de/) (Hiller et al. 2004). Predicted proteins were classified as proteases by querying the MEROPS database (http://merops.sanger.ac.uk/) (Rawlings et al. 2014) with BLASTp with cut-off *E* value as 1 e ^−^^5^. If a predicted protein was found to have more than one functional domain, only the domain with the lowest *E* value was retained. Putative α/β-hydrolases were classified according to BLASTp results (*E* value cut-off = 1 e ^−^^3^) obtained against the Lipase Engineering database (http://www.led.uni-stuttgart.de/). Carbohydrate-active enzymes (carbohydrate esterases, glycoside hydrolases, glycosyltransferases, polysaccharide lyases, and carbohydrate-binding modules) were annotated using the dbCAN Web server (http://csbl.bmb.uga.edu/dbCAN/) (Yin et al. 2012) at an *E* value cut-off of 1 e ^−^^5^. Transporters were predicted from the genome sequence by the TransportTP tool (http://bioinfo3.noble.org/transporter/) (Li et al. 2009). Reference organisms for our TransportTP analysis were: *Escherichia coli*, *Saccharomyces cerevisiae*, *Arabidopsis thaliana*, *Oryza sativa*, *Caenorhabditis elegans*, *Drosophila melanogaster*, and Homo sapiens (*E* value threshold = 0.1).

## Results and Discussion

### Species Identification

Cysts of an unknown origin were collected from the intestines of common carp and examined for parasitic infection. Isolated myxosporean-like spores from the cysts showed morphological and morphometric features nearly indistinguishable from those of the previously described *T. kitauei* (supplementary fig. S1*A* and *B* and table S1, Supplementary Material online; Egusa and Nakajima 1981; Zhou et al. 1998; Xie et al. 2000; Liu et al. 2011). BLAST analysis showed that 18S rRNA sequence of the isolated species was 99% identical to that of the *T. kitauei*-China (HQ115585) and *T. kitauei*-Korea (HM624024; supplementary table S2, Supplementary Material online). Furthermore, the phylogenetic analysis showed that the parasite from common carp in this study is clustered with *T. kitauei*-China (HQ115585) and *T. kitauei*-Korea (HM624024) with 100% bootstrap confidence values (supplementary fig. S1*C*, Supplementary Material online). These findings, combining with the fact that this parasite showed the same host and tissue specificity as *T. kitauei*, confirmed our isolate as *T. kitauei*.

### Genome Sequencing and Assembly

To optimize genome assembly, two next-generation sequencing platforms, Roche 454 and Illumina, were used to sequence the genome of *T. kitauei*. We generated 1.48 Gb (∼8 × genome coverage; 8-kb library with a Q20 percentage of 86.3% and 20-kb library with a Q20 percentage of 90.5%) from the Roche 454 sequencing and 5.5 Gb (∼29 × genome coverage; 3-kb mate-paired library with a Q20 percentage of 75.4%) and 2.5 Gb (∼13 × genome coverage; 500-bp paired-end library with a Q20 percentage of 80.1%) from Illumina sequencing. A hybrid genome assembly of 454 and Illumina sequences was conducted by first assembling the Illumina mate-pair and paired-end data with SOAPdenovo, followed by fragmentation of the SOAPdenovo assembled contigs and assembly with the Roche 454 data using Newbler. This approach improves the performance of Newbler with Roche 454 data by correcting the homopolymer and insertion/deletion errors common to Roche 454 sequencing. Potential contamination of the parasite samples with carp DNA was excluded in four ways: 1) During sample collection, the carp intestinal tissue was carefully removed from the cysts, and the collected *T. kitauei* spores were repeatedly washed; 2) The purity of the *T. kitauei* DNA was checked by PCR with carp-specific primers, and no carp DNA was detected; 3) In the bioinformatics analysis, assembled scaffolds showing high similarity (*E* value ≤ 1 e^−^^20^) to fish and bacterial sequences in GenBank were excluded; 4) A mean ± standard deviation (*n* = 3) approach was used to exclude potential carp scaffolds based on the fact that the mean GC content of *T. kitauei* (∼0.29; supplementary fig. S2, Supplementary Material online) is lower than that of fish. After the filtering process, 150.7 Mb were assembled into 5,610 scaffolds (N50 = 170 kb), and over 90% of the genome assembly was included in 1,336 scaffolds.

The genome size of *T. kitauei* was estimated based on 17-mer analysis (Kurtz et al. 2008; Wang et al. 2011; supplementary fig. S3, Supplementary Material online). To further eliminate contaminating fish and bacterial reads, only the reads mapped to the draft assembly (∼1.3 Gb) were used. The peak of 17-mer frequency (M) correlates with sequencing depth (N), read length (L), and k-mer length (K), with the relationship expressed as M = N × (L − K + 1)/L. The genome size of *T. kitauei* was inferred to be 188.5 Mb, meaning that 80% of the genome sequences were present in our draft assembly of the *T. kitauei* genome. The loss of the remaining 20% of the genome sequences may be due to the presence of repeat sequences in the genome and/or the limitations of next-generation sequencing technologies.

### Repeat Sequences

The assembled *T. kitauei* genome was found to contain approximately 13.8% interspersed repeat sequences (supplementary table S3, Supplementary Material online), and this number is likely underestimated owing to the limitations of genome sequencing strategies. Among the interspersed repeat sequences, approximately 7% were transposable DNA elements, including hAT-Charlie (2.3%) and TcMar-Tigger (0.2%); and some (0.07%) were long interspersed nuclear elements, while no short interspersed nuclear elements were observed. Moreover, a large number of DNA transposons and retrotransposons were found in the *T. kitauei* genome (supplementary table S4, Supplementary Material online). These elements can lead to increased expression if inserted in the vicinity of virulence-related genes (Faust and Guillen 2012). Proteins encoded by the transposons and retrotransposons fell into several families, including endonucleases, reverse transcriptases, transposases, and integrases (supplementary table S4, Supplementary Material online). One of these transposons encodes a retroviral aspartyl protease belonging to the expansive retroviral-like protease family, which may be involved in processing of polyproteins in *T. kitauei*.

### Gene Models

With the combination of four gene-scanning programs, 16,638 protein-coding genes were annotated in the 150. 7-Mb draft genome assembly. Transcriptome data were also collected to verify the gene annotations of the *T. kitauei* genome. About 8,659 predicted genes, accounting for 51.5% of the total predicted genes (16,638 protein-coding genes), were expressed in the myxospores of *T. kitauei*. The numbers of genes with RPKM values in the range of 10–100, 100–1,000, and over 1,000 were 1,817, 715, and 121, respectively. Thereafter, we then compared the genome features of *T. kitauei* to those of the two closely related free-living cnidarian species *H. magnipapillata* and *N. vectensis* (table 1). Although the estimated genome size of *T. kitauei* is approximately half of that of *N. vectensis* and one-fifth of that of *H. magnipapillata*, the gene numbers among the three species were very similar. The percentages of coding sequences, gene density, and average exon size of *T. kitauei* were much higher than those of *N. vectensis* and *H. magnipapillata* (table 1). Similar to the ciliate *Paramecium tetraurelia* (Aury et al. 2006), *T. kitauei* has relatively short introns, with the majority in the range of 20–25 bp (supplementary fig. S4, Supplementary Material online). The short introns may not allow alternative splicing (Aury et al. 2006). However, the benefits, if any, are unclear.

**Table 1:** Summary of Genomic Features of *Thelohanellus kitauei* and Two Free-Living Cnidarians

Genome features	Parasitic	Free-Living	
	*Thelohanellus kitauei*	*Hydra magnipapillata* (Chapman et al. 2010)	*Nematostella vectensis* (Putnam et al. 2007)
Total genome size (Mb)	150.7	852.2	356.6
Total coding size (Mb)	20	21.5	30.5
Total coding ratio (%)	13.3	2.5	8.6
Gene number	16,638	17,918	26,676
Average gene length (kb)	1,210	14,568	5,869
Gene density (genes per Mb)	110	21	75
Average coding sequence size (kb)	717	1,267	1,223
Average exon number	3	6	5
Average exon size (bp)	235	218	208
Average intron size (bp)	240	2,653	1,122
Total GC content (%)	37.5	27.8	40.6
GC content in coding region (%)	39.0	33.5	46.5

**Fig. 1.—:**
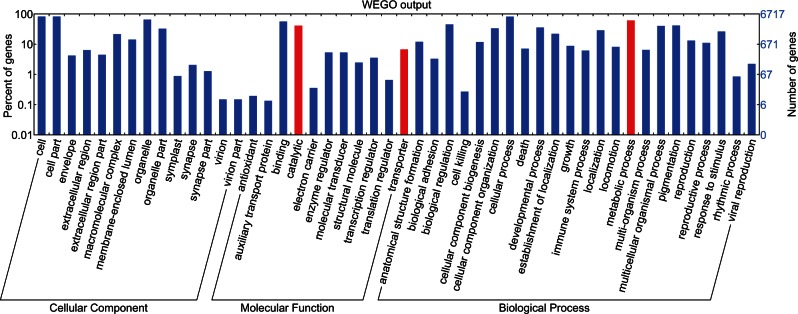
Overall function catalogs of *T. kitauei* gene annotation. Function catalogs described in this study are highlighted in red.

Approximately 55% of the putative genes have homologs in the NCBI database and >3,500 gene products could be assigned to gene ontology terms. As shown in figure 1, a large number of *T. kitauei* genes appear to be involved in multicellular organismal processes. In addition, KEGG pathway analysis revealed that *T. kitauei* carries genes encoding components of the Hedgehog, Jak-STAT, Notch, TGF-β, mTOR, and Wnt pathways (supplementary table S5, Supplementary Material online), which are signaling pathways present in multicellular Metazoa but not in fungi or choanoflagellates. Together, these results are coincident with the fact that *T. kitauei* is a multicellular metazoan.

### Proteases and Protease Inhibitors As Main Enzymes for Nutrient Digestion

**Fig. 2.—:**
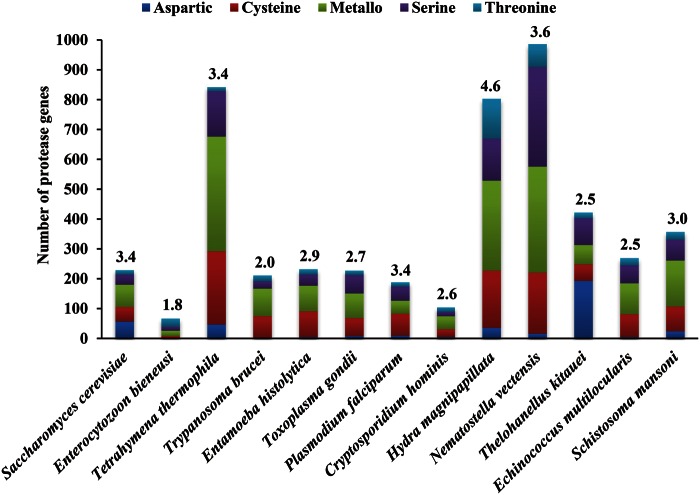
Comparison of proteases in *T. kitauei* and other organisms. Values above column are the percentage of the proteases within the total proteome of that species.

We identified 422 protease (including 34 secreted) homologues in *T. kitauei* through the MEROPS database, constituting around 2.5% of the proteome (fig. 2 and supplementary tables S6–S8, Supplementary Material online). The number of proteases in *T. kitauei* is much greater than the number of putative lipid (22 including 1 secreted) and carbohydrate (44 including 4 secreted) digestive enzymes. The number of digestive enzymes in *T. kitauei* was considerably lower than that in the two free-living relatives, with the number in *T. kitauei* representing 42.8%/52.6%, 16.3%/37.3%, and 8.5%/19.4% of the numbers in *N. vectensis*/*H. magnipapillata*, for proteases, lipid and carbohydrate digestive enzymes, respectively. As noted, the scale of enzyme number reduction in *T. kitauei* as compared with the free-living relatives was lower for proteases than that of the lipid and carbohydrate digestive enzymes, suggesting that proteases are the main enzymes involved in nutrient digestion (supplementary tables S7–S12, Supplementary Material online).

Compared with 11 other free-living and parasitic fungi, protozoans, and metazoans (fig. 2 and supplementary table S6, Supplementary Material online), the distribution of proteases in *T. kitauei* is clearly distinct: *T. kitauei* has a relatively large proportion of aspartic proteases (46% vs. 0−25%) but small proportion of cysteine proteases (13% vs. 12−38%) and metalloproteases (16% vs. 27−46%; fig. 2). Aspartic proteases subfamily A11X (the transposon endopeptidases) and A33 (skin-specific retroviral-like aspartic protease) are also expanded in *T. kitauei* relative to *H. magnipapillata* and *N. vectensis* (supplementary table S7, Supplementary Material online). These proteins are highly related to hemoglobin catabolism, transposon multiplication, and processing of profilaggrin (Matsui et al. 2011) and therefore likely account for the significant decrease in red blood cells in *T. kitauei*-infected carp (Wang et al. 2005). The proportion of astacin metalloproteases in *T. kitauei* (0.2% of total proteases) were much lower than those in two free-living cnidirians (8.5% and 8.9% of total proteases in *H. magnipapillata* and *N. vectensis*, respectively; supplementary table S7, Supplementary Material online). As astacin metalloproteases were reported to have the function for degradation of hydra extracellular matrix (ECM; Sarras et al. 2002), which might be beneficial for a myxozoan parasite, the low abundance of astacin metalloproteases may suggest that *T. kitauei* may have distinct ECM degradation pathway that may involve other metalloproteases or members of serine and cysteine proteinases (Lu et al. 2011).Other abundant protease families in at least one of two free-living cnidarians, such as the ubiquitin-specific proteases (C19 family), lysostaphin-type enzymes (M23B subfamily), and γ-glutamyl transferases (T3 family), were also largely reduced in *T. kitauei* (supplementary table S7, Supplementary Material online). Furthermore, around 40% of the protease families that were present in the two free-living cnidarians are absent in *T. kitauei* (supplementary table S7, Supplementary Material online). Taken together, these observations indicated a different life styles of *T. kitauei* from its free-living relatives.

Relative to the number of proteases, there were more (206) putative protease inhibitors in *T. kitauei* than in the two free-living cnidarians (94–164), although the diversity (8 vs. 15–18 families) was lower in *T. kitauei* (supplementary table S13, Supplementary Material online). Moreover, the serpin family (I04), which contains inhibitors of serine and cysteine endopeptidases, was greatly expanded in *T. kitauei* (182 vs. <5 in *H. magnipapillata* and *N. vectensis*).

Thirty-four proteases contain signal peptides (potential secretory pathway-targeted proteins, supplementary table S8, Supplementary Material online). Within them, 5 of the 19 proteases in the C1A subfamily and 2 of the 5 kexin-type peptidases in the S8B subfamily are highly expressed (RPKM values ≥ 100) in the myxospore stage of *T. kitauei*. Nineteen protease inhibitors were putatively secretory in *T. kitauei*, with the majority (14) as serine and cysteine protease inhibitors belonging to the serpin, serine carboxypeptidase Y (I51), and Kunitz-type (I02) families, which may regulate the activities of host-derived serine and cysteine proteases. Notably, two of the secretory protease inhibitors from the I25A and I51 subfamily are highly expressed in the myxospore stage. Proteases and their inhibitors have been reported as important factors for invasion and immune evasion of parasites (Toubarro et al. 2010; Doyle et al. 2011; Faria et al. 2011; Alam et al. 2013). The highly expressed (RPKM values ≥ 100) secreted proteases and inhibitors observed in *T. kitauei* myxospore stage suggest their putative involvement in these functions (supplementary table S8, Supplementary Material online).

### Nutrient Absorption Mainly Depends on Endocytosis and Secondary Carriers

**Fig. 3.—:**
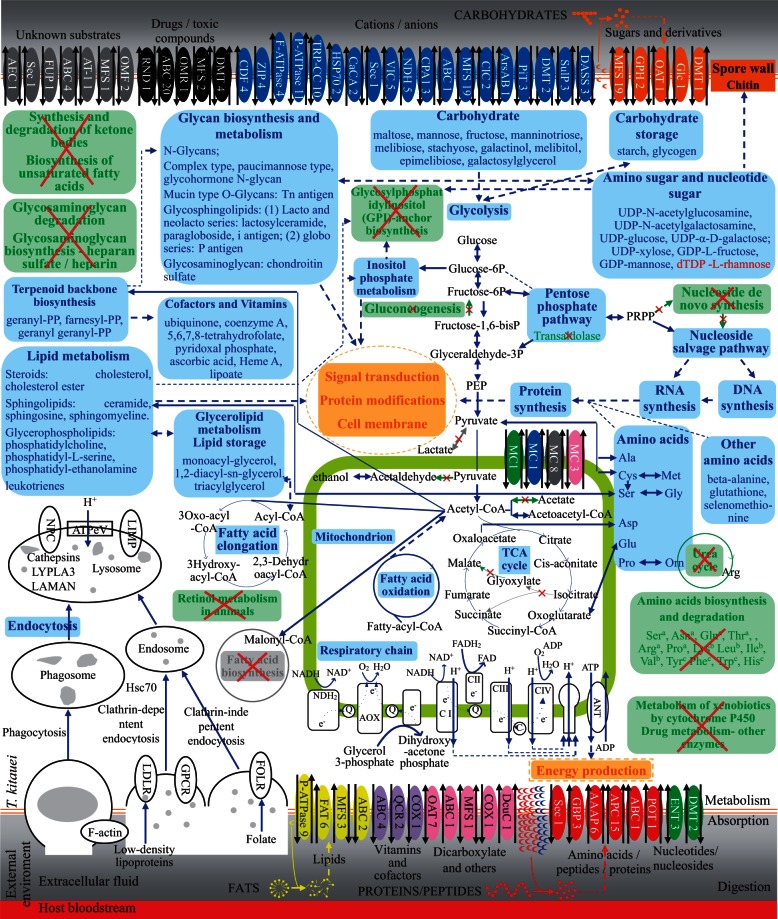
An overview of nutrient digestion, absorption and metabolism in *T. kitauei*, as deduced from genome sequence analysis. The color-coding in the secretory digestive enzymes of *T. kitauei*: red, proteases; blue, proline iminopeptides; orange, carbohydrate-binding module family proteins and glycoside hydrolases; yellow, lipase. Transporters are grouped by substrate specificity: red, amino acids/peptides/proteins; orange, sugars and derivative; yellow, lipids; green, nucleotides/nucleosides; purple, vitamins and cofactors; pink, dicarboxylates and others organic; blue, cations and anions; black, drugs/toxic compounds; gray, unknown substrates; numbers indicated the number of predicted transporters of each type. The color-coding in metabolism of *T. kitauei* indicates: blue, enzymes or pathways present in all three species, *T. kitauei*, *H. magnipapillata*, and *N. vectensis*; green with a cross, enzymes or pathways present in at least one of the two free-living cnidarians but absent in *T. kitauei*; gray with a cross, enzymes or pathways absent in all three species; red, enzymes or pathways present only in *T. kitauei*; orange, putative functions. Amino acids that can be de novo synthesized and catabolized (a) or partially synthesized and catabolized (b) or only catabolized (c) by the two free-living cnidarians. Unbroken arrows indicate direct steps in a pathway, and broken arrows indicate multiple steps in a pathway not shown. Amino acids are indicated in the three letter code. AAAP, amino acid/auxin permease; APC, amino acid-polyamine-organocation family; ArsAB, arsenite-antimonite efflux family; ABC, ATP-binding cassette superfamily; AT-1, autotransporter-1; AEC, auxin efflux carrier; DcuC, C4-dicarboxylate uptake C; CaCA, Ca^2+^:cation antiporter family; Hsp70, cation channel-forming heat shock protein-70 family; CDF, cation diffusion facilitator; ClC, chloride carrier/channel family; DASS, divalent anion: Na^+^ symporter; DMT, drug/metabolite transporter; ENT, equilibrative nucleoside transporter; GBP, general bacterial porin; Sec, general secretory pathway family; GPH, glycoside-pentoside-hexuronide: cation symporter; F-ATPase, H^+^- or Na^+^-translocating F-type, V-type and A-type ATPase; PiT, inorganic phosphate transporter; MFS, major facilitator superfamily; MC, mitochondrial carrier; CPA1, monovalent cation: proton antiporter-1; OAT, organo anion transporter; OMF, outer membrane factor; FUP, outer membrane fimbrial usher porin family; OMR, outer membrane receptor; FAT, proposed fatty acid transporter; POT, proton-dependent oligopeptide transporter; COX, proton-translocating cytochrome oxidase; NDH, proton-translocating NADH dehydrogenase; QCR, proton-translocating quinol: cytochrome c reductase; Glc, PTS glucose-glucoside; P-ATPase, P-type ATPase; RND, resistance-nodulation-cell division; SulP, sulfate permease; TRP-CC, transient receptor potential Ca^2+^ channel; VIC, voltage-gated ion channel; ZIP, zinc (Zn^2+^)-iron (Fe^2+^) permease; PEP, phosphoenolpyruvic acid; NADH, nicotinamide adenine dinucleotide; Q, quinine; AOX, alternative oxidase; CI to CIV, complex I to complex IV; C, cytochrome c; ANT, adenine nucleotide translocator; FAD, flavin adenine dinucleotide; FADH_2_, reduced FAD; ADP, adenosine diphosphate; FOLR, folate receptor; GPCR, G protein-coupled receptors; LDLR, low-density lipoprotein receptor; Hsc70, heat shock 70 kDa protein 1/8; ATPev, V-type H^+^-transporting ATPase; LIMP, lysosomal integral membrane protein; NPC, niemann-pick C protein; LYPLA3, lysophospholipase III; LAMAN, lysosomal alpha-mannosidase; F-actin, actin beta/gamma 1; TCA, tricarboxylic acid.

Endocytosis (classified as clathrin-mediated endocytosis, clathrin-independent endocytosis, and phagocytosis) has been confirmed to be central in the pathogenesis of many parasites, including *Leishmania* spp., *Giardia lamblia*, trypanosomatids, *Entamoeba histolytica*, and *Toxoplasma gondii* (Robibaro et al. 2001; Lopez-Soto et al. 2009; Rivero et al. 2010; Subramanya and Mensa-Wilmot 2010; Andrade-Neto et al. 2011; Rivero et al. 2011). Like the two free-living relatives, *T. kitauei* has most of endocytosis-related genes (134 including 72 phagosome-related genes; fig. 3 and supplementary fig. S5*A*, Supplementary Material online). Although failed to map to KEGG endocytosis pathway, the extensive collection of genes in low-density lipoprotein receptor family (122 genes) detected in *T. kitauei* suggests that low-density lipoprotein receptor-mediated endocytosis (a type of clathrin-dependent pathway) may play an important role in *T. kitauei* uptake of fish lipids (supplementary fig. S5*A*1 and table S14, Supplementary Material online). Although caveolin, the central component of caveolae-dependent endocytosis (a type of clathrin-independent pathway), was not detected in both *T. kitauei* and the two relatives, folate receptors, which are alternatively related to the clathrin-independent pathway, were identified. In addition, actin β/γ 1 is expressed at relatively high level (RPKM = 1,7702.82) in the transcriptome of myxospore-stage *T. kitauei*, and might be essential for phagocytosis (supplementary fig. S5*A*2, Supplementary Material online; Doherty and McMahon 2009; Mooren et al. 2012). Moreover, although *T. kitauei* has fewer lysosomal membrane proteins and acid hydrolases compared with its two free-living relatives (supplementary fig. S5*A*3, Supplementary Material online), its CD63 antigen (RPKMs of 2 of 3 genes are 2,395.23 and 2,638.33), Niemann-Pick C1 protein 1 (RPKMs of 2 of 7 genes are 355.94 and 589.40), cysteine protease cathepsin L (RPKMs of 5 of 20 genes are ranged from 113.45–4,977.53), and lipase LYPLA3 (RPKM of 1 of 3 genes is 610.83) are highly expressed in the myxospore stage. These results indicate that *T. kitauei* mainly carries out endocytosis, especially low-density lipoprotein receptor-mediated endocytosis, to acquire main nutrients such as lipids (supplementary fig. S5*A*, Supplementary Material online).

**Fig. 4.—:**
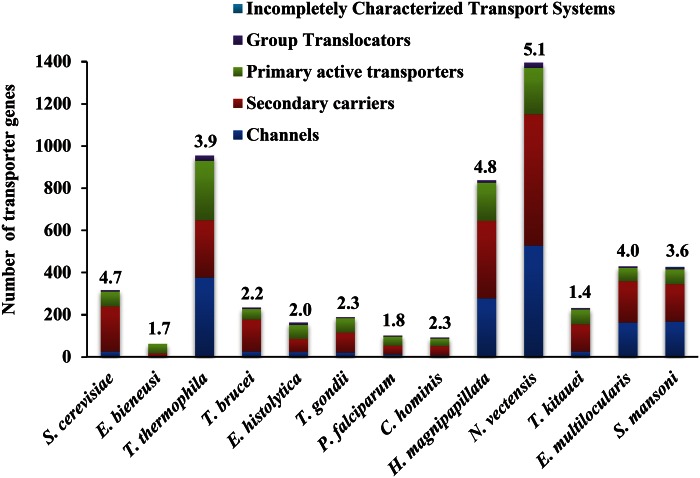
Comparison of transporters of *T. kitauei* and other organisms. Values above column are the percentage of the transporters within the total proteome of that species.

In comparison with the two free-living cnidarians, *T. kitauei* has a limited repertoire of membrane transporters (231 transport proteins in 38 different transporter superfamilies/families vs. 837−1,395 transporters in 60−73 families; figs. 3 and 4). In addition, there are fewer energy-independent channel proteins in *T. kitauei* (11% of the total transporters in *T. kitauei* vs. ∼33% in the two free-living cnidarians). *Thelohanellus kitauei* seems to rely primarily on secondary carriers (56%) and to a lesser degree on primary active transporters (30%) (fig. 4). The secondary carriers of *T. kitauei* fall into two main superfamilies, the major facilitator (24% of the total transporters) and the amino acid/polyamine/organocation superfamilies (10%) (supplementary table S15, Supplementary Material online), which together are involved in the utilization of carbohydrates, phosphates, and amino acids (Wong et al. 2012; Yan 2013). On the other side, the primary active transporters of *T. kitauei* are mainly composed of ATP-binding-cassette transporters (14%), which involve in exporting drug (Leprohon et al. 2011), and P-type ATPases (9%), which transport phospholipids, sodium, potassium, and calcium (Kuhlbrandt 2004). Nearly one-third of the cation transporters are involved in Ca^2+^ transport, and the majority (∼68%) of the putative anion transporters are involved in phosphate transport (supplementary table S15, Supplementary Material online). Therefore, the low blood phosphorus and calcium levels observed in *T. kitauei* infected carp might be attributed to the powerful capability of *T. kitauei* in transporting host calcium and phosphate (Wang et al. 2005).

### Anabolism Rather than Catabolism

**Fig. 5.—:**
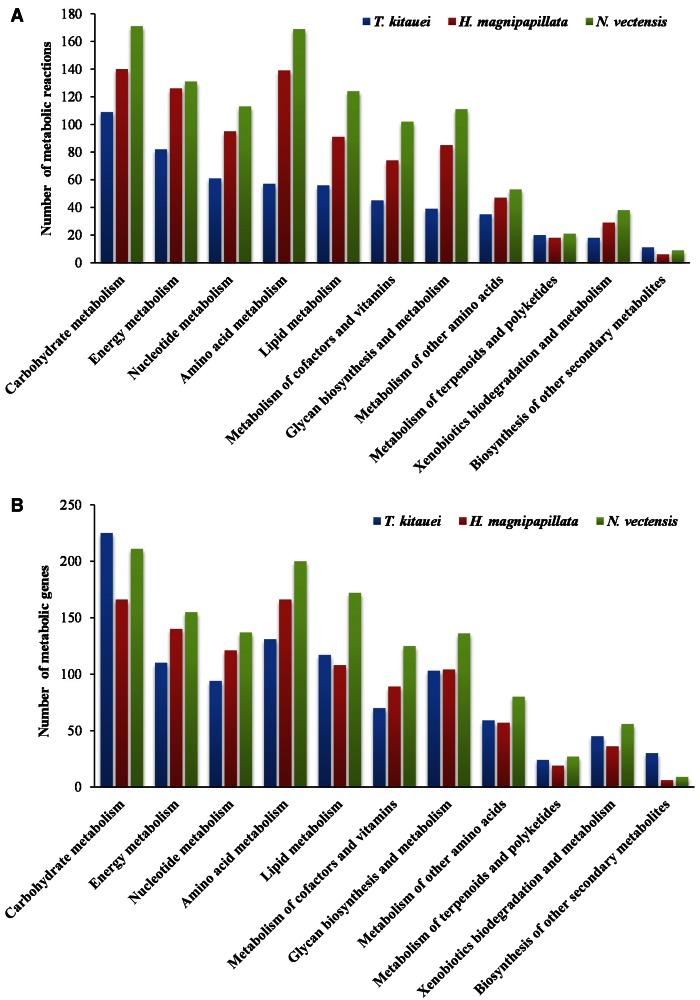
Number of metabolic reactions (*A*) and number of metabolic genes (*B*) in comparison between *T. kitauei* and two free-living cnidarias.

To date, the metabolic pathways of myxozoans have not been well characterized. In this study, comprehensive, genome-wide metabolic pathway reconstruction for a myxozoan was firstly conducted. Compared with the two free-living relatives, *T. kitauei* genome revealed extensive reductions in overall metabolic capacity, with fewer genes involved in the eight primary metabolic processes, that is, carbohydrate, energy, nucleotide, amino acid, lipid, cofactor/vitamin, glycan, and other amino acid metabolism (figs. 3 and 5 and supplementary fig. S5*B–K*, Supplementary Material online).

Anaerobic fermentation plays an important role in the energy metabolism of many parasites, such as *E. histolytica*, *Plasmodium falciparum*, and tapeworms (Vanacova et al. 2003; Painter et al. 2007; Tsai et al. 2013). Both *T. kitauei* and its free-living relatives are unlikely to be able to produce lactate, acetaldehyde, and acetate through fermentation, as they lack the requisite lactate dehydrogenase, pyruvate decarboxylase, and acetyl-CoA synthetase (ADP-forming; fig. 3 and supplementary fig. S5*B*1, Supplementary Material online; Sanchez and Muller 1996; Zhao et al. 2013; Mithran et al. 2014). Moreover, unlike the two free-living relatives, *T. kitauei* lacks malate dehydrogenase (EC 1.1.1.40) for conversion of malate to pyruvate , as well as the propionyl-CoA cycle which converts succinate to propionate, suggesting a less importance of the anaerobic malate dismutation pathway producing succinate in *T. kitauei* (supplementary fig. S5*B*2 and 3, Supplementary Material online; Muller et al. 2012; Mentel et al. 2014). Taken together, these results imply that *T. kitauei* does not rely much on anaerobic fermentation. On the other hand*,* some enzymes related to the tricarboxylic acid cycle (RPKMs of 10 genes were higher than 100, and RPKMs of 16 genes were higher than 10, out of 32 genes) and oxidative phosphorylation (RPKMs of 5 genes were higher than 100, and RPKMs of 27 genes were higher than 10, out of 44 genes) were highly expressed in the myxospore stage of *T. kitauei* (fig. 3 and supplementary fig. S5*B*4 and *C*1, Supplementary Material online) further suggesting that *T. kitauei* may have greater metabolic dependency on respiration than on fermentation.

Compared with the two free-living cnidarians, *T. kitauei* has a reduced ability to de novo synthesize and hydrolyze amino acids (fig. 3 and supplementary fig. S5*D*, Supplementary Material online), which is similar to *Schistosoma mansoni* and tapeworms (Berriman et al. 2009; Tsai et al. 2013). *Thelohanellus kitauei* seems to only be able to de novo synthesize or hydrolyze a few amino acids including alanine, aspartate, glutamate, glycine, cysteine, and methionine (fig. 3 and supplementary fig. S5*D*, Supplementary Material online). The polyamines and their derivatives mediate a variety of important functions and are therefore essential to cell growth and proliferation (Birkholtz et al. 2011). *Thelohanellus kitauei* may use ornithine to synthesize polyamines, as the enzyme (ornithine decarboxylase) that converts ornithine to the polyamine putrescine was highly expressed (RPKM of 1 of 2 genes is 5,246.84) in the myxospore stage of *T. kitauei*. Other amino acids that can be de novo synthesized (serine, asparagine, glutamine, threonine, arginine, and proline) or partially synthesized (lysine, leucine, isoleucine, and valine) and catabolized by the two free-living cnidarians are unlikely synthesized or catabolized in *T. kitauei* (supplementary fig. S5*D*, Supplementary Material online)*.*

Although both *T. kitauei* and the two free-living relatives lack the pathways for fatty acid biosynthesis, they are capable of fatty acid elongation (fig. 3 and supplementary fig. S5*E*, Supplementary Material online). Unlike the two free-living relatives, *T. kitauei* has an incomplete β-oxidation pathway in that it lacks dehydrogenases specific for either short- or medium-chain acyl-CoA (supplementary fig. S5*E*1, Supplementary Material online), suggesting that the lipid metabolism in *T. kitauei* inclined to anabolism rather than catabolism.

The reduced ability to hydrolyze fatty acids and amino acids, and the completeness of key carbon metabolism pathways (glycolysis and the tricarboxylic acid cycle) suggest that carbohydrates are the primary energy source for *T. kitauei.* The two free-living cnidarians have the complete pentose phosphate pathway, whereas *T. kitauei* appears to have a transaldolase-deficient pentose phosphate pathway, like in *P. falciparum* (Gardner et al. 2002; supplementary fig. S5*B*5, Supplementary Material online). Nevertheless, *T. kitauei* seems to utilize an alternative pathway that contains three enzymes, namely phosphofructokinase, transketolase, and aldolase for hexose-pentose interconversion, which maintains cytosolic NADP^+^/NADPH and H^+^ balance. Notably, ribose-phosphate pyrophosphokinase, which converts d-ribose-5′-phosphate to phosphoribosyl pyrophosphate, is highly expressed (RPKMs of 3 genes are 297.03, 592.19, and 968.95, respectively) in the myxospore stage of *T. kitauei*. Adenosine monophospates (AMP) can be converted to high energy molecule adenosine triphosphate (ATP) and its cyclic structure cyclic AMP plays an important role in intracellular signaling. The product phosphoribosyl pyrophosphate seems to mainly enter the adenine salvage pathway to form AMP, due to the absence of enzymes (amidophosphoribosyl transferase, uridine monophosphate synthetase, and ATP phosphoribosyltransferase) for either de novo nucleotide synthesis or histidine metabolism, and the presence of adenine phosphoribosyltransferase in *T. kitauei* (supplementary fig. S5*B*5 and *F*1, Supplementary Material online). The genomes of the two free-living cnidarians indicate that they can synthesize all glycolytic metabolites by gluconeogenesis. *Thelohanellus kitauei*, however, lacks fructose-bisphosphatase (supplementary fig. S5*B*1, Supplementary Material online), implying the absence of gluconeogenesis and the inability to convert fatty acids and amino acids into glucose. Actually, the fructose-bisphosphatase was also absent in other intestinal parasites such as *G. lamblia*, *E. histolytica*, *Cryptosporidium paryum*, and *Cryptosporidium hominis* (http://www.genome.jp/kegg-bin/get_htext?htext=br08601_map00010.keg&hier=5). Taken together, the absence of gluconeogenesis, high proportion of sugar transporters (10% of total transporters, supplementary table S15, Supplementary Material online), and the high proportion of glycosyltransferases (70.8% of total carbohydrate-active enzymes vs. 27.5–37.8% of those in *N. vectensis* and *H. magnipapillata*, supplementary table S11, Supplementary Material online) may underlie the evolution of *T. kitauei*’s ability to more efficiently harvest carbohydrates (mainly glucose) from the host.

The genomes of the two nonparasitic relatives suggest that they are capable of synthesizing and hydrolyzing chitin. Although failed to map to KEGG metabolic pathways, ten genes encoding cellulose/chitin synthases were annotated in *T. kitauei* using the dbCAN Web server (supplementary fig. S5*B*6 and table S11, Supplementary Material online). However, *T. kitauei* lacks both chitinases and chitosanases (supplementary table S11, Supplementary Material online). Therefore, *T. kitauei* either degrade chitin through endoglucanases, endo-β-*N*-acetylglucosaminidases, or other enzymes containing chitin/chitopentaose-binding modules that were identified in *T. kitauei* genome (supplementary table S11, Supplementary Material online). Alternatively, it is not impossible that *T. kitauei* does not have the capability for chitin degradation. Further investigations are necessary to uncover the detailed model of chitin metabolism in *T. kitauei*.

*Thelohanellus kitauei* seems to have lost the ATP-expensive pathways for de novo biosynthesis of inosine 5′-phosphate and uridine 5′-phosphate. Therefore, it must rely on salvage pathways, which is similar to other parasites such as *E. histolytica*, *G. lamblia*, and *Trichomonas vaginalis* (supplementary fig. S5*F*, Supplementary Material online; Abrahamsen et al. 2004; Loftus et al. 2005). Genes encoding components of the purine ribonucleotides or pyrimidine ribonucleotides interconversion pathways were identified in the genome of *T. kitauei*, suggesting that it can generate all necessary nucleotides from individual sources—purines or pyrimidines (supplementary fig. S5*F*, Supplementary Material online). In contrast to the two free-living cnidarians, *T. kitauei* lacks the enzymes for degradation of free purine and pyrimidine bases.

The reduced level of metabolic complexity observed in *T. kitauei* is a convergent trait among endoparasites (Nerima et al. 2010). The lack of de novo synthesis of fatty acids, nucleotides, and many of the amino acids as well as the reduced capacity for hydrolysis of fatty acids, nucleosides, and amino acids suggest that these molecules are mainly directed to anabolic rather than catabolic processes and that carbohydrates are the primary energy source. Also, the lack of gluconeogenesis implies the high reliance of carbohydrates (mainly glucose) from the host. All together, these facts suggest that *T. kitauei* scavenges nutrients from its host to compensate for its streamlined metabolic capabilities. The high nutrients (especially carbohydrates) requirement and the predominant aerobic respiration are consistent with the parasitic site of *T. kitauei*, that is, the loose connective tissue of the intestinal submucosa, where a high abundance of capillaries may supply sufficient oxygen and nutrients, especially glucose.

### Control Strategy for *T. kitauei*

Some antiprotozoan drugs have shown efficacy against myxosporean infections, but these compounds either are toxic to fish or have shown limited efficacy or poor palatability (Molnar 1993; Rhee et al. 1993; le Gouvello et al. 1999; Athanassopoulou et al. 2004; Karagouni et al. 2005; Yonar and Yonar 2010; Jones et al. 2012; Manning et al. 2013). Also, many myxozoans elicit little or no host immune response in fish (Sitja-Bobadilla 2008; Gomez et al. 2014), leading to the absence of effective antimyxozoan immunoprophylactic agents till now. Therefore, novel targets and compounds are urgently needed.

The myxospore stage is tied with disease processes of *T. kitauei* in its carp host. To identify potential drug targets, we first selected the 2,653 most highly expressed genes (RPKMs ≥ 10) out of 8,659 expressed ones in the *T. kitauei* spore transcriptome. The putative products of these genes were searched against known drug target proteins in the ChEMBL database (https://www.ebi.ac.uk/chembl/), and 513 were found to be homologs of existing drug targets (BLAST *E* value ≤1 e ^−^^10^; supplementary table S16, Supplementary Material online).

Nutritional and metabolic pathways have traditionally provided attractive targets for drug development. For example, the widely used quinoline-containing antimalarial drugs inhibit endocytosis (Roberts et al. 2008; Dunmore et al. 2013); vaccines against helminth parasites target digestive proteases (Pearson et al. 2010); several promising chemotherapeutic targets have been identified in the lipid synthetic and salvage pathways of apicomplexan parasites (Coppens 2013); and therapeutic approaches are being developed that exploit auxotrophies and metabolic defects of *T. gondii* (Coppens 2014). Therefore, targets related to nutritional and metabolic pathways are of particular interest in our study. Among the 513 drug targets we identified, 220 are related to nutrient digestion (27), absorption (68), and metabolism (125). Genes of the 513 drug targets were then compared with the genome of carp to avoid potential drug toxicity arising from chemical cross-reactivity between the orthologous parasite and host targets (Swain et al. 2011; http://zfgenomics.zfscreens.com/sub/carp). Fourty-seven *T. kitauei*-specific target genes that lack orthologs in the host were selected (BLAST *E* value >1 e ^−^^3^; supplementary table S16, Supplementary Material online), with 23 of them related to nutritional and metabolic pathways (table 2). Six *T. kitauei*-specific targets are related to endocytosis, including two low-density lipoprotein receptor-related proteins, and two sortilins that are involved in the regulation of both apolipoprotein B secretion and low-density lipoprotein catabolism (Strong et al. 2012). Four putative *T. kitauei*-specific targets are related to lipid metabolism. Other *T. kitauei*-specific targets related to transport (3) and metabolism of carbohydrates (4), cofactors (3), glycan (1), nucleotides (1), and amino acids (1) (table 2). Homologues of targets for treatment of distantly related protozoan and fungal parasites such as *Leishmania* spp., *Plasmodium* spp., and *Aspergillus fumigatus* were included in the list of *T. kitauei*-specific targets (table 2), suggesting that some parasitic mechanisms and metabolic pathways are shared by even evolutionarily distant parasites and compound libraries developed against targets in one parasite may contain leads to pursue for chemotherapy of other infections (Renslo and McKerrow 2006). Moreover, the list of *T. kitauei*-specific targets includes several homologues of targets for cancer chemotherapy, including arachidonate 5-lipoxygenase, 17 beta-estradiol 17-dehydrogenase, diacylglycerol kinase, 2,3-bisphosphoglycerate-independent phosphoglycerate mutase, and ectonucleoside triphosphate diphosphodydrolase (table 2), implying some consistency in the challenges of inhibiting cancer tumors and parasitic infestion (Tsai et al. 2013). Actually, the anthelminthic medicines niclosamine, mebendazole, and albendazole have already been shown to inhibit cancer growth (Tsai et al. 2013).

**Table 2:** Promising *Thelohanellus kitauei*-Specific Drug Targets (23) Relating to Nutrient Process

Gene ID	Predicted Protein	Function Category	Previous Indications As Drug Target
evm.model.scaffold04826.5	Peptidyl-prolylcis-trans isomerase FKBP4	Transport	Antiparasitic targets (Ibrahim and Nishikawa 2012)
evm.model.scaffold05481.72	Vesicular glutamate transporter 3		
evm.model.scaffold02466.3			
evm.model.scaffold00373.8	Integrin beta 2	Endocytosis	Antiinflammatory target (de Gaetano et al. 2013)
evm.model.scaffold04701.1	Heterogeneous nuclear ribonucleoprotein K		Anticancer target (Benelli et al. 2009)
evm.model.scaffold01071.3	Sortilin		Target for Alzheimer's disease (Caglayan et al. 2014)
evm.model.scaffold01071.5			
evm.model.scaffold05633.24	Low-density lipoprotein-related protein 2		Target for Alzheimer's disease (Martiskainen et al. 2013)
evm.model.scaffold04423.69			
evm.model.scaffold01248.6	Arachidonate 5-lipoxygenase	Lipid metabolism	Anticancer target (Chen et al. 2009)
evm.model.scaffold04176.12	17 beta-estradiol 17-dehydrogenase		Anticancer target (Szajnik et al. 2012)
evm.model.scaffold00813.1	Diacylglycerol kinase (ATP dependent)		Targets for cancer (Dominguez et al. 2013)
evm.model.scaffold04610.1	Phospholipase D3		Target in disease settings (Huang and Frohman 2007)
evm.model.scaffold05340.25	Oligosaccharyltransferase complex subunit beta	Glycan biosynthesis	Target for *Aspergillus fumigatus* (Li et al. 2011)
evm.model.scaffold00661.36	2,3-bisphosphoglycerate-independent phosphoglyceratemutase	Carbohydrate metabolism	Anticancer (Ren et al. 2010)/antifilarial (Singh et al. 2013) target
evm.model.scaffold03068.51	Inositol-tetrakisphosphate 1-kinase		Antimalarial target (Stritzke et al. 2012)
evm.model.scaffold01538.6	Inositol-1,4,5-trisphosphate 5-phosphatase		Antimalarial target (Stritzke et al. 2012)
evm.model.scaffold01687.1	Ribokinase		Antibacterial target (Desroy et al. 2009)
evm.model.scaffold04623.113	Pyrazinamidase/nicotinamidase PNCA	Cofactor metabolism	Target for *Leishmania* (Gazanion et al. 2011)
evm.model.scaffold02481.37			
evm.model.scaffold03983.31	Thiamine pyrophosphokinase		Antimalarial target (Chan et al. 2013)
evm.model.scaffold04174.4	Ectonucleoside triphosphate diphosphohydrolase 4	Nucleotide metabolism	Anticancer target (Tada et al. 2011)
evm.model.scaffold02448.28	Glutathione S-transferase	Other amino acid metabolism	Antimesothelioma (De Luca et al. 2013) /antimalarial (Harwaldt et al. 2002) target

## Conclusion

The characterization of the first genome of a myxozoan will provide a platform for exploring basic questions on the biology and evolution of myxozoans. We also expect the *T. kitauei* genome to provide a useful reference for comparative genomics of eukaryotic fish parasites, particularly to identify the relative importance of shared genes among various evolutionarily distant fish parasites such as *Ichthyophthirius multifiliis*. Insights gained from this study will promote experimental approaches for myxozoan research and facilitate the development of control strategies for severe fish parasite infections and may ultimately prevent myxozoan infection in humans.

## Supplementary Material

Supplementary figures S1–S5, and tables S1–S16 are available at *Genome Biology and Evolution* online (http://gbe.oxfordjournals.org/).

Supplementary Data
