# Supplementary material for: The Genome of the Myxosporean Thelohanellus kitauei Shows Adaptations to Nutrient Acquisition within Its Fish Host
Source: Genome Biol Evol. 2014 Nov 8;6(12):3182–98. doi: 10.1093/gbe/evu247 (PMC4986447; doi:10.1093/gbe/evu247)
Supplement: Supplementary Data [file supp_6_12_3182__index.html]

The Genome of the Myxosporean Thelohanellus kitauei Shows Adaptations to Nutrient Acquisition within its Fish Host — The Genome of the Myxosporean Thelohanellus kitauei Shows Adaptations to Nutrient Acquisition within Its Fish Host — Supplementary Data 

# The Genome of the Myxosporean *Thelohanellus kitauei* Shows Adaptations to Nutrient Acquisition within Its Fish Host

## Supplementary Data

files

**Files in this Data Supplement:**

- Supplementary Data - docx file
- Supplementary Data - docx file
- Supplementary Data - xlsx file
