# Supplementary material for: The Genome of the Myxosporean Thelohanellus kitauei Shows Adaptations to Nutrient Acquisition within Its Fish Host
Source: Genome Biol Evol. 2014 Nov 8;6(12):3182–98. doi: 10.1093/gbe/evu247 (PMC4986447; doi:10.1093/gbe/evu247)

**Supplementary Material**

**Figure legends**

**Fig. S1.** Species identification of the isolated common carp parasites. **(A)**, Scanning electron micrograph of parasite spores from common carp. **(B)**, Light micrograph of parasite spores stained with 1% crystalviolet solution. Scale bar = 10 μm. **(C)**, Phylogenetic position of the 18S rDNA sequence of the common carp parasite. The neighbor-joining tree was constructed in MEGA with 1000 bootstrap reiterations (values above nodes).

**Fig. S2.** GC distribution of the assembled scaffolds.

**Fig. S3.** The 17-mer distribution frequency of the *T. kitauei* genome.

**Fig. S4.** Intron length distribution of *T. kitauei.*

**Fig. S5.** Comparison of *T. kitauei* transport and metabolic enzymes (based on KEGG pathways) with *H. magnipapillata* and *N. vectensis.*

**Supplementary Figures**

**Fig. S1.**


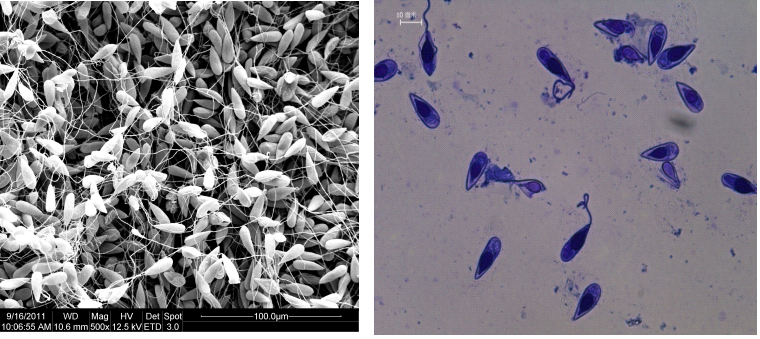

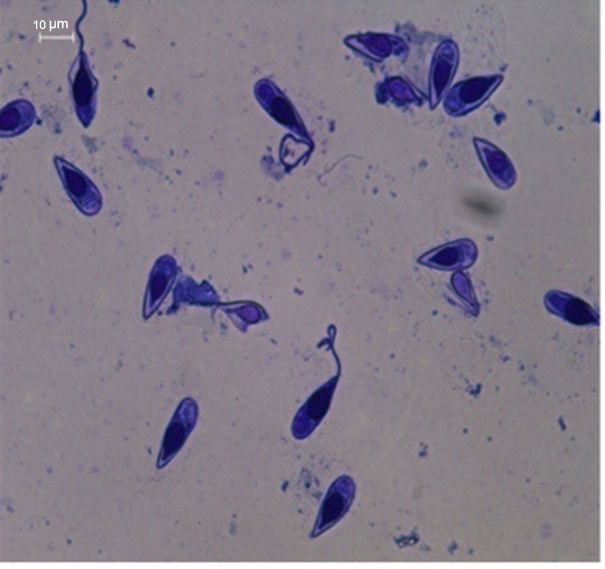


A

B

C

**Fig. S2.**


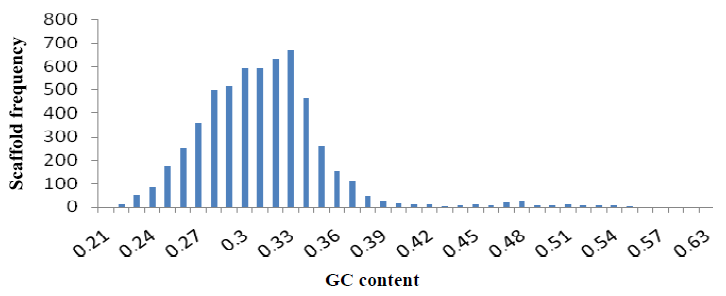


**Fig. S3.**


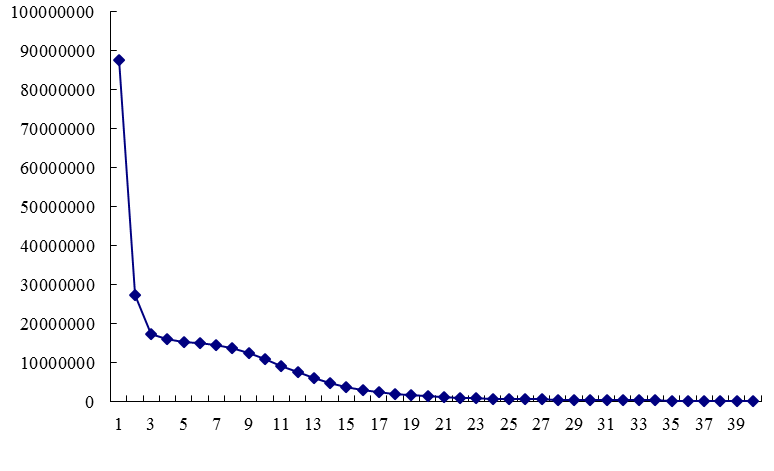


**K-mer frequency**

**Number of k-mer**

**Fig. S4.**


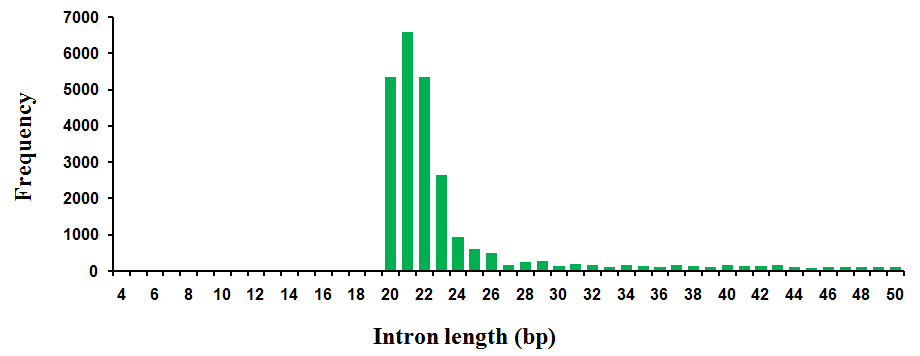


**Fig. S5.**

KEGG metabolic pathway maps were generated using the KEGG color pathways tools (http://www.genome.jp/kegg/tool/color_pathway.html). Enzyme commission numbers for all three species are indicated on the maps. Blue indicates enzymes present in all three species, green indicates enzymes present in at least one of the other free-living cnidarians but absent in *T. kitauei*, and red indicates enzymes present only in *T. kitauei*.

The notes below briefly summarize the conclusions from the each KEGG pathway analysis for the three species.

***A Transport and Catabolism***

**A1 Endocytosis excluding phagocytosis**:

All three cnidarian species are capable of endocytosis.


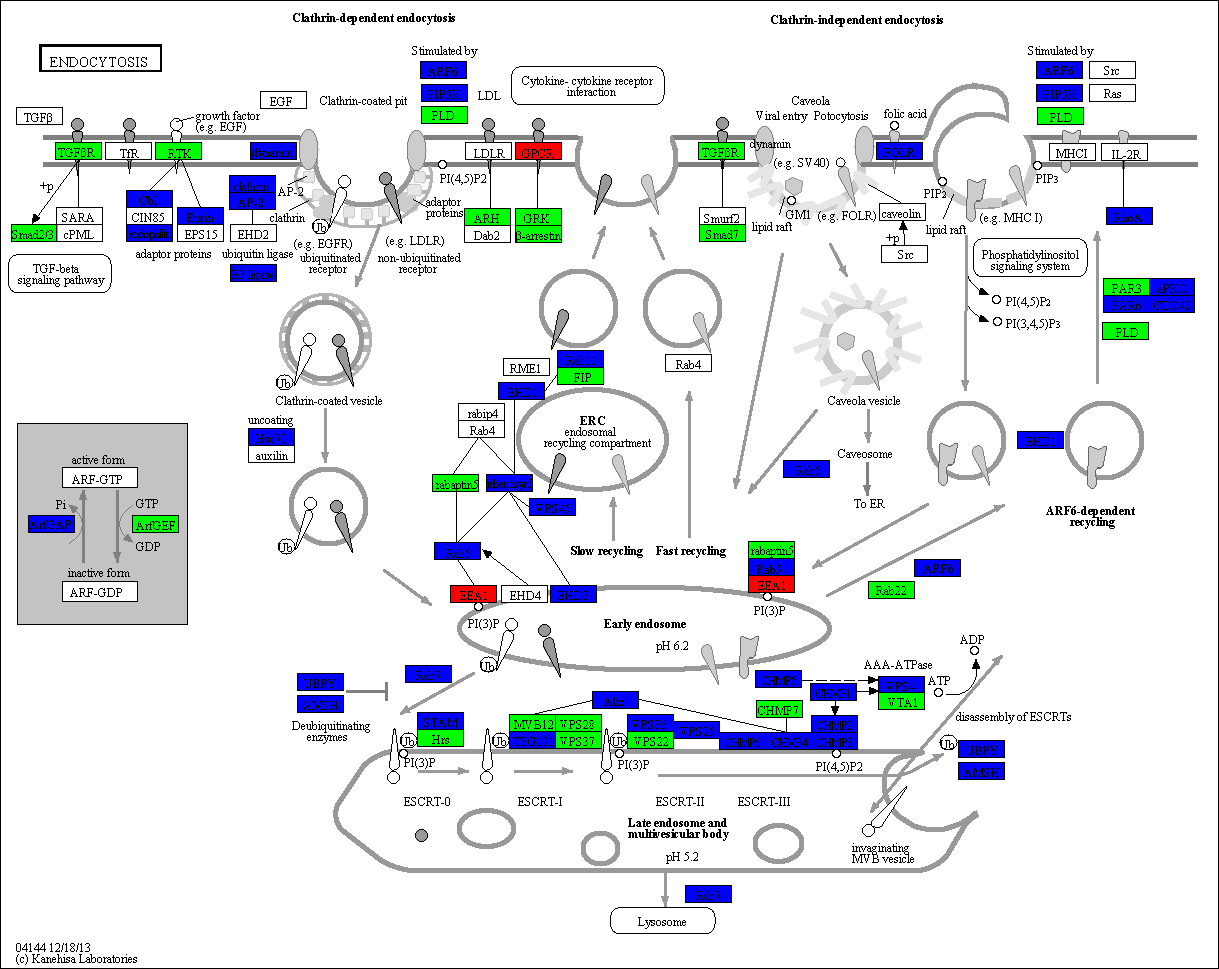


**A2 Phagosome**:

All three cnidarian species are capable of phagocytosis.


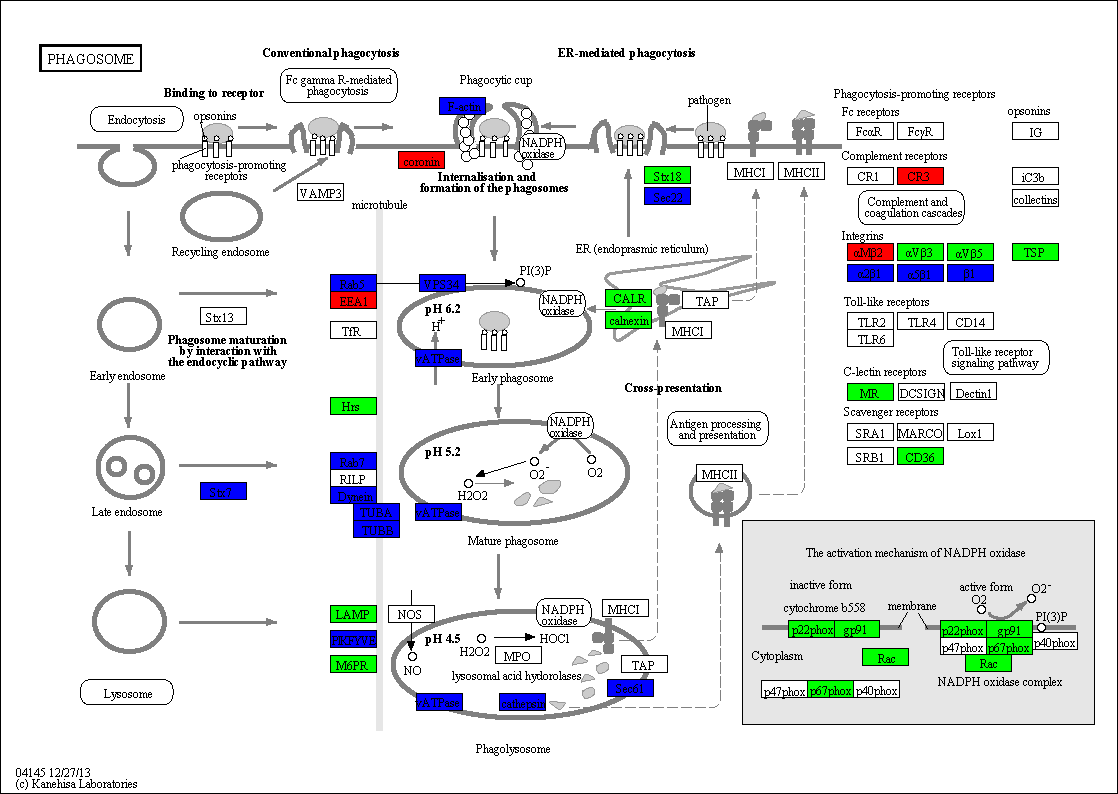


**A3 Lysosome**:

Compared with its two free-living relatives, *T. kitauei* had a reduced complexity of lysosomal membrane proteins and acid hydrolases.


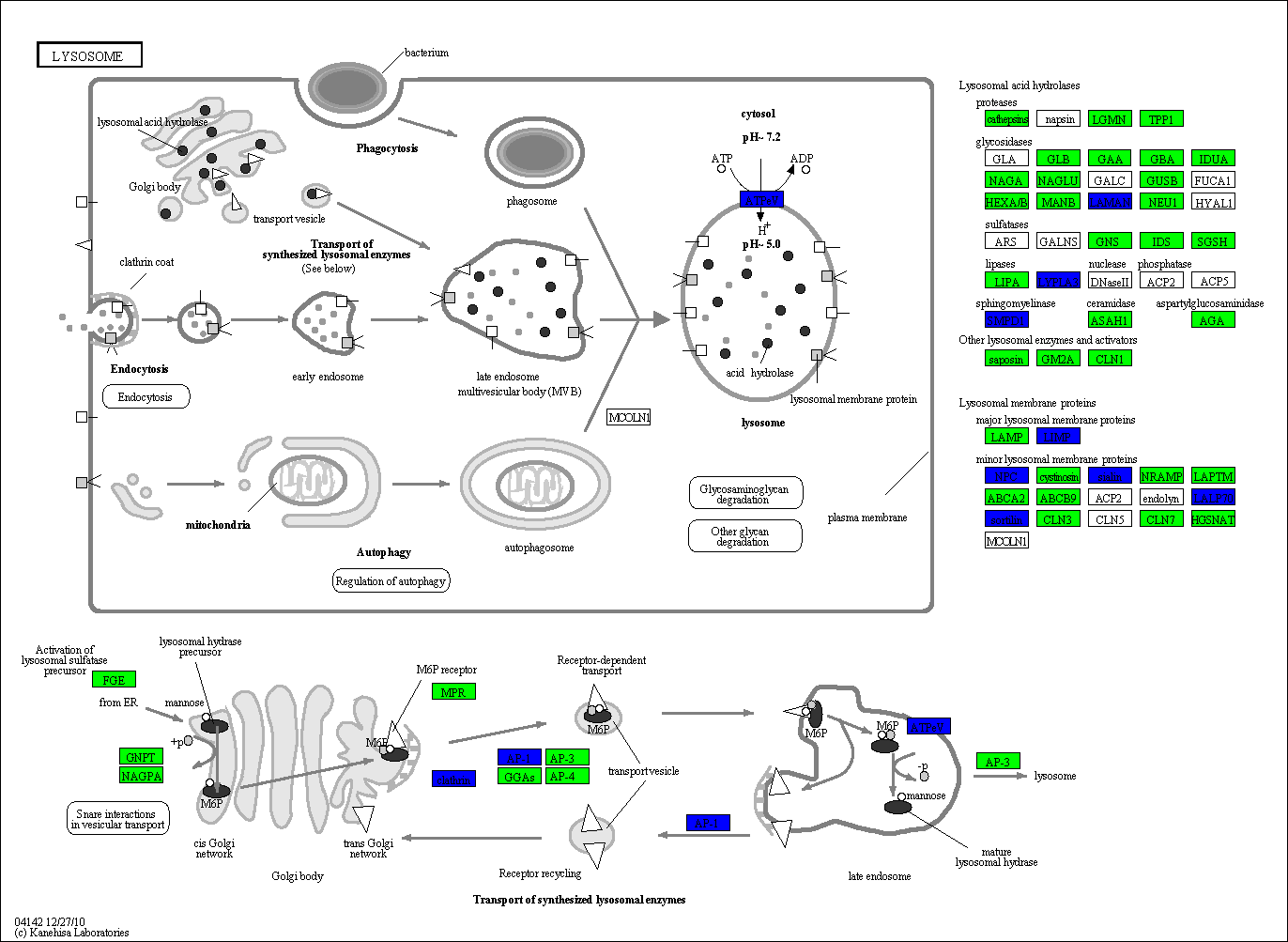


***B Carbohydrate Metabolism***

**B1 Glycolysis/Gluconeogenesis:**

Glycolysis is the same in all three species, and the two free-living cnidarians can synthesize all glycolytic metabolites by gluconeogenesis (i.e., starting with oxaloacetic acid and ending with glucose-6-phosphate). *T. kitauei* lacks fructose-bisphosphatase in the gluconeogenesis pathway, which converts fructose-1,6-bisphate to fructose-6-phosphate.


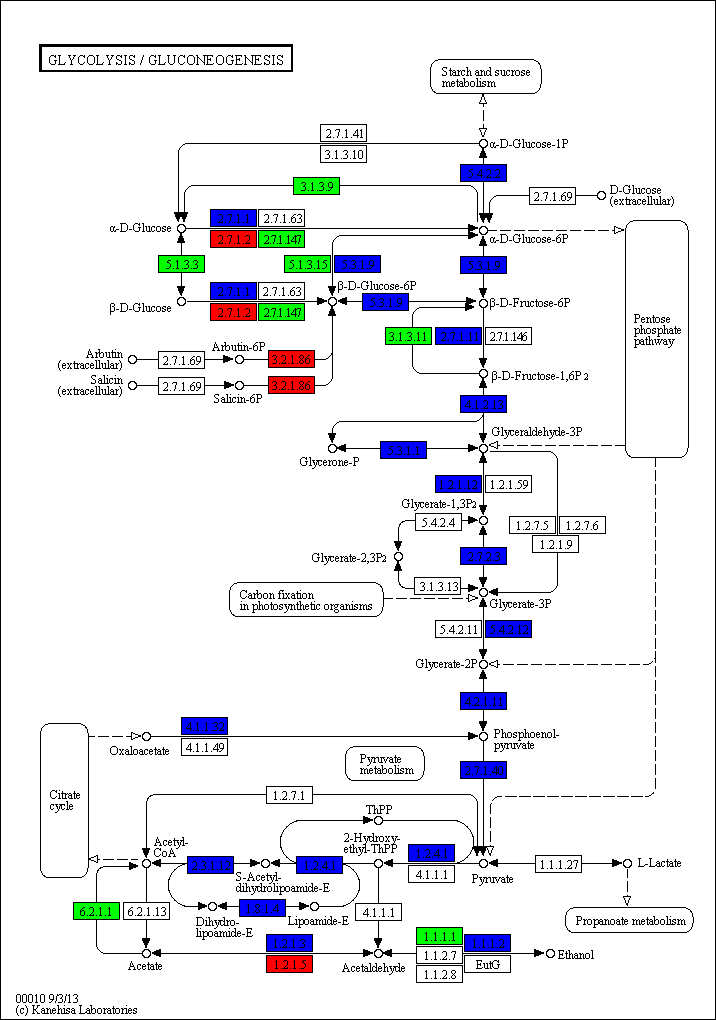


**B2 Pyruvate metabolism:**

All three species can convert pyruvate to acetyl-CoA, acetoacetyl-CoA and oxaloacetate, but none of them can convert pyruvate to L-lactate. All three species can convert acetyl-CoA to acetoacetyl-CoA and malonyl-CoA, which can be used for fatty acid biosynthesis. The two free-living species can convert acetyl-CoA to L-malate, and 3-carboxy-3-hydroxy-4-methlpentanoate, which can be used for leucine biosynthesis.


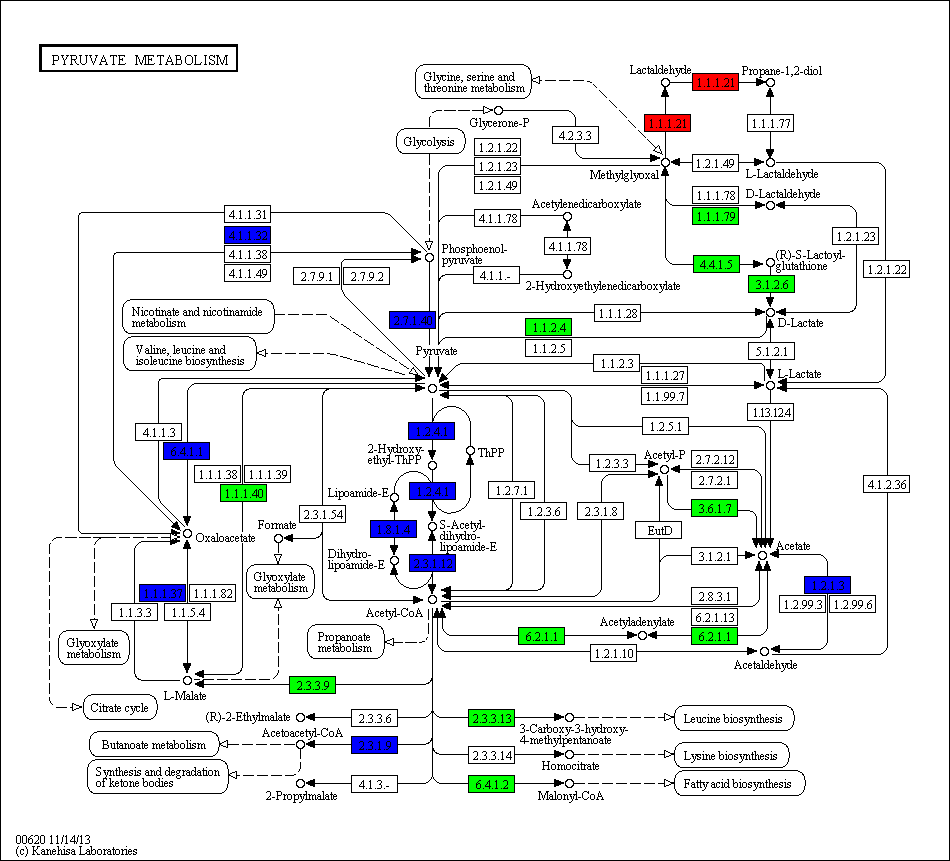


**B3 Propanoate metabolism:**

The two free-living species can metabolize propionyl-CoA to propanoate. All three species can metabolize propionyl-CoA via the methylmalonyl-CoA pathway.


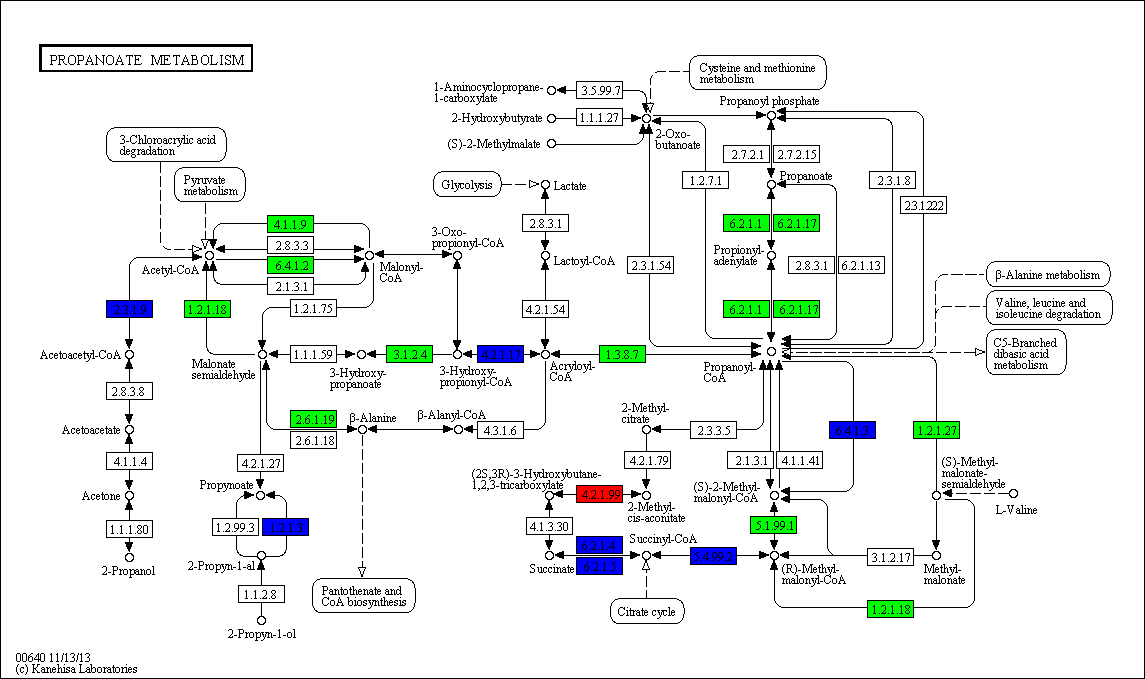


**B4 Citric acid cycle:**

All three cnidarian species have the complete citric acid cycle.


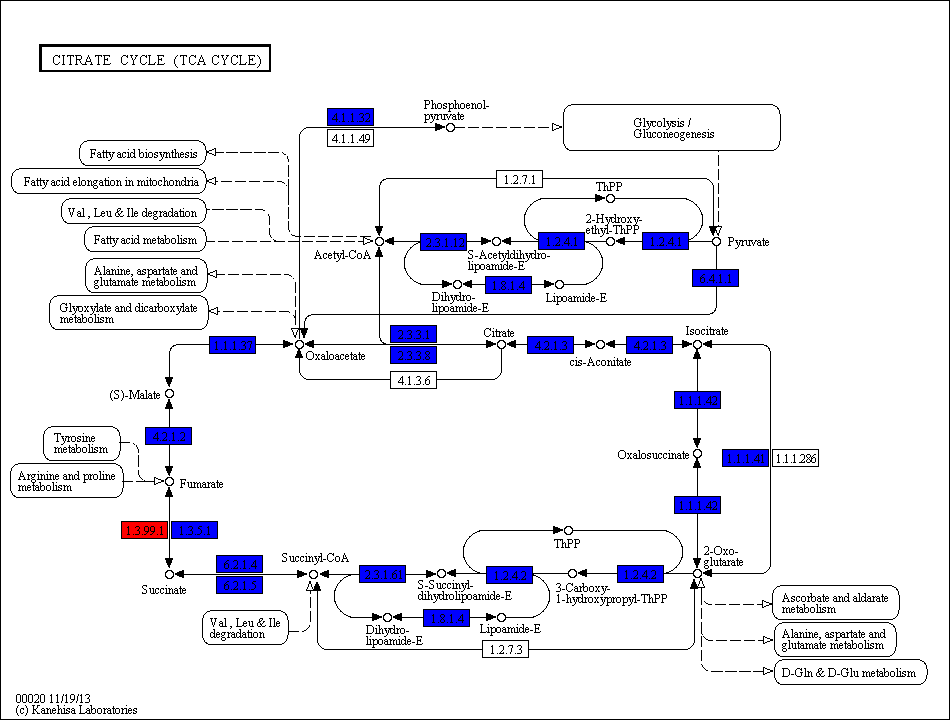


**B5 Pentose phosphate pathway:**

The two free-living cnidarians have the complete pentose phosphate pathway. *T. kitauei* has the transaldolase-deﬁcient pentose phosphate pathway.


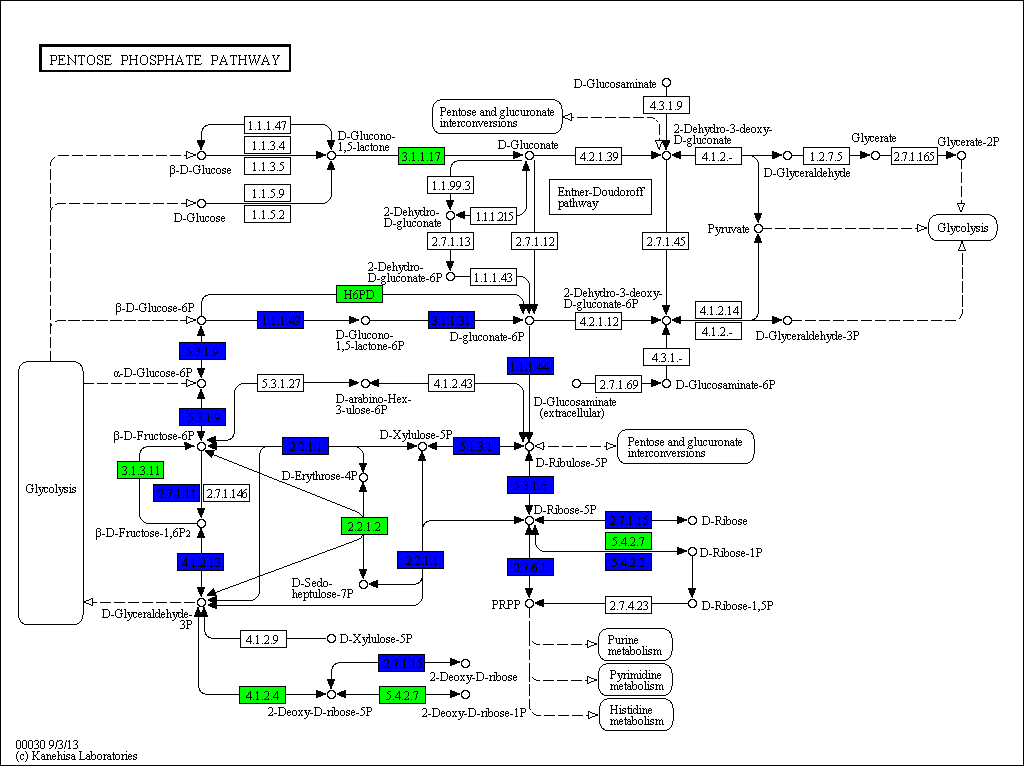


**B6 Amino sugar and nucleotide sugar metabolism:**

All three species can synthesize UDP-glucose, uridine diphosphate N-acetylglucosamine, [GDP-man](http://en.wikipedia.org/wiki/GDP-mannose)nose, and CMP-N-acetylneuraminic acid. All three species can convert UDP-glucose to UDP-galactose or UDP-xylose and GDP-mannose to GDP-fucose. The two free-living cnidarians can also synthesize UDP-galactose and GDP-fucose from the respective monosaccharides*.* The two free-living cnidarians can form and degrade chitin.


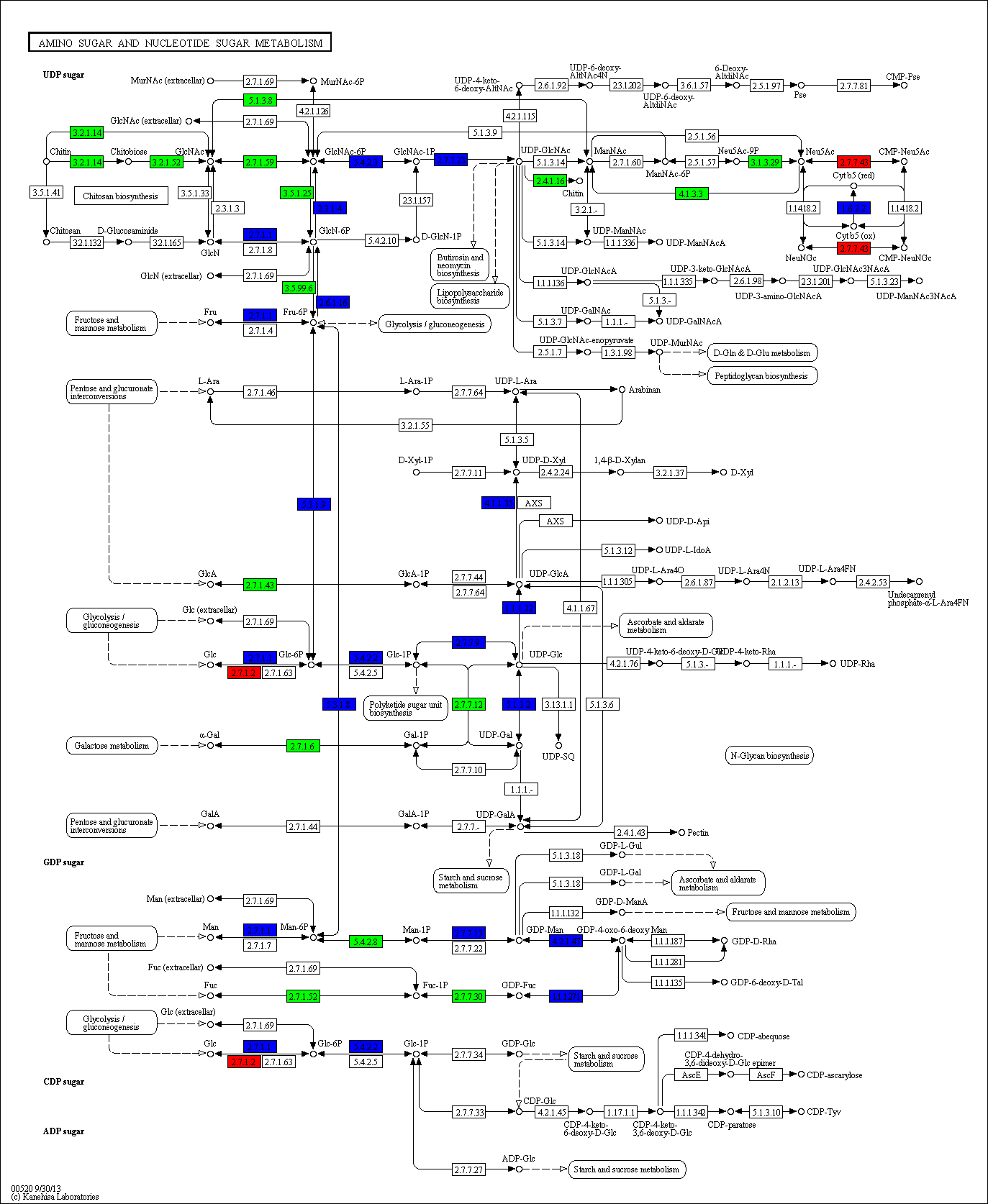


**B7 Pentose and** **glucuronate interconversion:**

Pentose and glucuronate interconversion is absent in all three cnidarians.


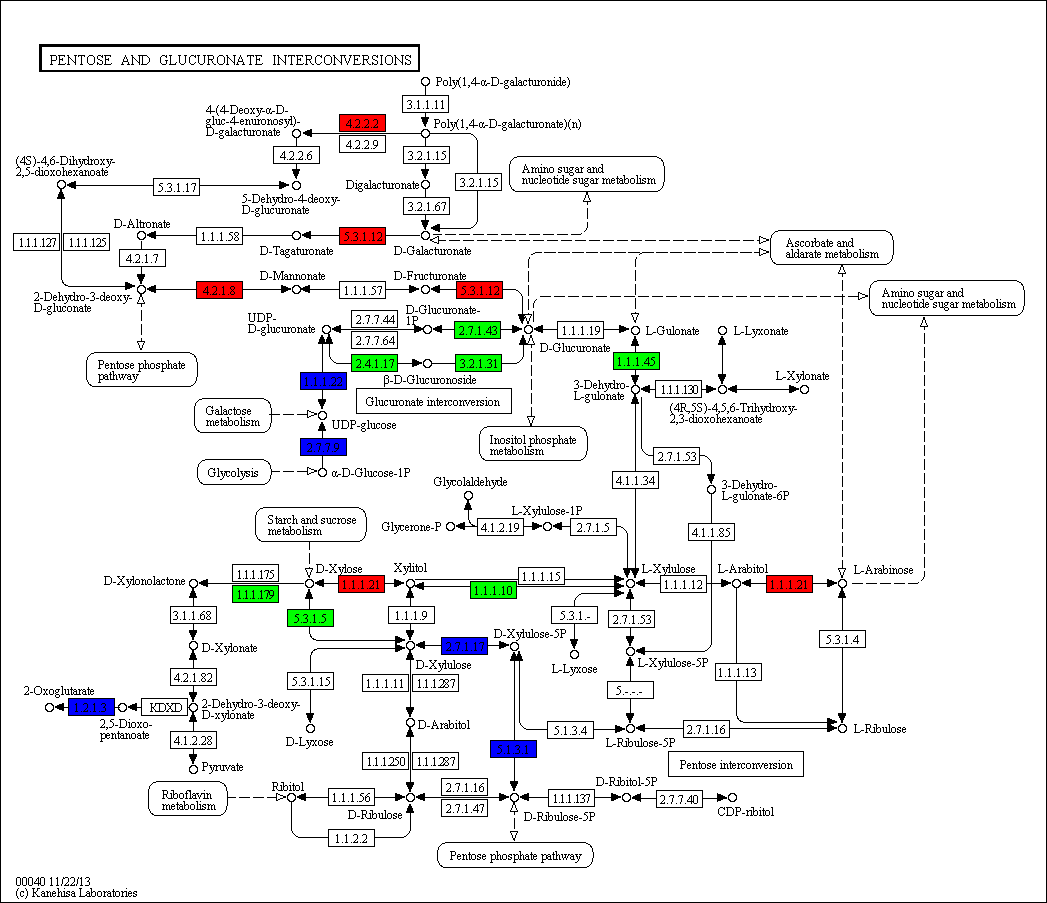


**B8 Fructose and mannose metabolism:**

There are no major differences between the three species.


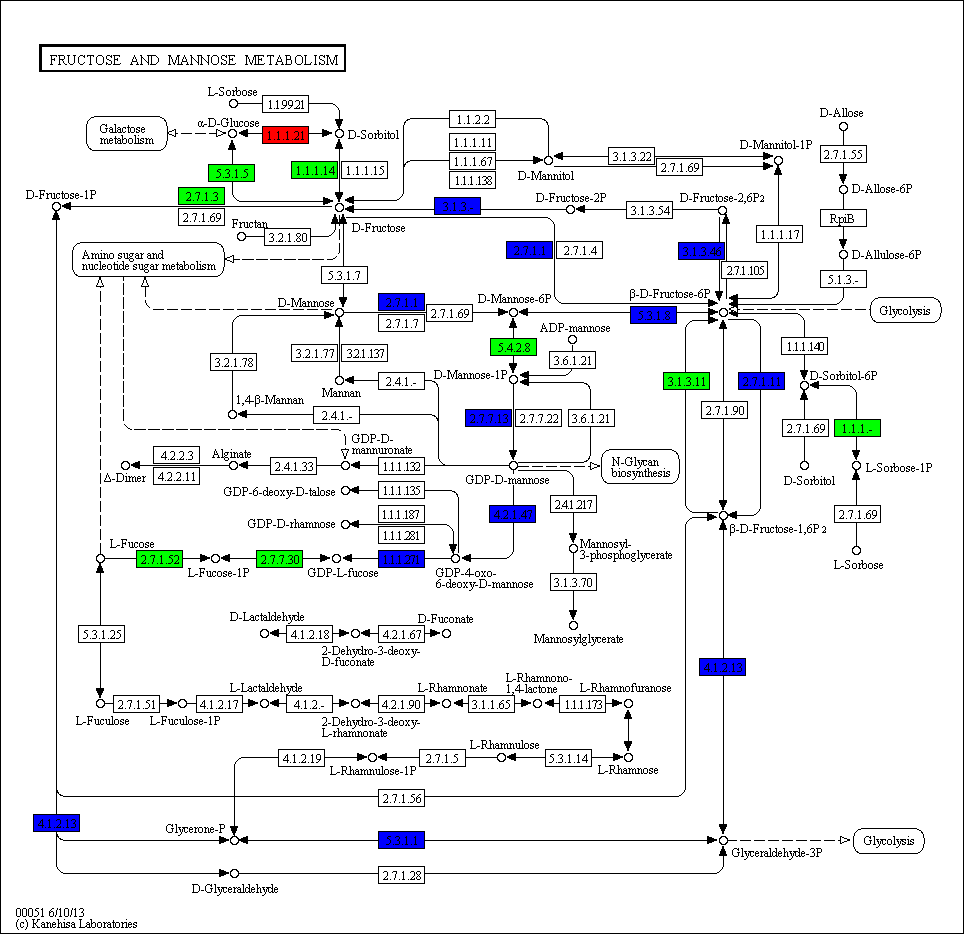


**B9 Galactose metabolism:**

*H. magnipapillata* and *N. vectensis* can convert galactose into glucose. *T. kitauei* can convert glucose to UDP-galactose to form lactose. In *T. kitauei*, galactose appears to be produced by hydrolysis of manninotriose, melibiose, stachyose, melibitol, galactinol, and epimelibiose, hydrolysis of galactosylglycerol by galactosidase to yield α-galactose, or by hydrolysis of galactitol by aldehyde reductase.


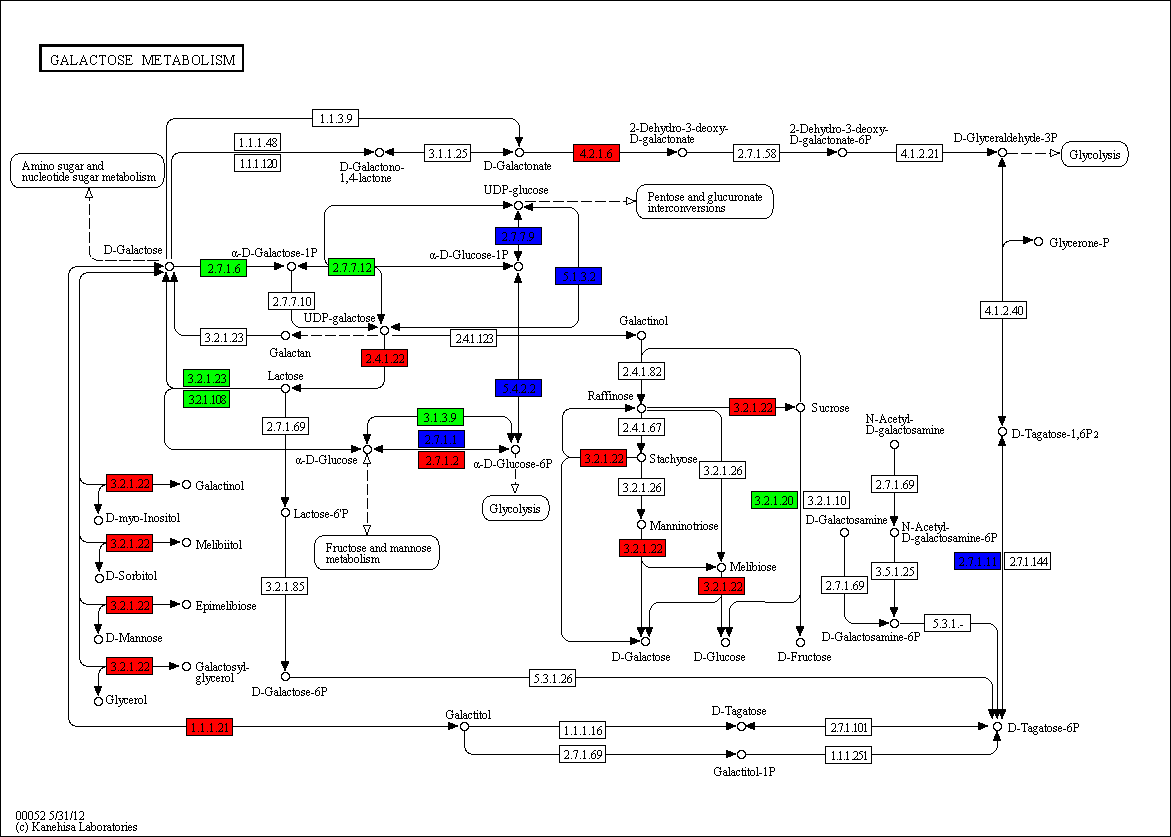


**B10 Ascorbate and** **aldarate metabolism:**

All three cnidarians lack the factors necessary for metabolism of ascorbate and aldarate. All three cnidarians can convert L-gulonate or L-gulono-1,4-lactone to L-ascorbate or convert D-glucurono-lactone to D-glucarate, and *T. kitauei* can still convert L-arabinono-1,4-lactone to L-arabinonate.


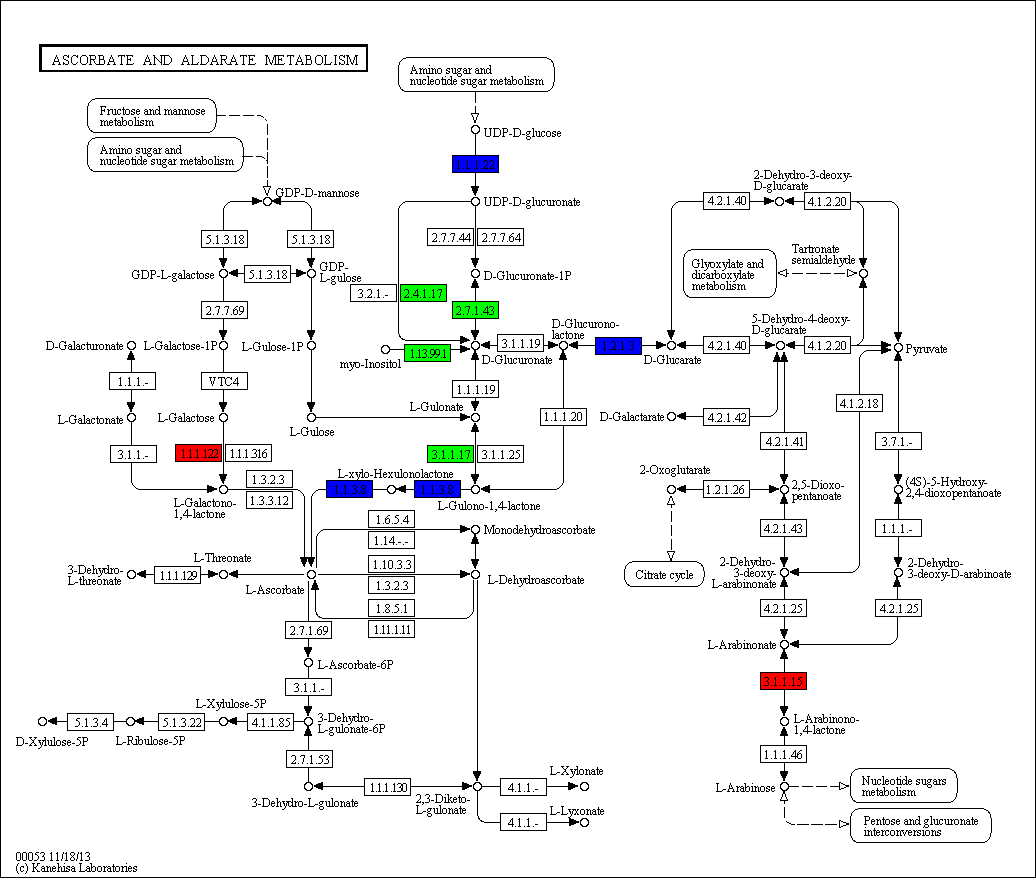


**B11 Starch and sucrose metabolism:**

All three cnidarians are capable of synthesizing and degrading starch and glycogen utilizing maltose, but only the two free-living cnidarians can degrade sucrose.


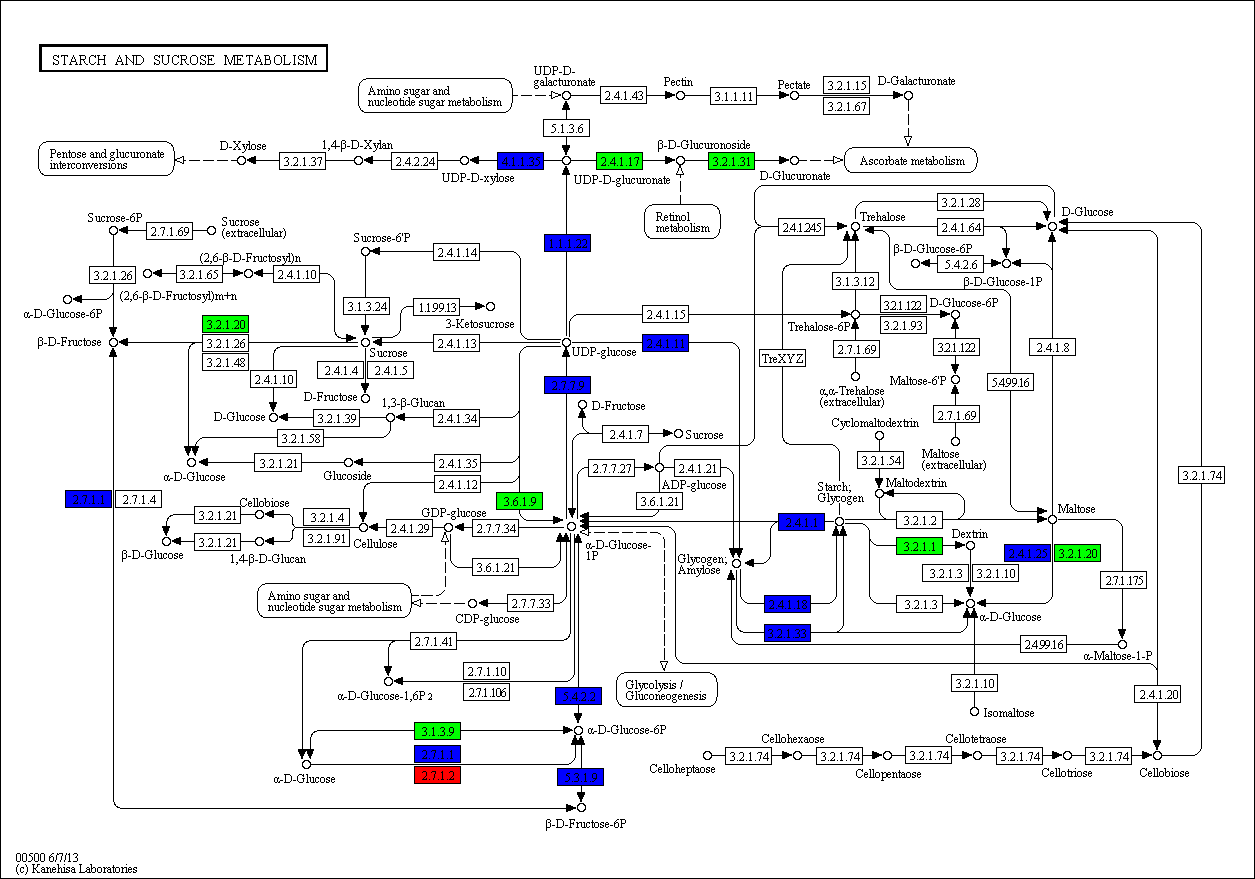


**B12 Inositol phosphate** **metabolism:**

In all three species, inositol phosphate can be formed either as a product of phosphatidylinositol phosphate metabolism or from glucose-6-phosphate via the enzyme inositol-3-phosphate synthase 1.


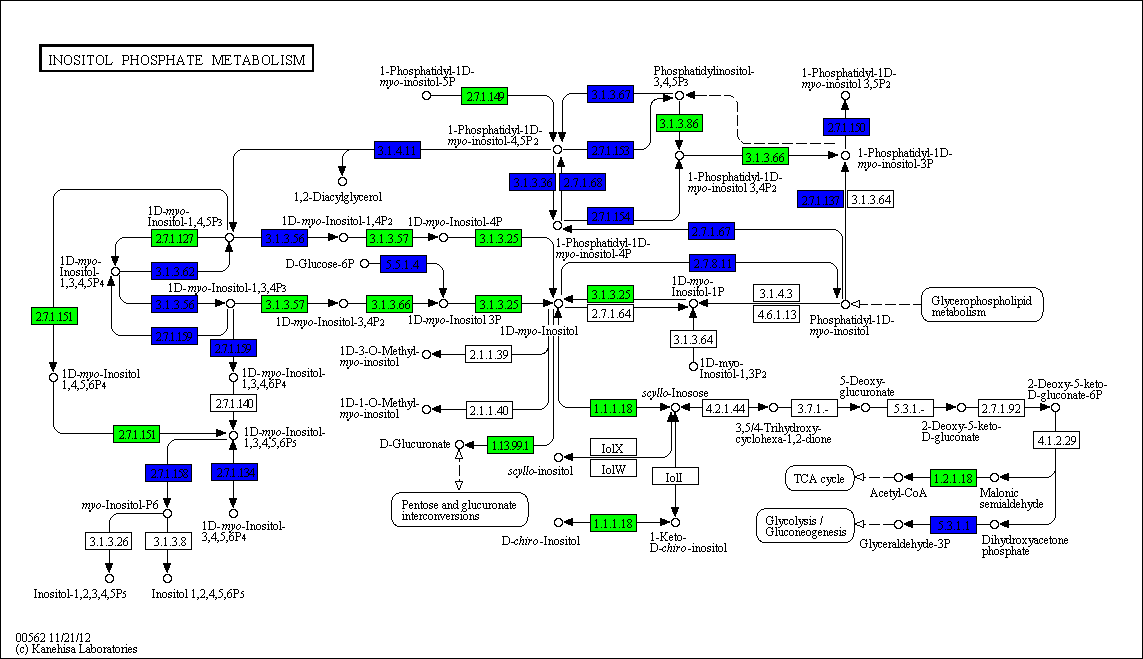


**B13 Glyoxylate and dicarboxylate metabolism:**

All three species lack the key enzyme of the glyoxylate cycle, isocitrate lyase, which allows conversion of isocitrate to succinate and glyoxylate. *T. kitauei* and *H. magnipapillata* also lack malate synthase, which converts glyoxylate directly to malate. The two free-living species have a more comprehensive set of enzymes and can convert glycolate to glyoxylate and hydroxy-pyruvate to tartronate-semialdehyde. *N. vectensis* can convert glycolate to hydroxypyruvate.


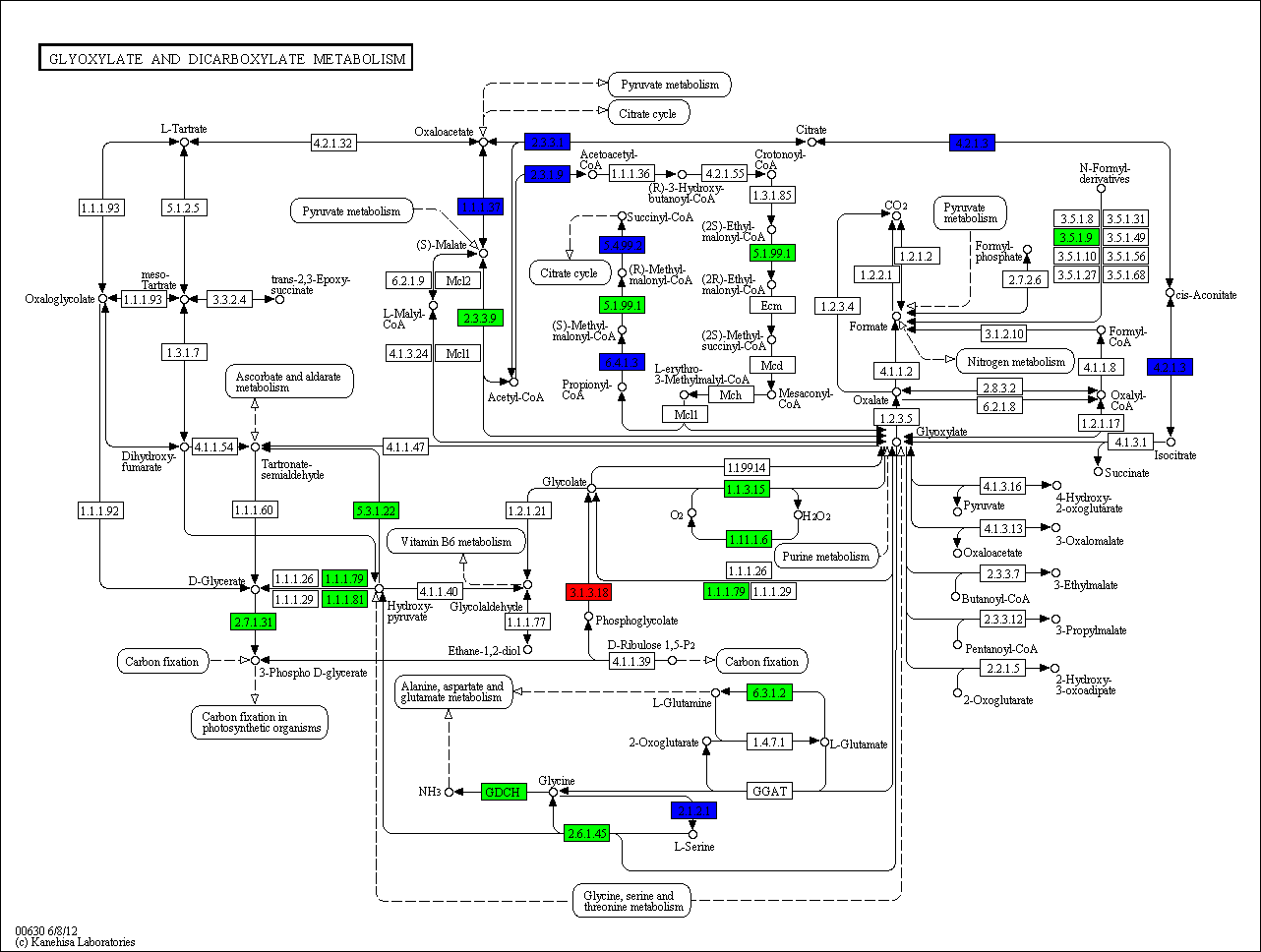


***C Energy Metabolism***

**C1 Oxidative phosphorylation and F_1_F_0_-ATP synthase:**

This pathway is present in all three species, but the two free-living species have a more comprehensive set of enzymes. NADH-ubiquinone oxidoreductase and NDUFB1 NADH dehydrogenase (ubiquinone) 1 β subcomplexes are present in the two free-living species and absent in *T. kitauei*.


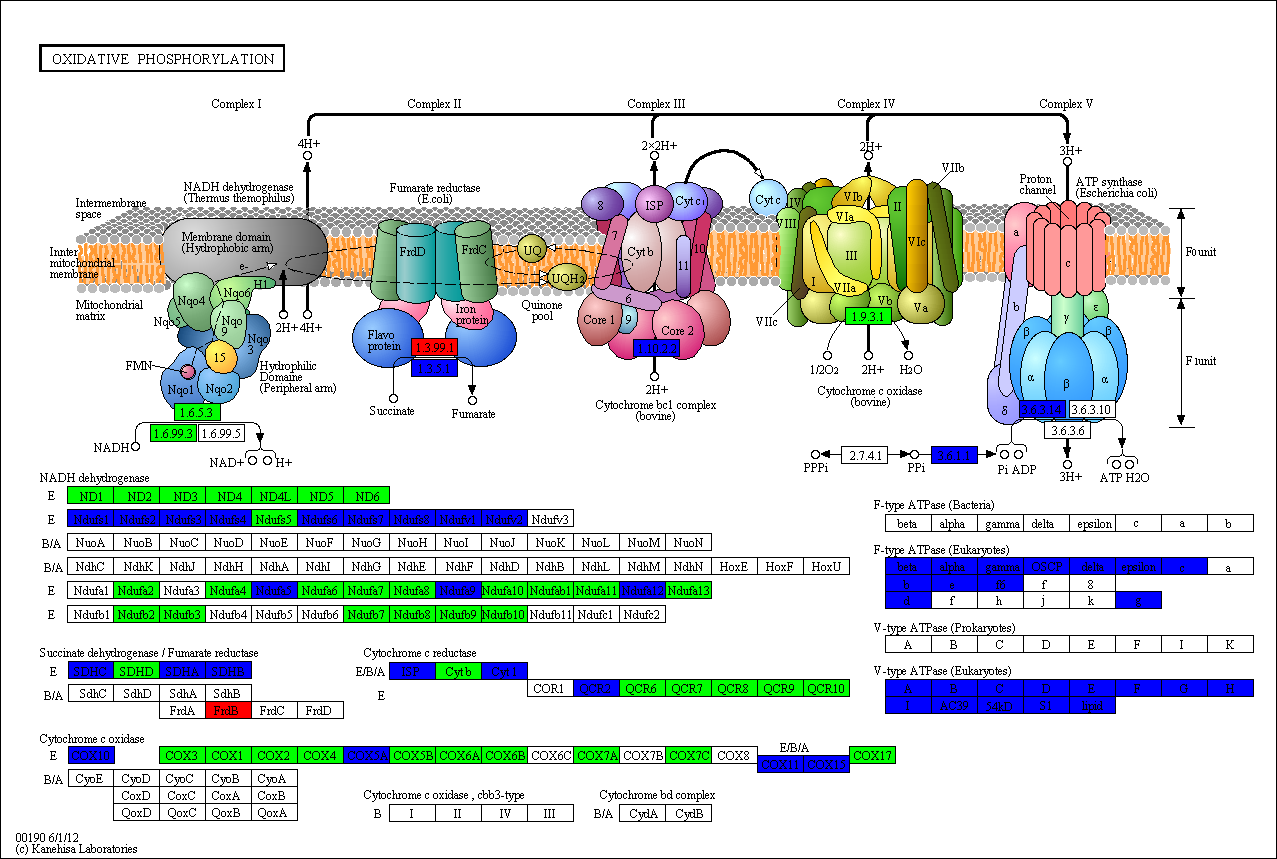


**C2 Nitrogen metabolism:**

There are two major routes for ammonium assimilation in the two free-living species, namely the glutamine synthetase and glutamate synthase cyclic mechanism and the glutamate dehydrogenase pathway. There is only the glutamate dehydrogenase pathway for ammonium assimilation in *T. kitauei*. Nitrate reductases, which can catalyze NAD(P)H reduction of nitrate to nitrite, are present in *T. kitauei* but absent in the two free-living species.


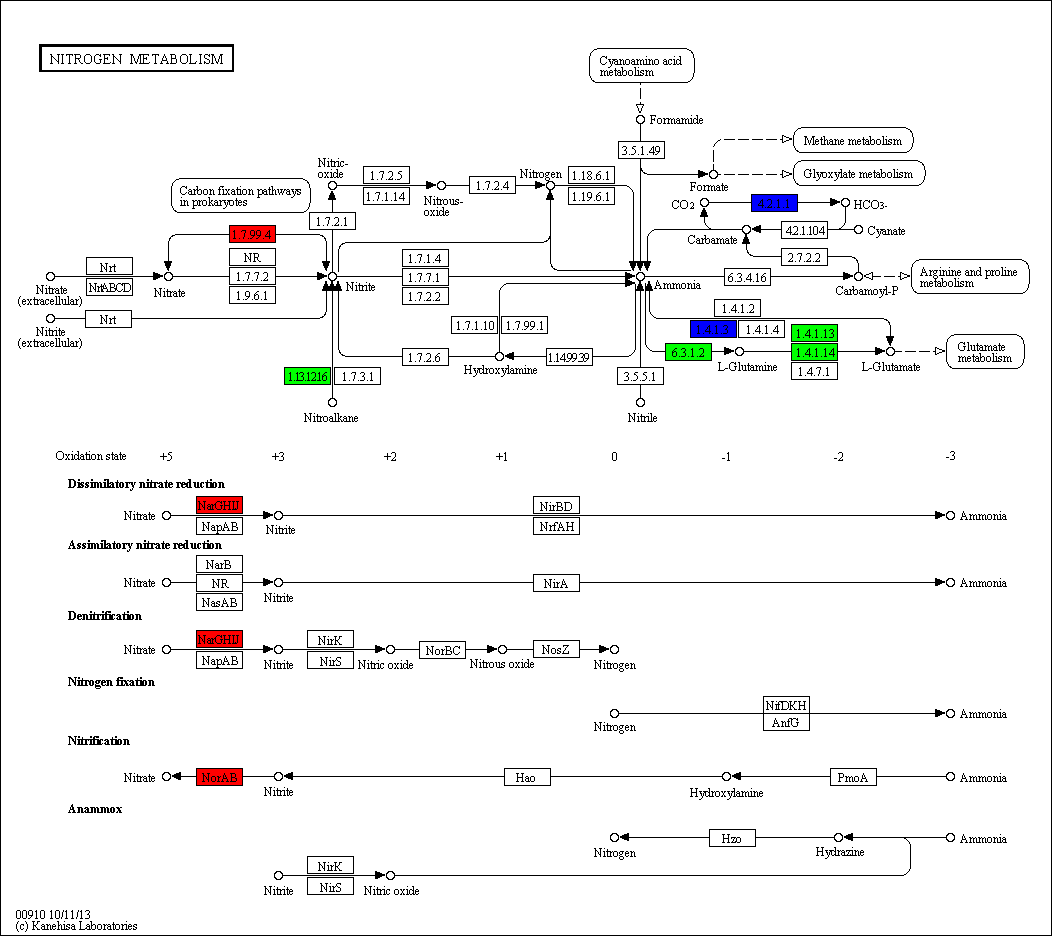


**C3 Sulfur metabolism:**

All three species are capable of synthesizing phosphoadenylyl sulfate and have the cysteine synthase enzyme, which can use H_2_S as a sulfur donor for making cysteine.


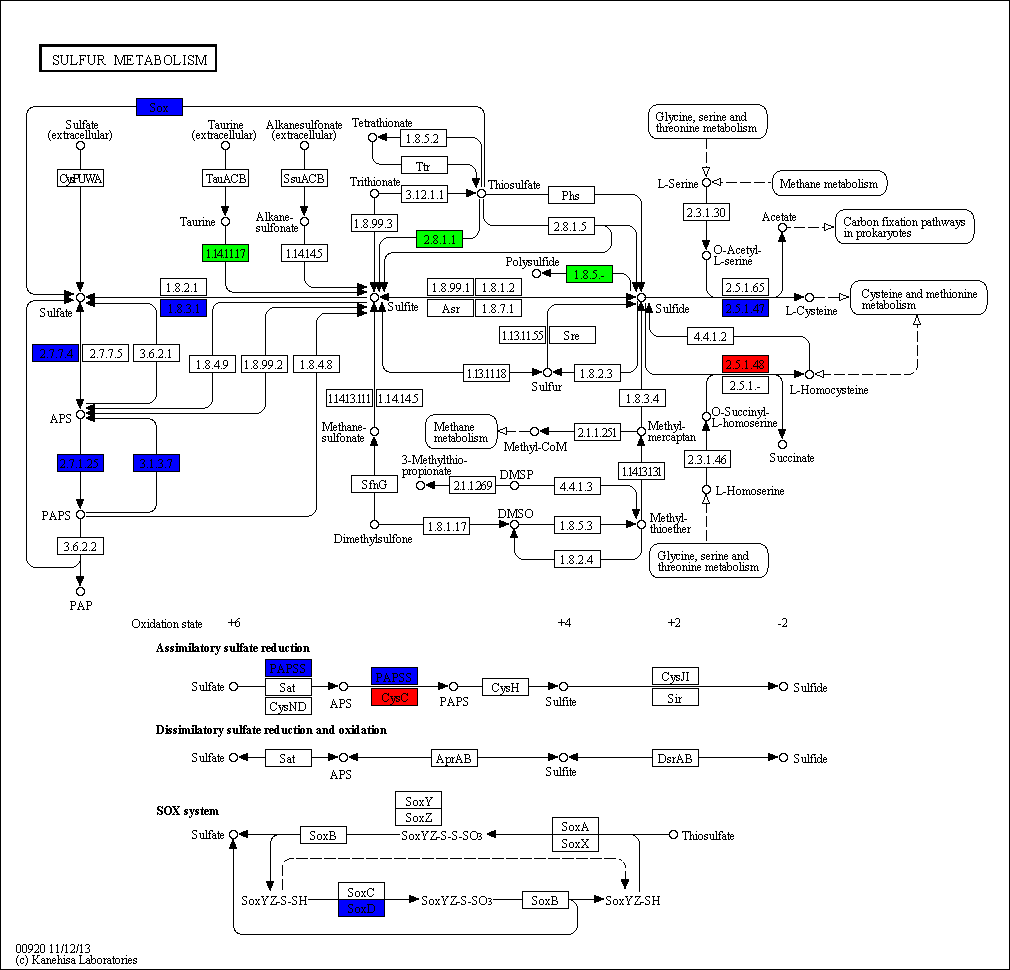


***D Amino Acid Metabolism***

**D1 Alanine, aspartate and glutamate metabolism:**

The two free-living species have a more comprehensive set of enzymes than *T. kitauei*.  In *T. kitauei*, alanine, aspartate and glutamate are formed by transamination of pyruvate, oxaloacetate and 2-ketoglutarate, respectively.

In amino acid catabolism of *T. kitauei*, alanine, aspartate and glutamate are transaminated, respectively, to pyruvate, oxaloacetate and 2-ketoglutarate, and then oxidized via the citric acid cycle. Aspartate may also be converted to fumarate.


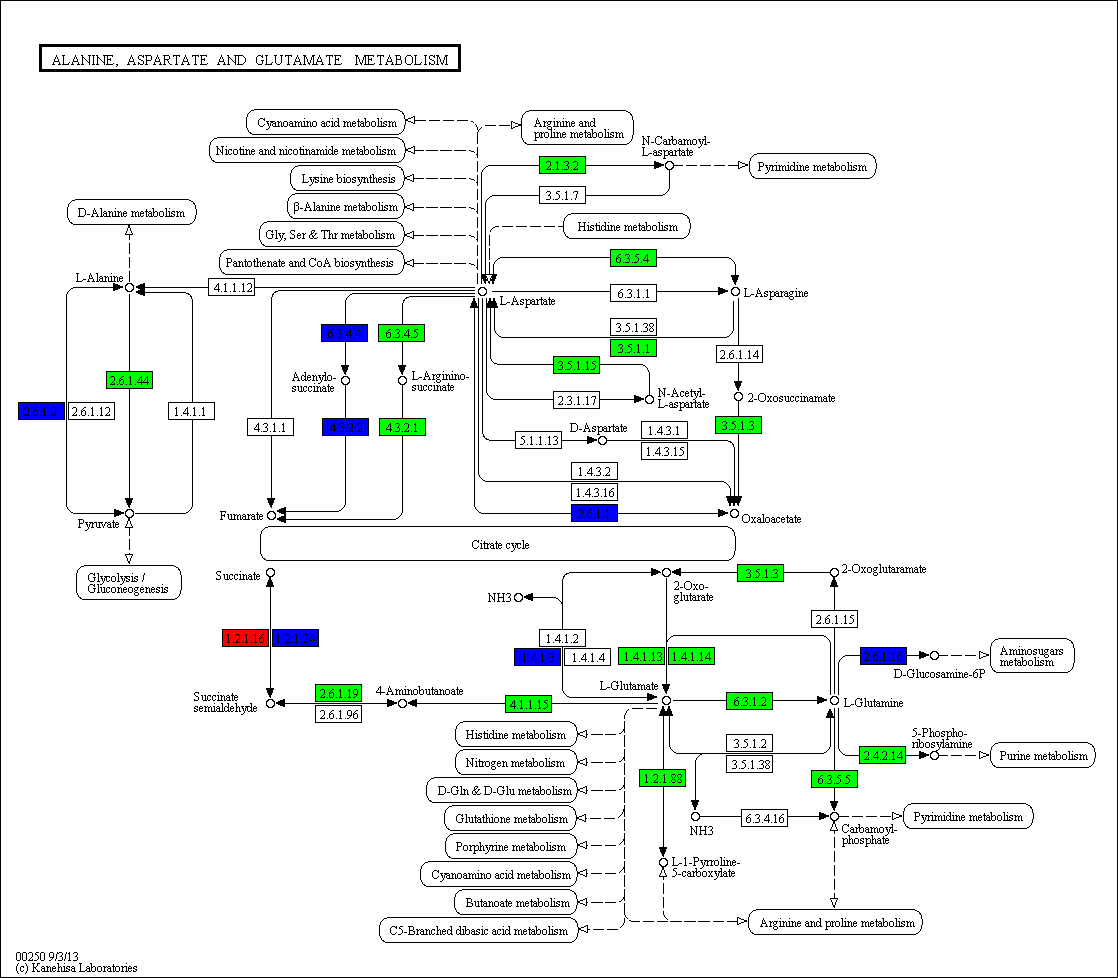


**D2 Glycine, serine and** **threonine metabolism:**

The two free-living species have a more comprehensive set of enzymes than *T. kitauei*. *T. kitauei* cannot synthesize glycine and serine *de novo* but they can be interconverted by hydroxymethyl transferase; the latter can also be used for the synthesis of cysteine. *T. kitauei* lacks threonine aldolase, which catalyze the conversion between glycine and threonine.

**
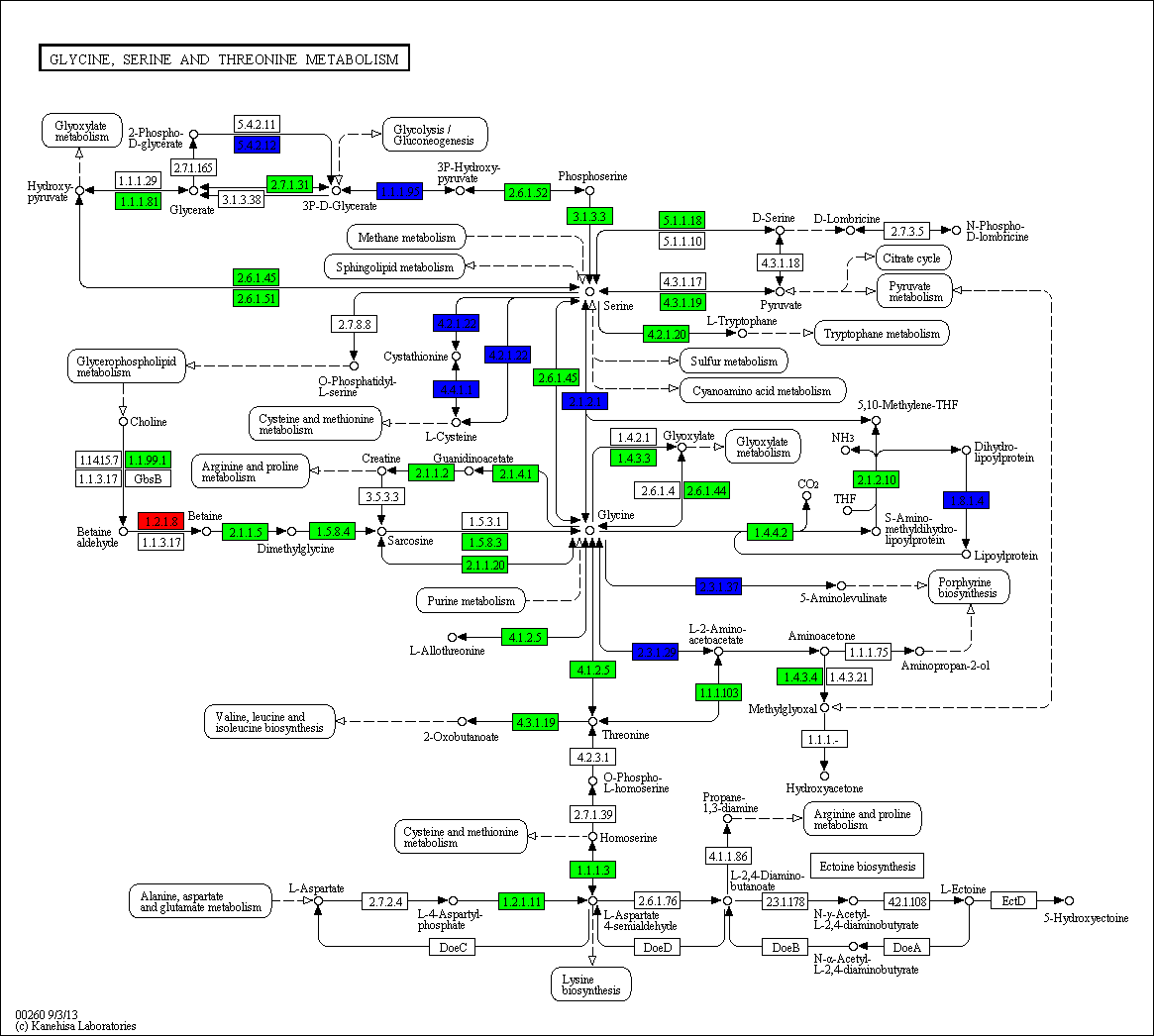
**

**D3 Cysteine and methionine metabolism:**

There are no major difference between the three species. *T. kitauei* encodes the enzyme cysteine synthase, which can use H_2_S as a sulfur donor to synthesize cysteine, and methionine can be salvaged from homocysteine. Only *T. kitauei* has the transsulfuration enzyme cystathionine γ-synthase, which catalyzes the first specific step in L-methionine biosynthesis. *T. kitauei* can use methionine to produce S-methyl-5′-thioadenosine. *T. kitauei* lacks methionine salvage and converts cysteine to pyruvate for further metabolism to acetyl-CoA.


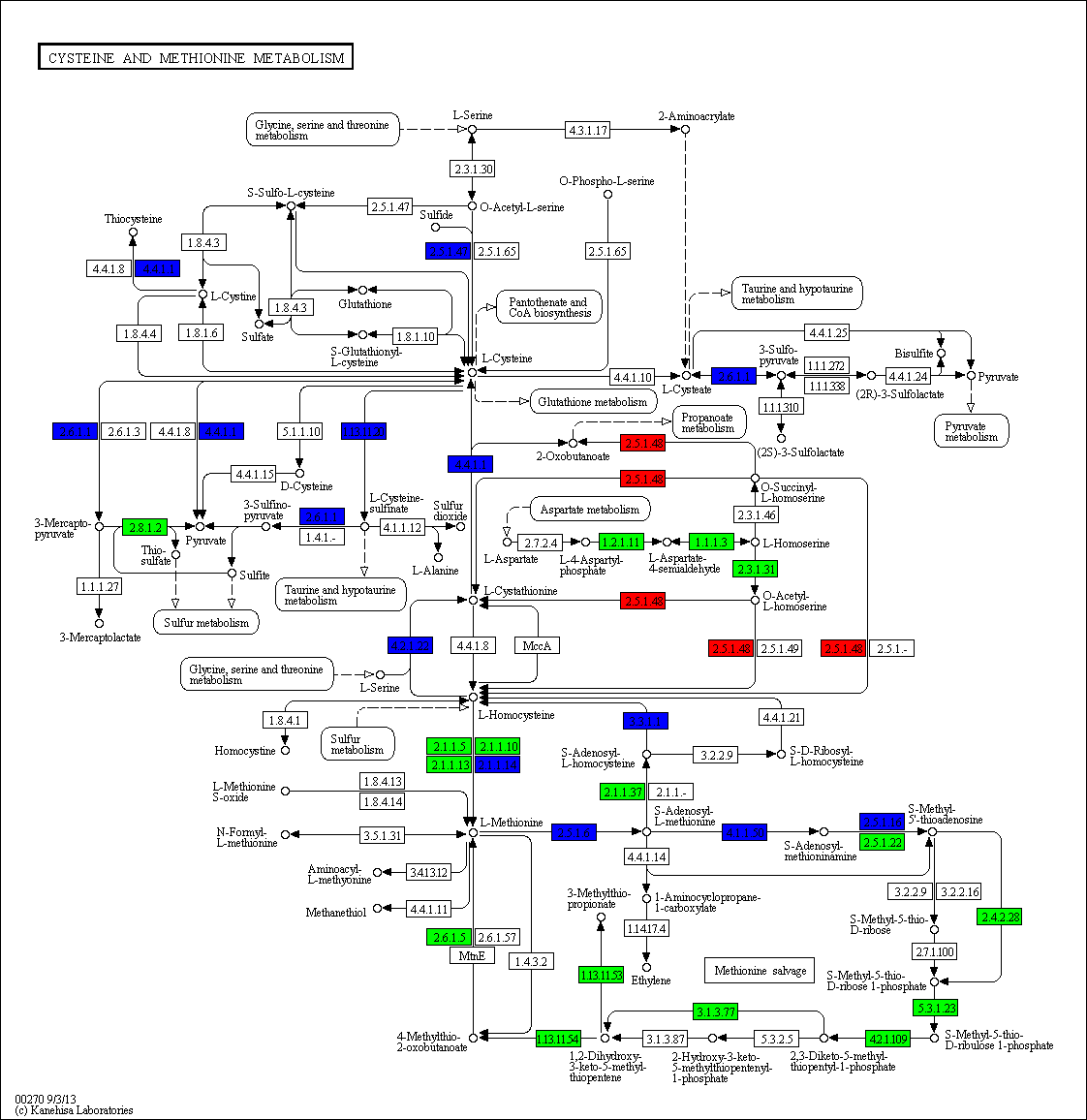


**D4 Valine, leucine and isoleucine degradation:**

Unlike *T. kitauei*, *H. magnipapillata* and *N. vectensis* are capable of degrading these three amino acids.


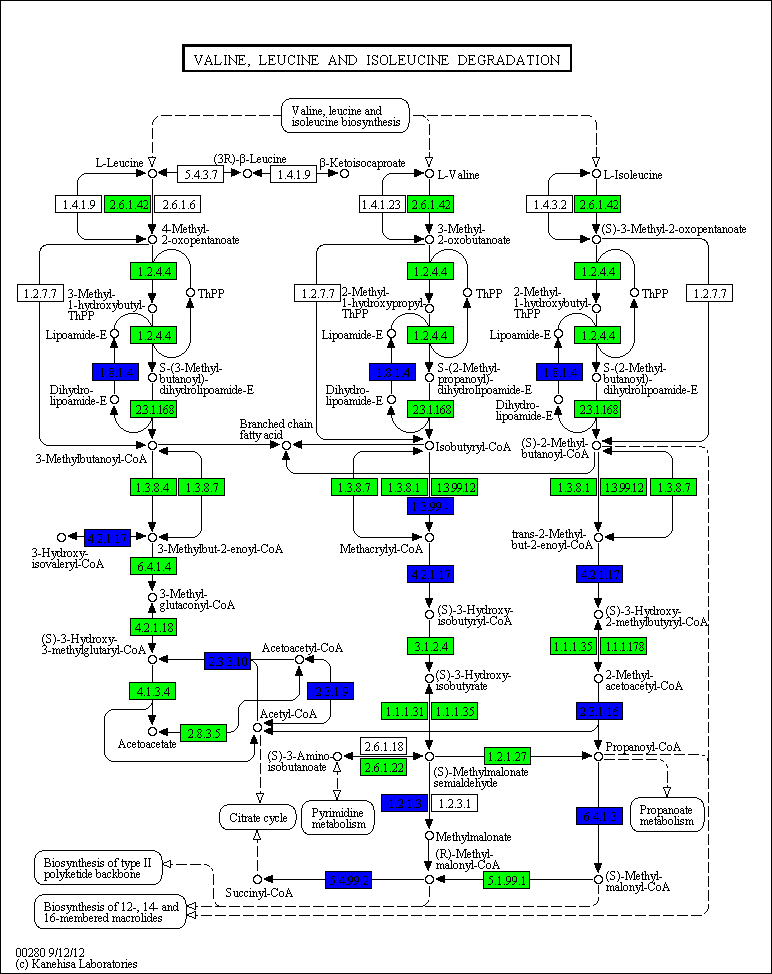


**D5 Valine, leucine and isoleucine biosynthesis:**

Unlike *T. kitauei*, *H. magnipapillata* and *N. vectensis* are partially capable of synthesizing these three amino acids.


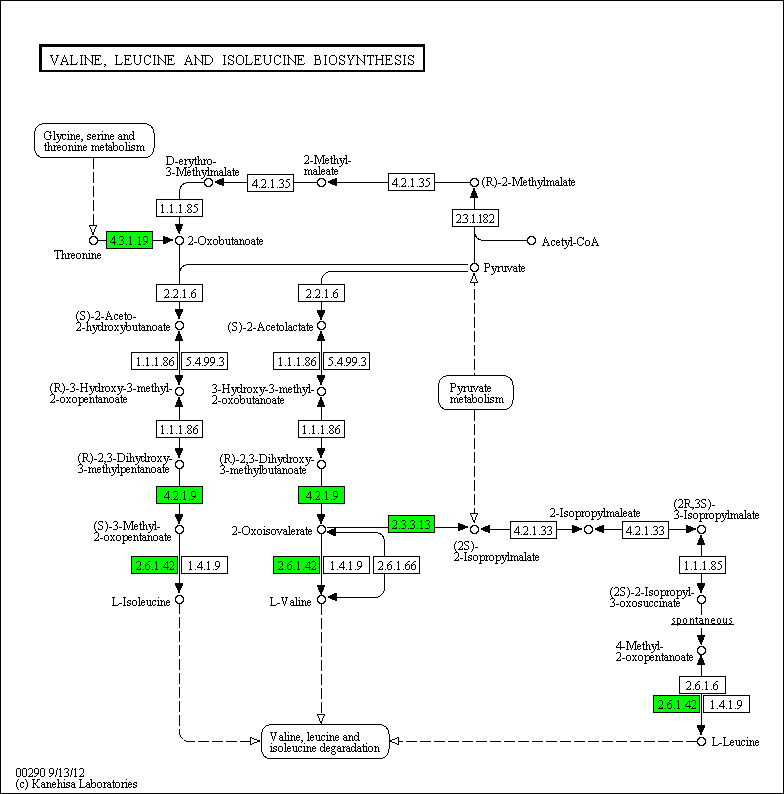


**D6 Lysine biosynthesis:**

*T. kitauei* and *H. magnipapillata* cannot synthesize lysine. Only *N. vectensis* has saccharopine dehydrogenase, which is the last enzyme in l-lysine biosynthesis.


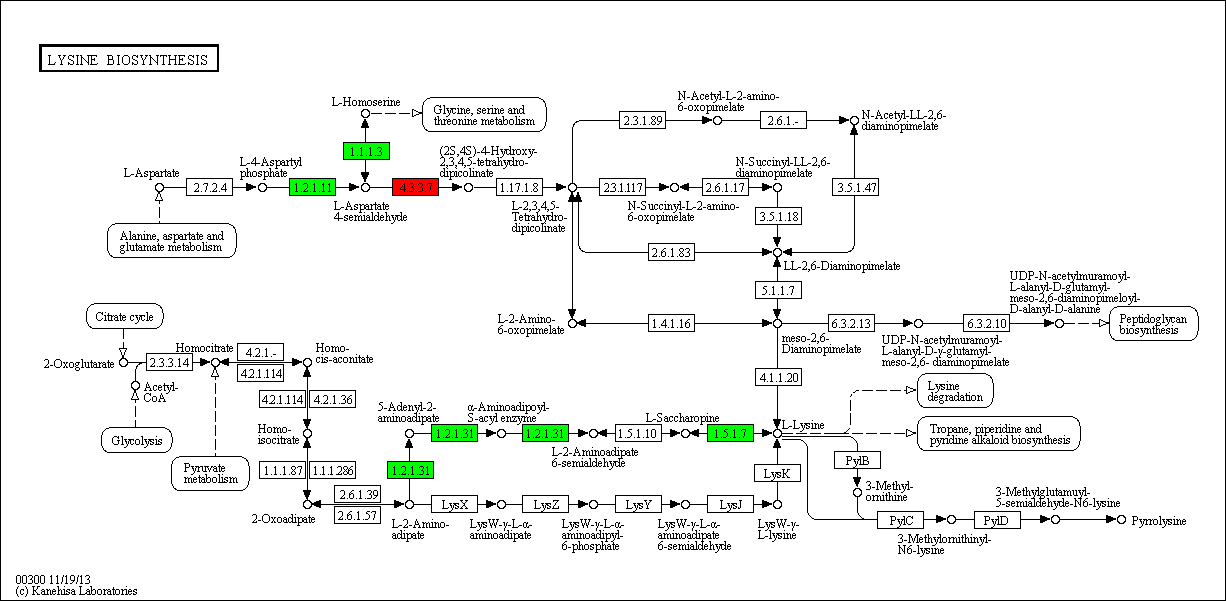


**D7 Lysine degradation:**

*H. magnipapillata and N. vectensis* have similar sets of enzymes for lysine degradation, which appear to be lacking in *T. kitauei*.

*
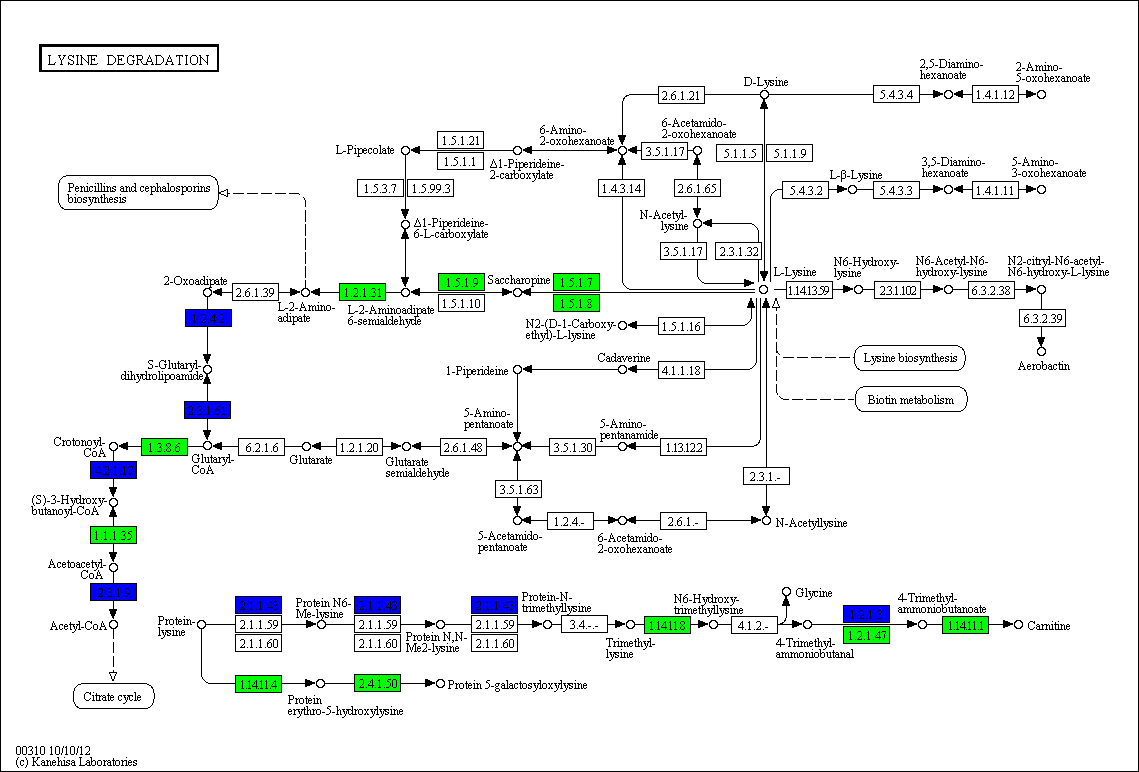
*

**D8 Arginine and proline metabolism:**

*H. magnipapillata* and *N. vectensis* carry out the urea cycle, but this pathway is missing in *T. kitauei*. Pathways linking glutamine and proline or ornithine and arginine also are absent in *T. kitauei*. In the three species, ornithine and proline can be interconverted by glutamate-5-semialdehyde, and the latter can also be formed by degradation of peptides. The three species have the polyamine pathway and can convert ornithine to putrescine, then to spermidine, and finally spermine.


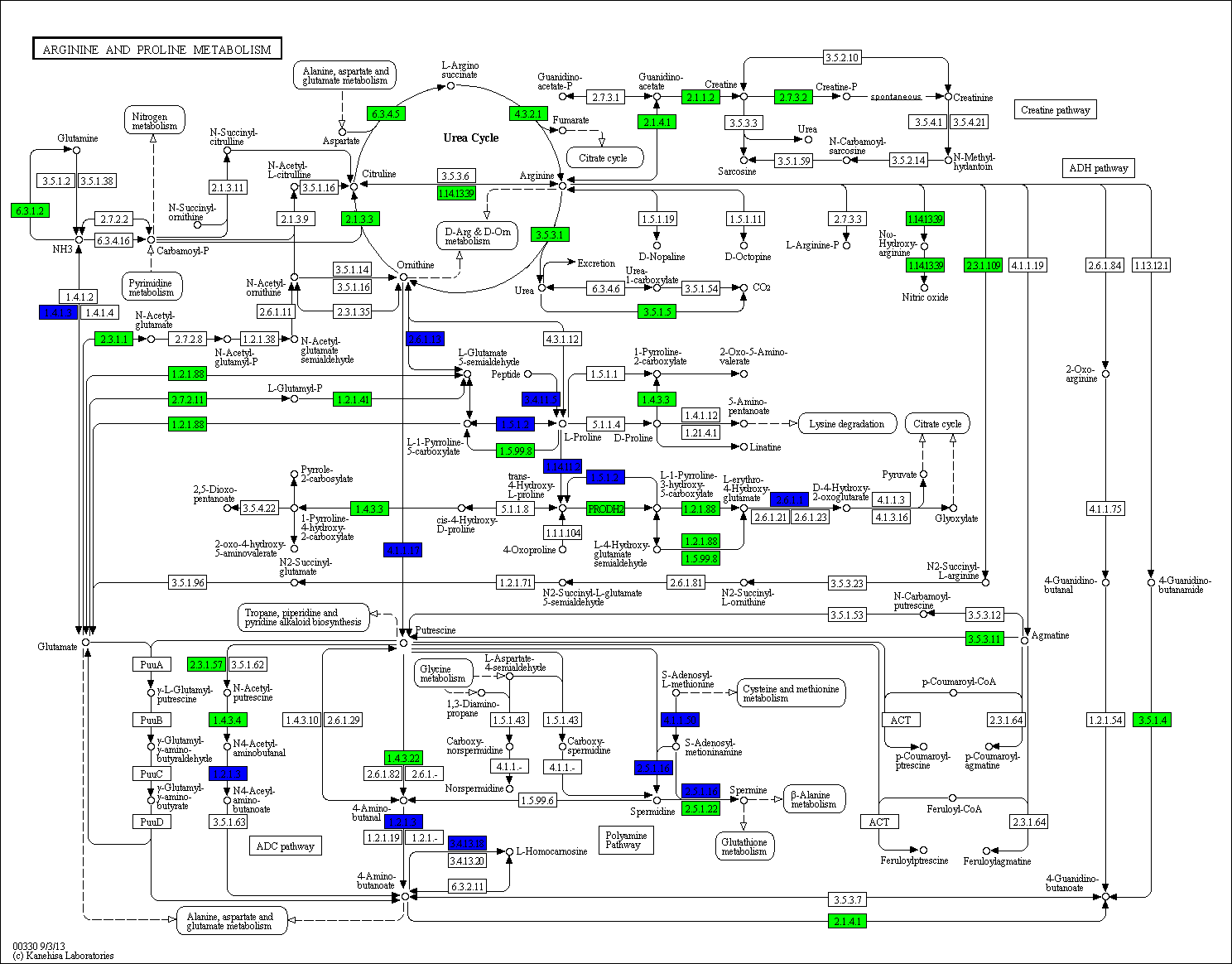


**D9 Histidine metabolism:**

*T. kitauei* is incapable of biosynthesis or degradation of histidine.


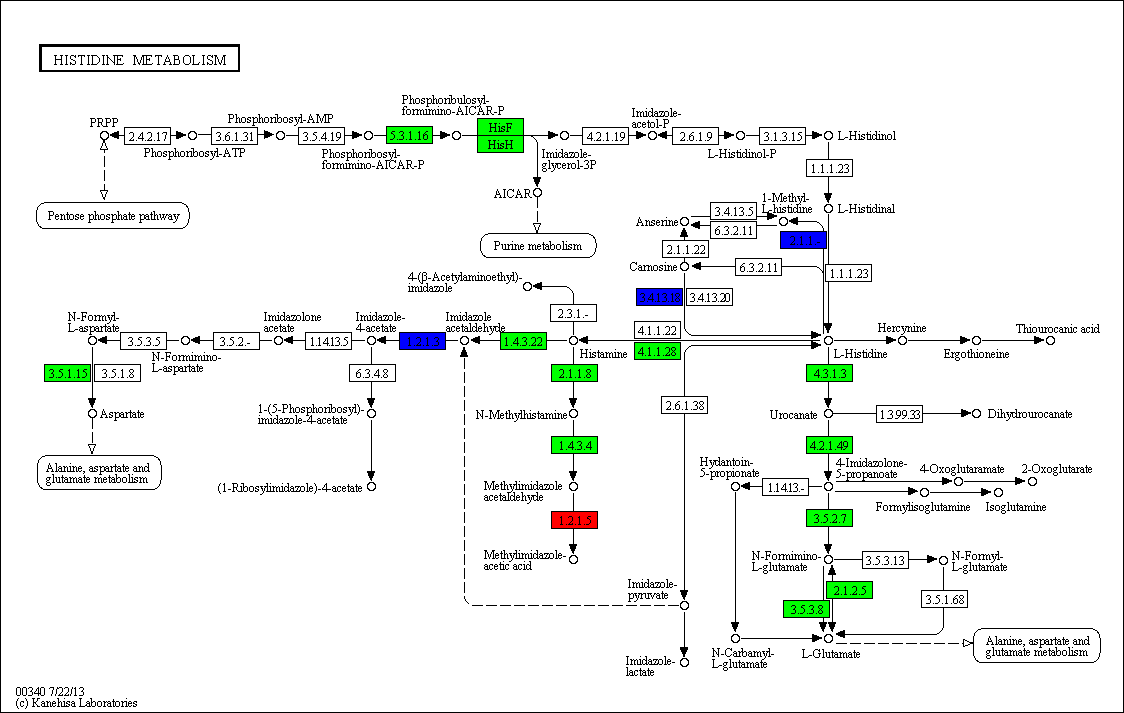


**D10 Tyrosine metabolism:**

*H. magnipapillata* and *N. vectensis* can break down tyrosine to acetoacetate.


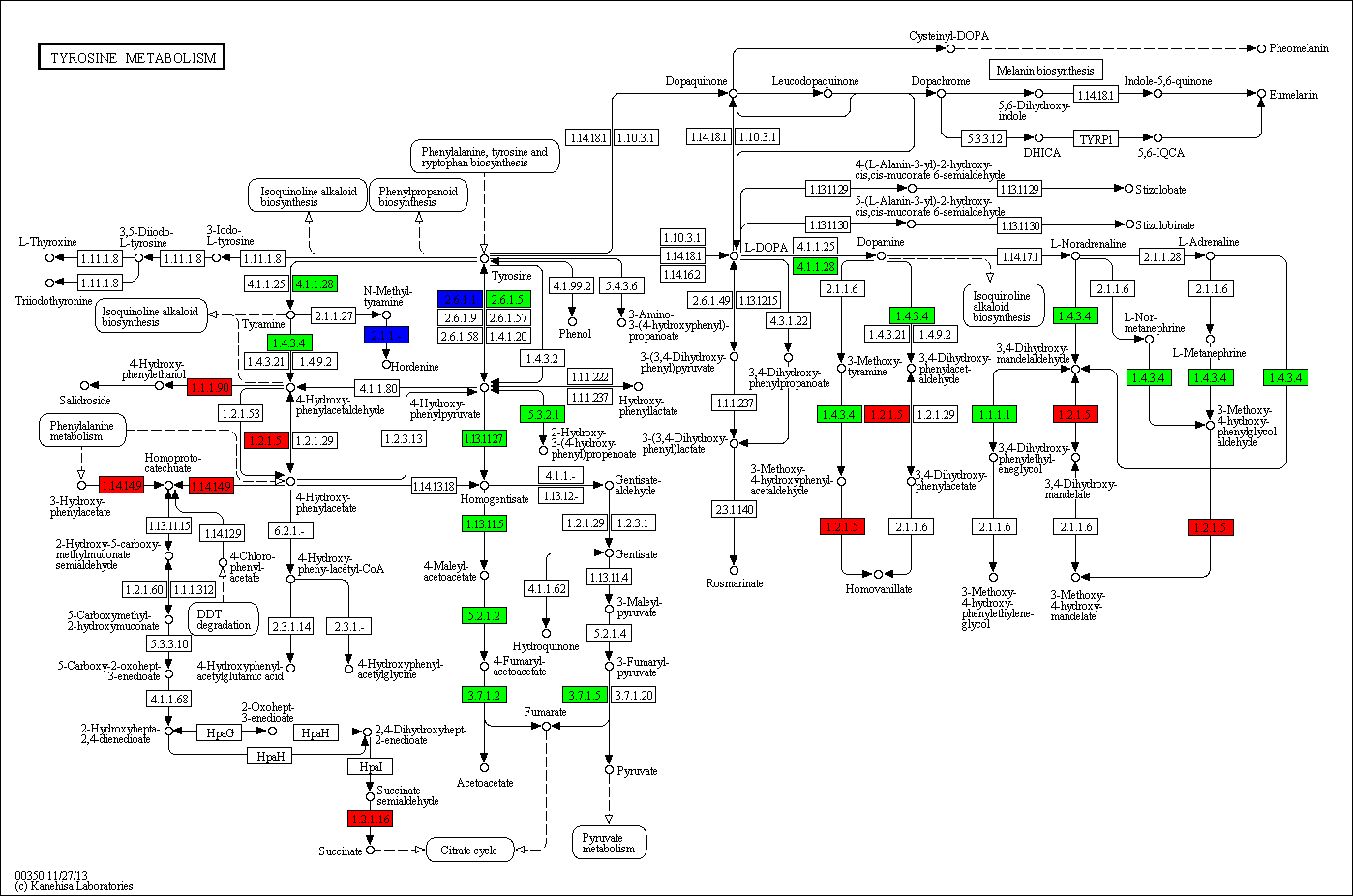


**D11 Phenylalanine metabolism:**

Phenylalanine is an essential amino acid for all three cnidarians. The two free-living cnidarians have the ability to convert phenylalanine to tyrosine. *T. kitauei*, however, does not have this enzyme and is auxotrophic for tyrosine. All three species can convert phenylalanine into phenylpyruvate.


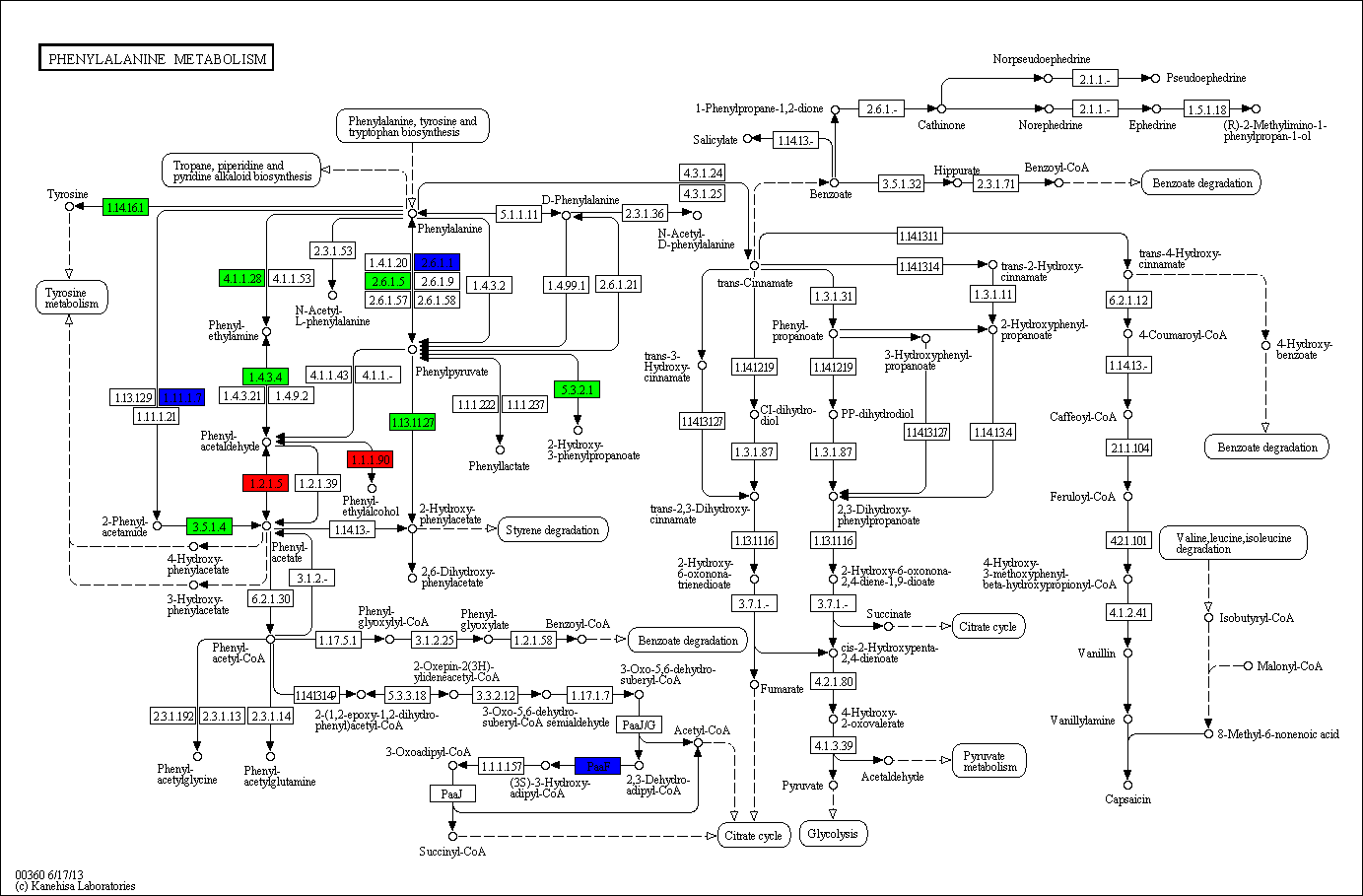


**D12 Tryptophan metabolism:**

Unlike *T. kitauei*, the two free-living species are capable of metabolizing tryptophan through the serotonin, tryptamine, and kynurenine pathways.


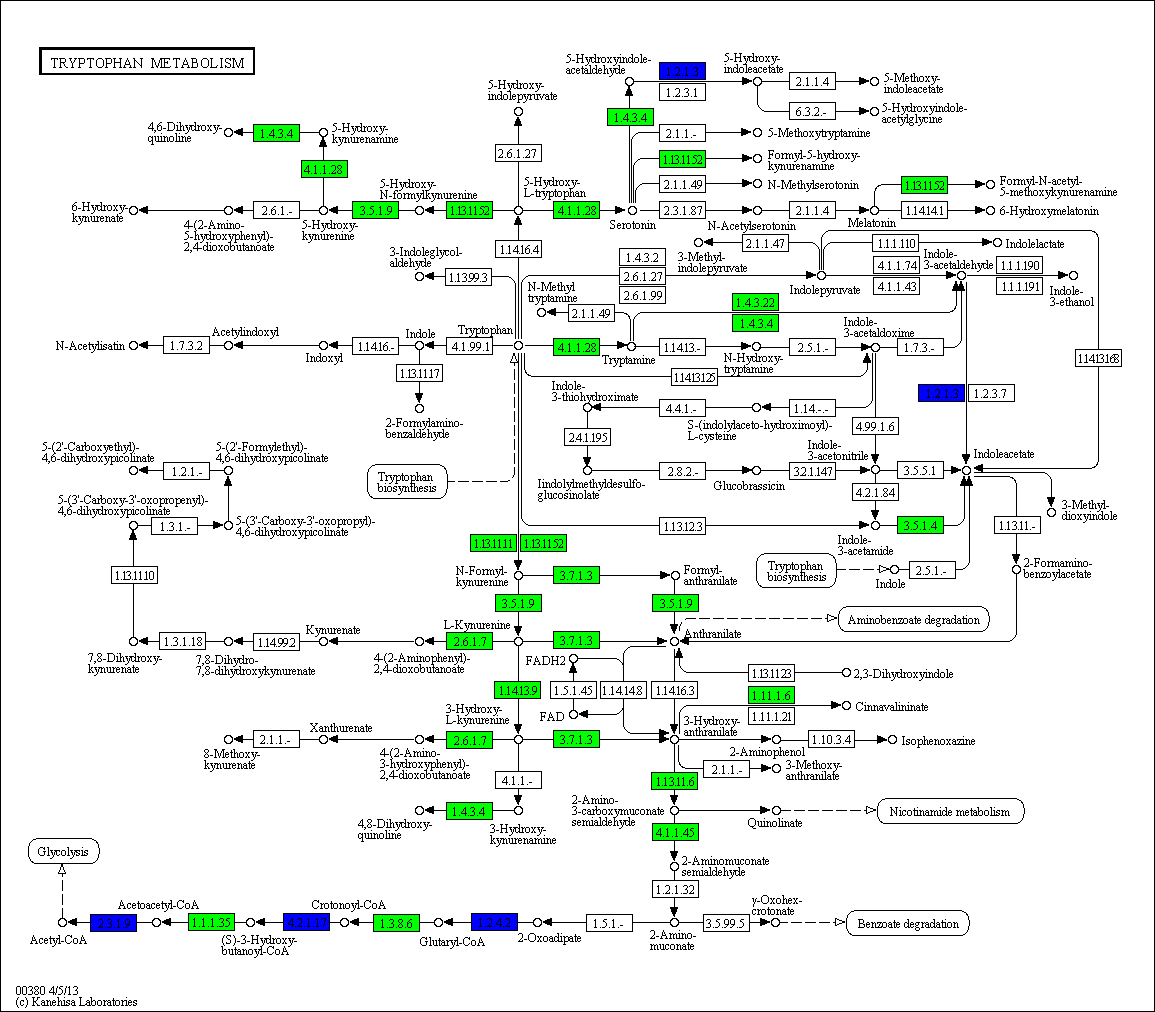


**D13 Phenylalanine, tyrosine and tryptophan biosynthesis:**

All three species have partially enzymes for phenylalanine, tyrosine and tryptophan biosynthesis.


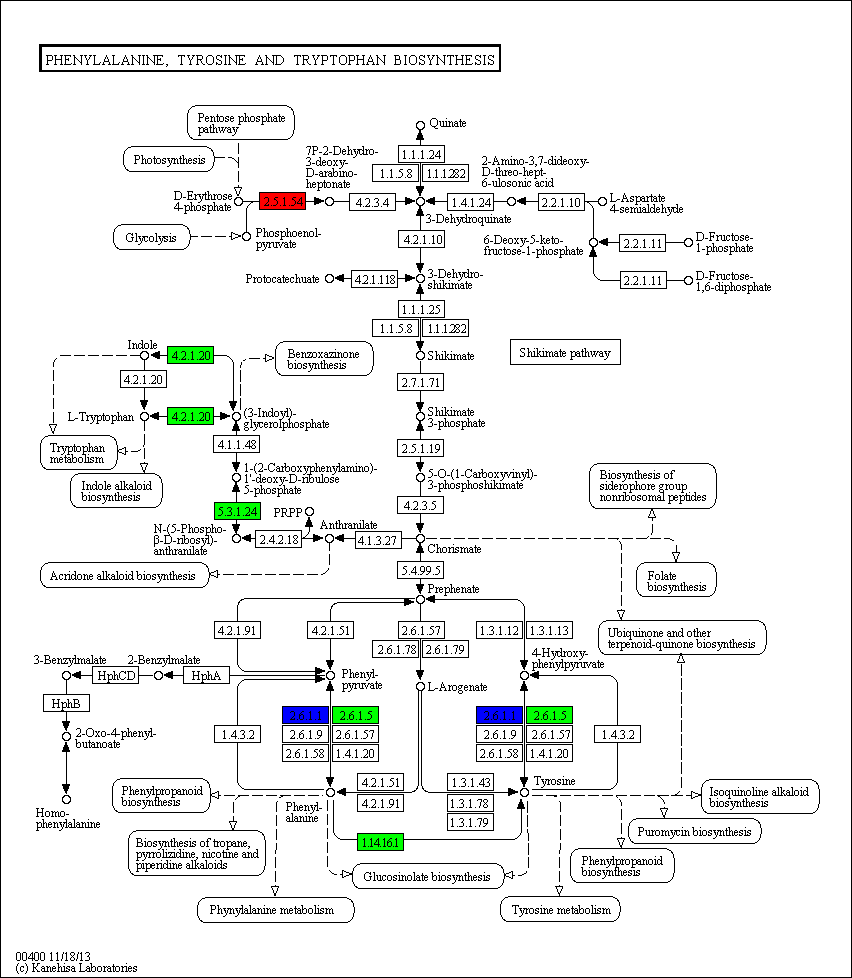


***E Lipid Metabolism***

**E1 Fatty acid elongation in mitochondria:**

All three species are capable of fatty acid elongation.


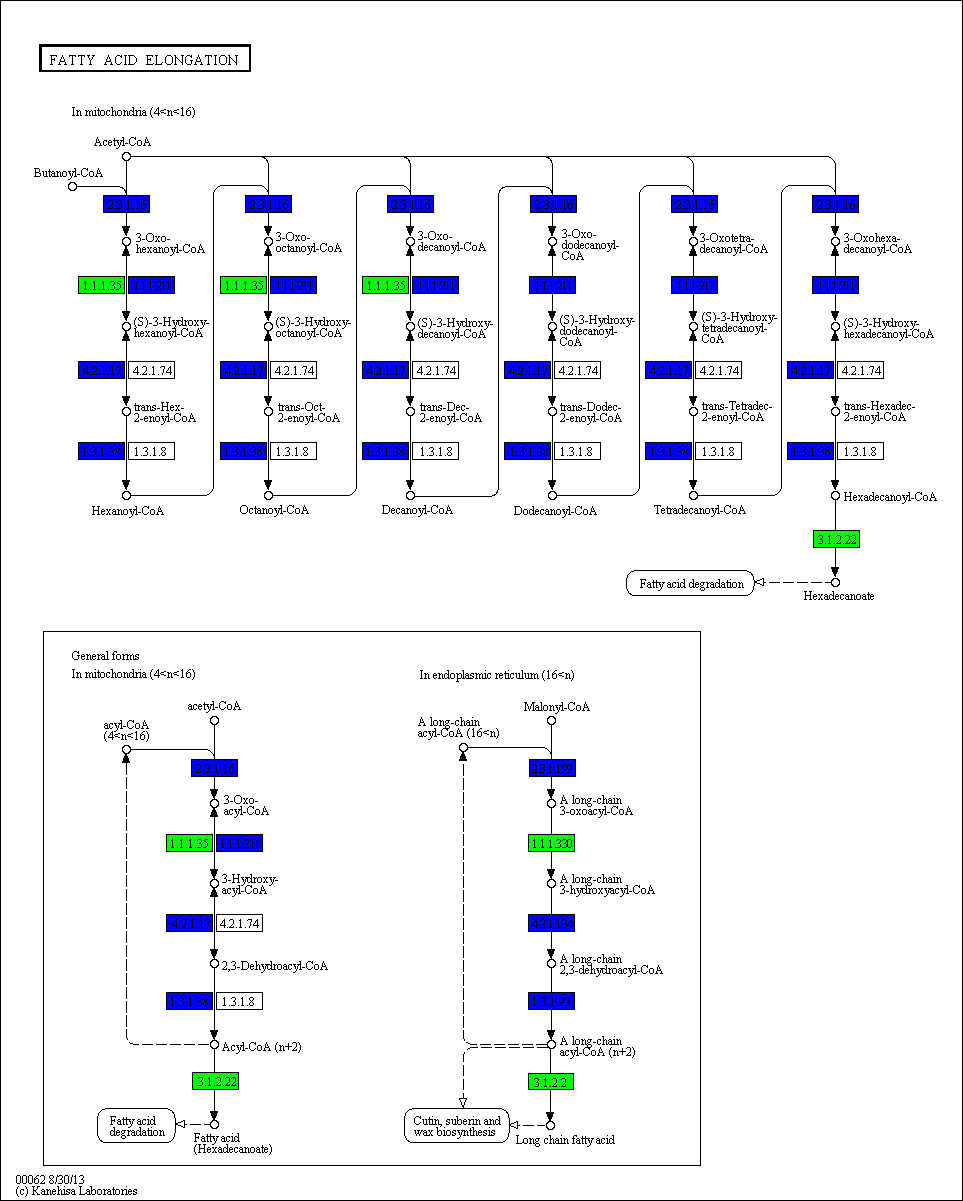


**E2 Fatty acid biosynthesis:**

All three species lack pathways for fatty acid biosynthesis.


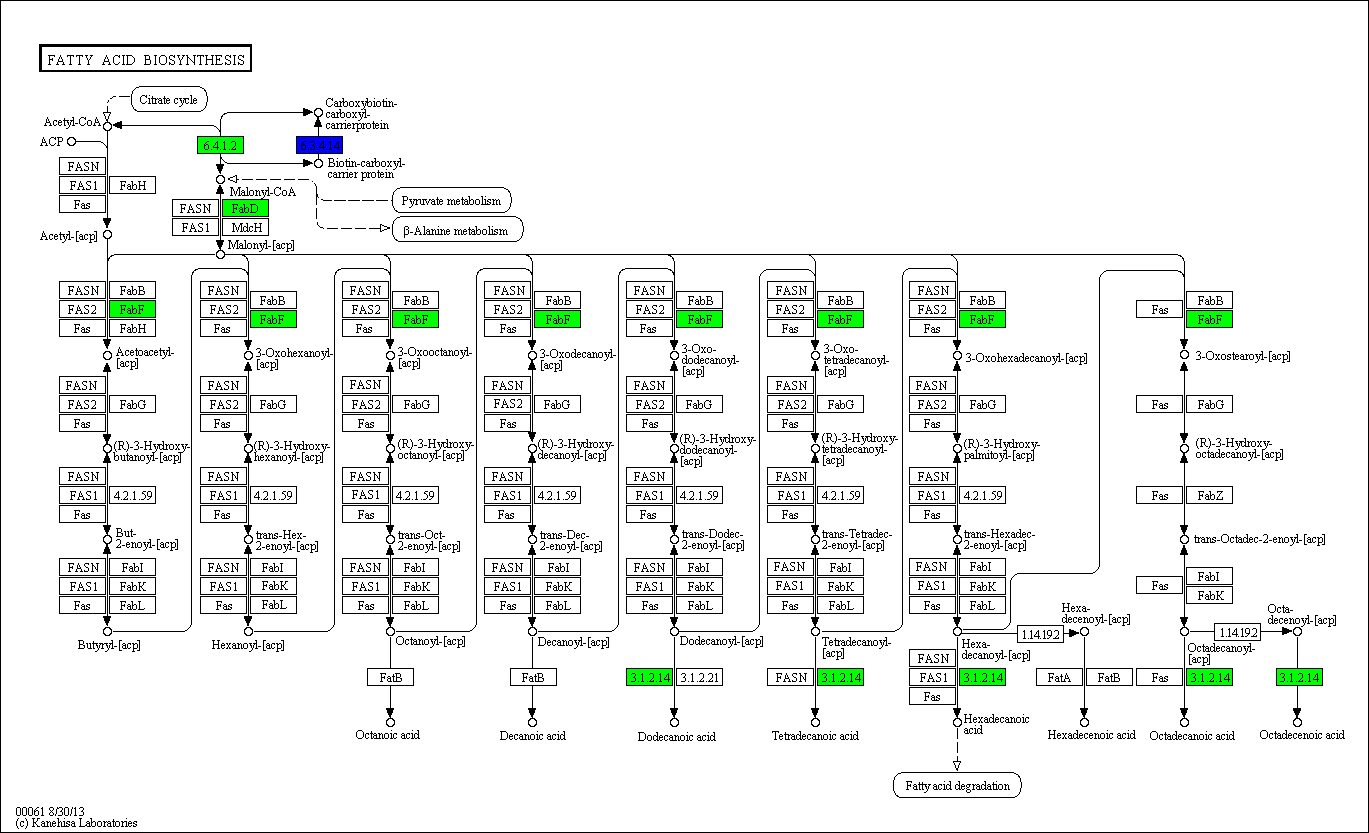


**E3 Fatty acid metabolism:**

All three species have some of the enzymes required for fatty acid breakdown via β-oxidation. *T. kitauei* lacks acyl-CoA dehydrogenases specifying either medium- or short-chain acyl-CoAs, which catalyze the first reaction of β-oxidation.


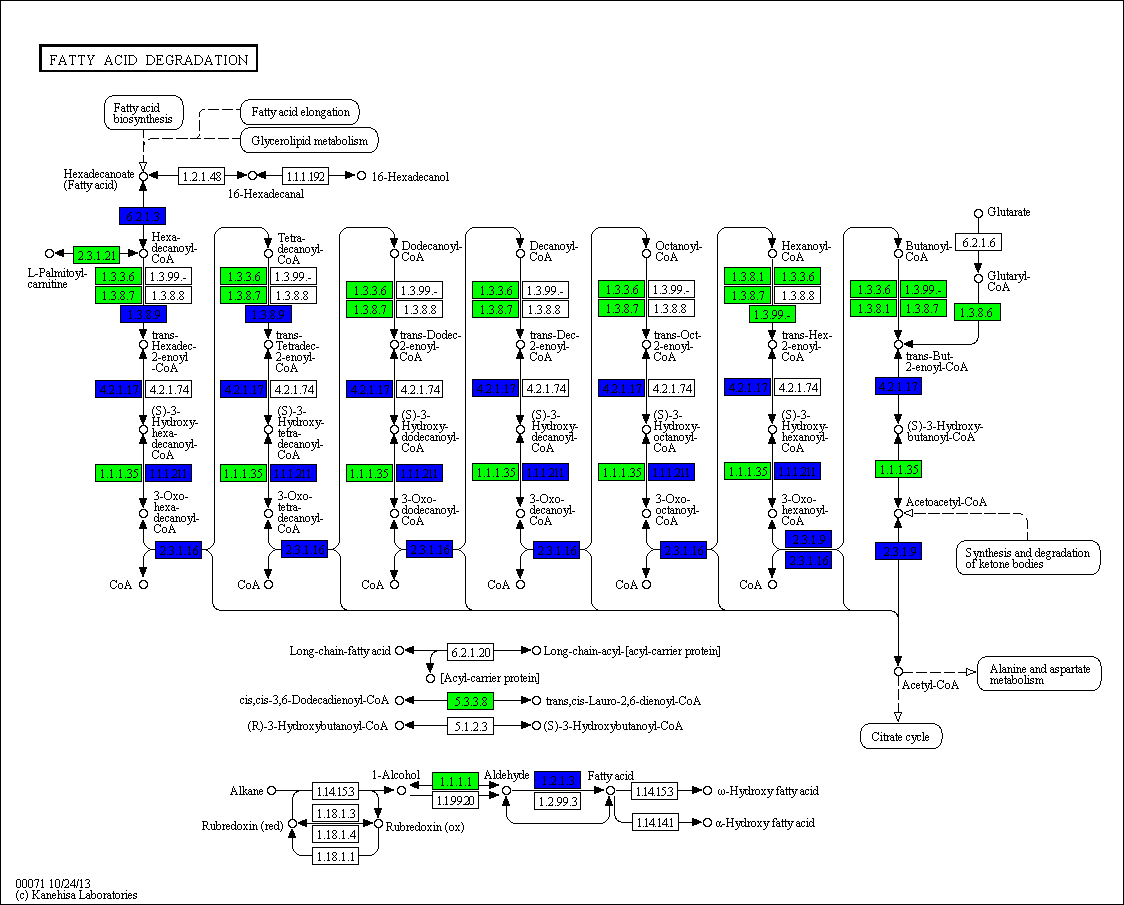


**E4 Synthesis and degradation of ketone bodies:**

*N. vectensis* can synthesis and degrade of ketone bodies.


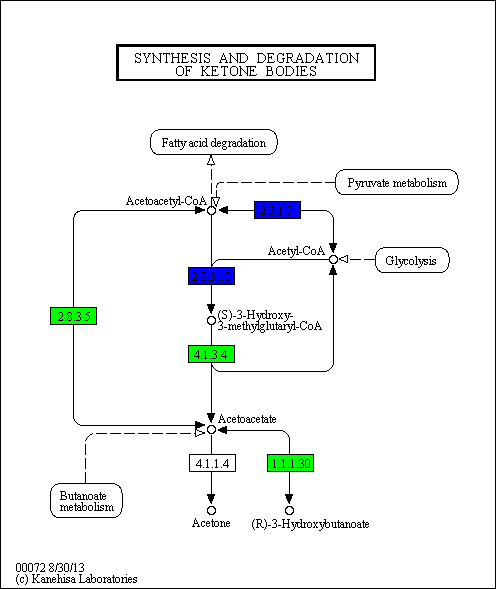


**E5 Steroid biosynthesis:**

All three species can synthesize cholesterol from 7-dehydrocholesterol or lathosterol and can esterify cholesterol to cholesteryl esters.


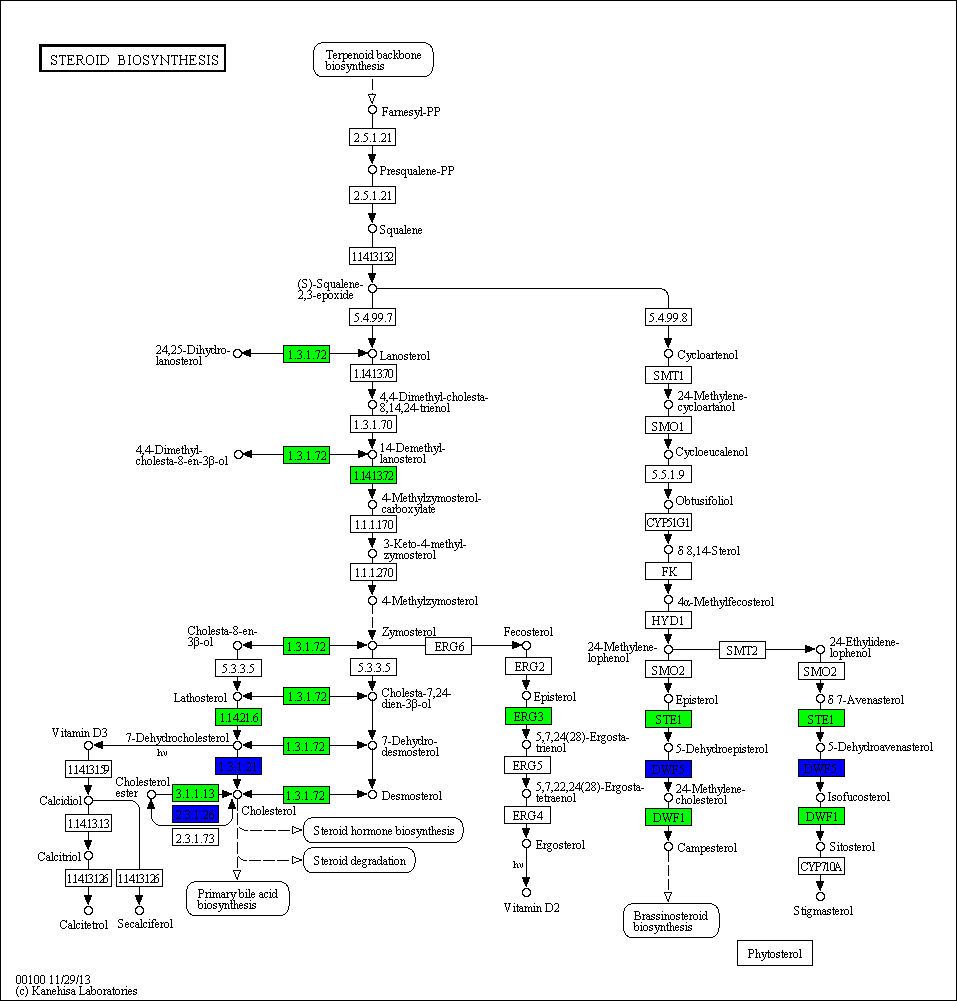


**E6 Glycerolipid metabolism:**

All three species have similar synthesize pathways for mono-, di- and triacylglycerol. *T. kitauei* mediates [tri- and diacylglycerol](http://en.wikipedia.org/wiki/Triacylglycerol) hydrolysis by patatin-like phospholipase domain-containing protein 3. *N. vectensis* mediates monoacylglycerol hydrolysis by acylglycerol lipase.


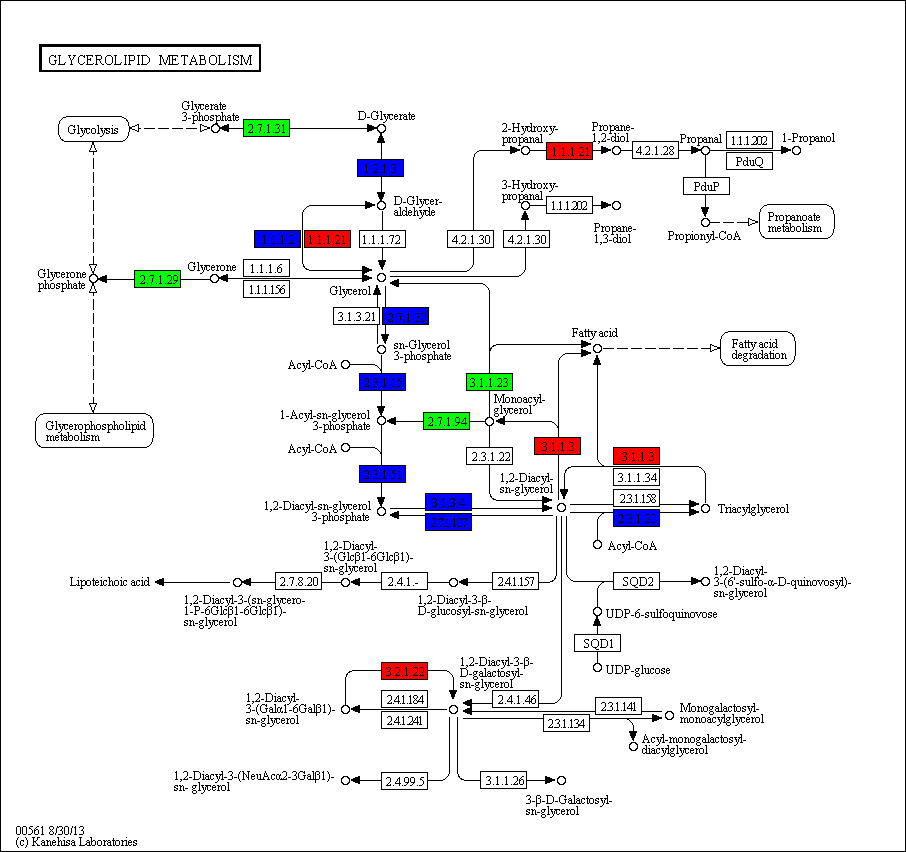


**E7 Glycerophospholipid** **metabolism:**

No major differences were observed between the three species. All three cnidarians can synthesize glycerophospholipids such as phosphatidylcholine, phosphatidylserine and phosphatidylethanolamine.

In *T. kitauei*, phosphatidylcholine is synthesized by the diacylglycerol pathway, and phosphatidylserine is produced from phosphatidylcholine by base-exchange reactions. Glycerophospholipids are synthesized by the phosphatidylserine decarboxylation pathway. sn-Glycero-3-phosphocholine and sn-glycero-3-phosphoethanolamine are the major forms of choline and ethanolamine storage, respectively, in the cytosol.


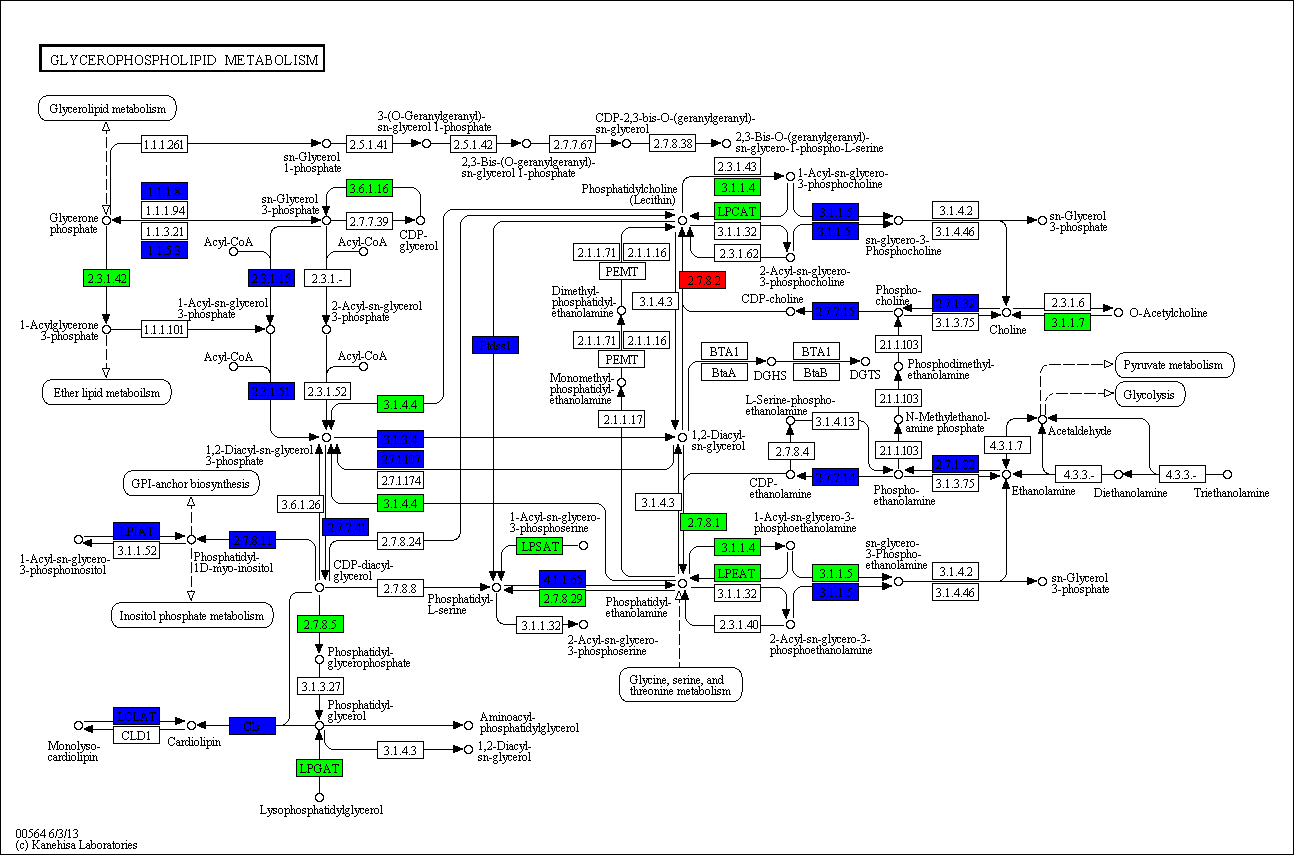


**E8** [**Arachidonic acid**](http://en.wikipedia.org/wiki/Arachidonic_acid) **metabolism:**

All three cnidarians can degrade [arachidonic acid](http://en.wikipedia.org/wiki/Arachidonic_acid) to produce leukotrienes.


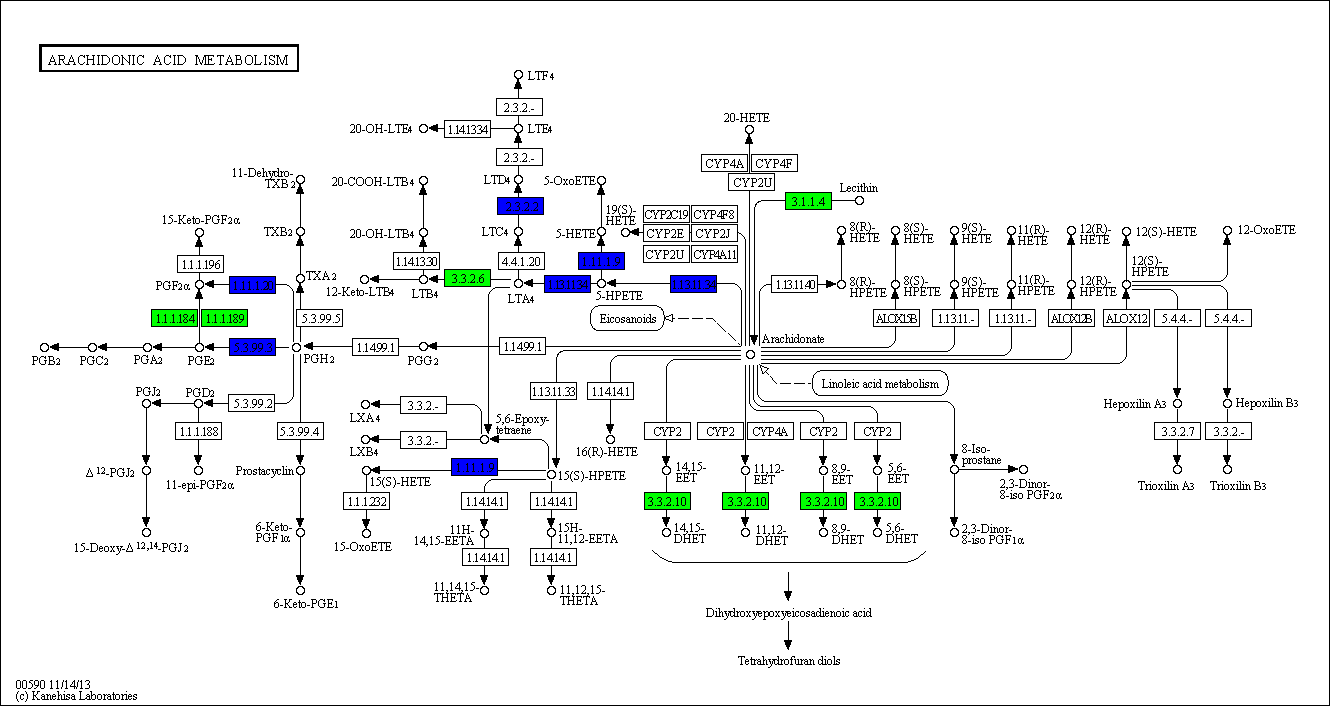


**E9 Sphingolipid metabolism:**

Compared with its two free-living relatives, *T. kitauei* has reduced metabolism of sphingolipids. The two free-living cnidarians are capable of *de novo* synthesis of ceramide, and ceramide can be further reversibly converted to ceramide-1-phosphate, glucosylceramide, sphingomyelin or sphingosine. *T. kitauei* lacks dihydroceramide desaturase, which can desaturate dihydroceramide to form ceramide, but ceramide can be formed from sphingosine by reacylation or be created from sphingomyelin by sphingomyelinase. Ceramide may be subsequently metabolized to sphingomyelin or glucosylceramide.


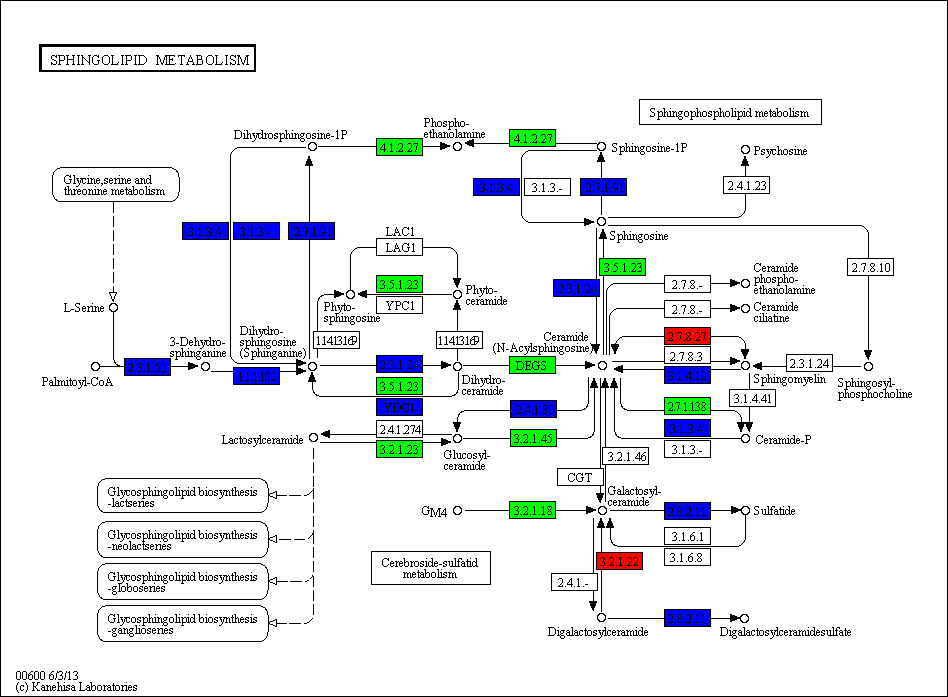


***F Nucleotide Metabolism***

**F1 Purine metabolism:**

Unlike the two free-living species, *T. kitauei* is not capable of synthesizing purines, depending instead on salvage reactions. *T. kitauei* purine metabolism is greatly simplified, retaining only the purine nucleotide cycle, including the conversion of IMP to AMP or XMP and interconversion between IMP and GMP. Lacking hypoxanthine-guanine and xanthine phosphoribosyltransferase and adenosine deaminase, the parasite may produce GMP from free guanine by adenine phosphoribosyltransferase or from IMP by GMP synthase (glutamine-hydrolyzing) and IMP dehydrogenase. Unlike *T. kitauei and H. magnipapillata*, *N. vectensis* can further degrade the purine bases to uric acid and urea.

*
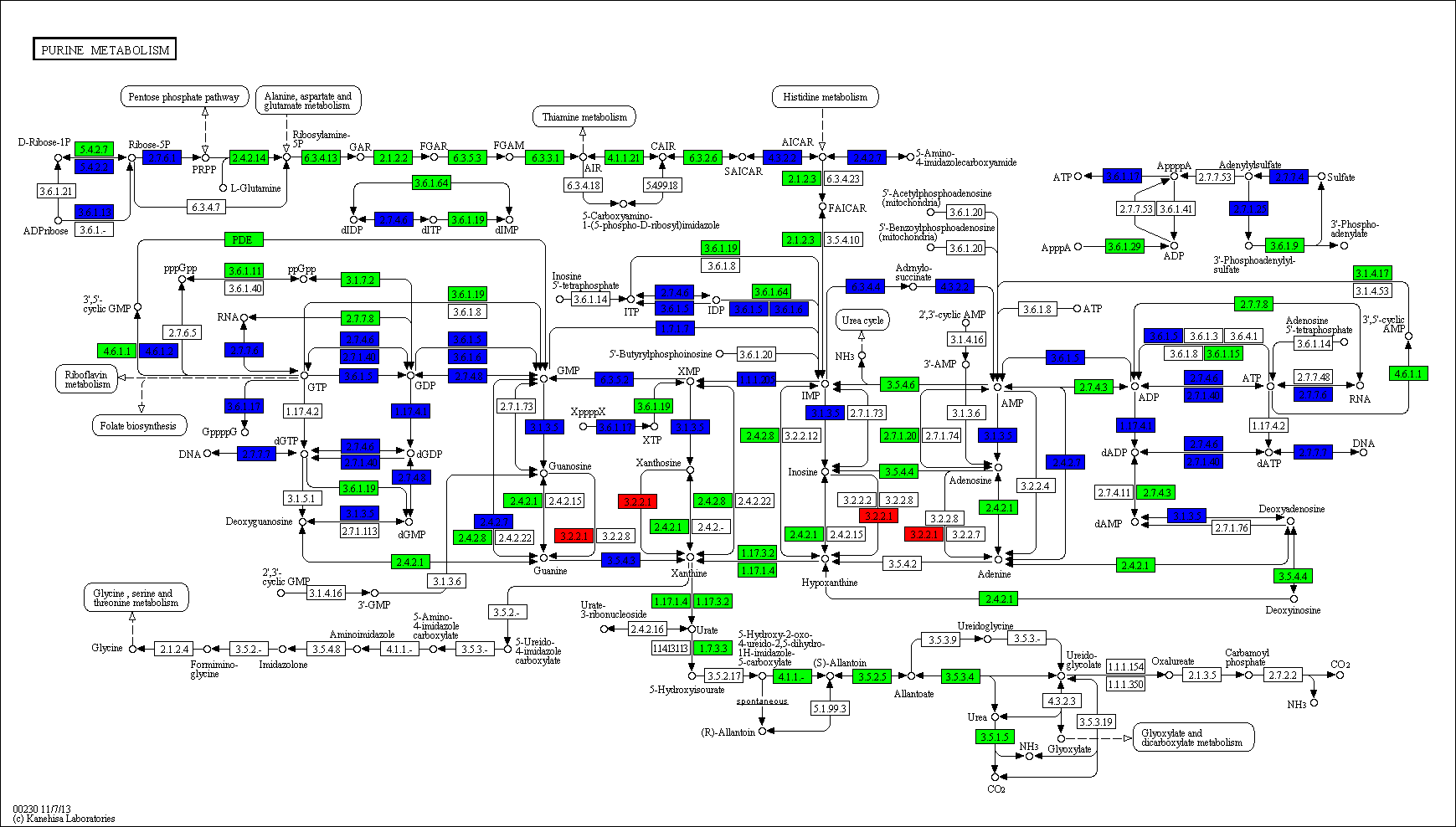
*

**F2 Pyrimidine metabolism:**

Unlike *H. magnipapillata* and *N. vectensis*, *T. kitauei* is incapable of synthesizing pyrimidines and depends on pyrimidine salvage for survival. In the pyrimidine salvage pathway of *T. kitauei*, uridine, cytidine, deoxyuridine and thymidine can be reconverted to nucleotides via the salvage pathway. dTMP can also be synthesized from dUMP by thymidylate synthase. UTP is aminated by CTP synthase, generating CTP. In *T. kitauei*, all dNDPs are derived from NDPs via the catalysis of a common ribonucleotide reductase, likely via a 3′-ribonucleotide radical intermediate. *T. kitauei* lacks the enzymes that degrade free bases of purines and pyrimidines.


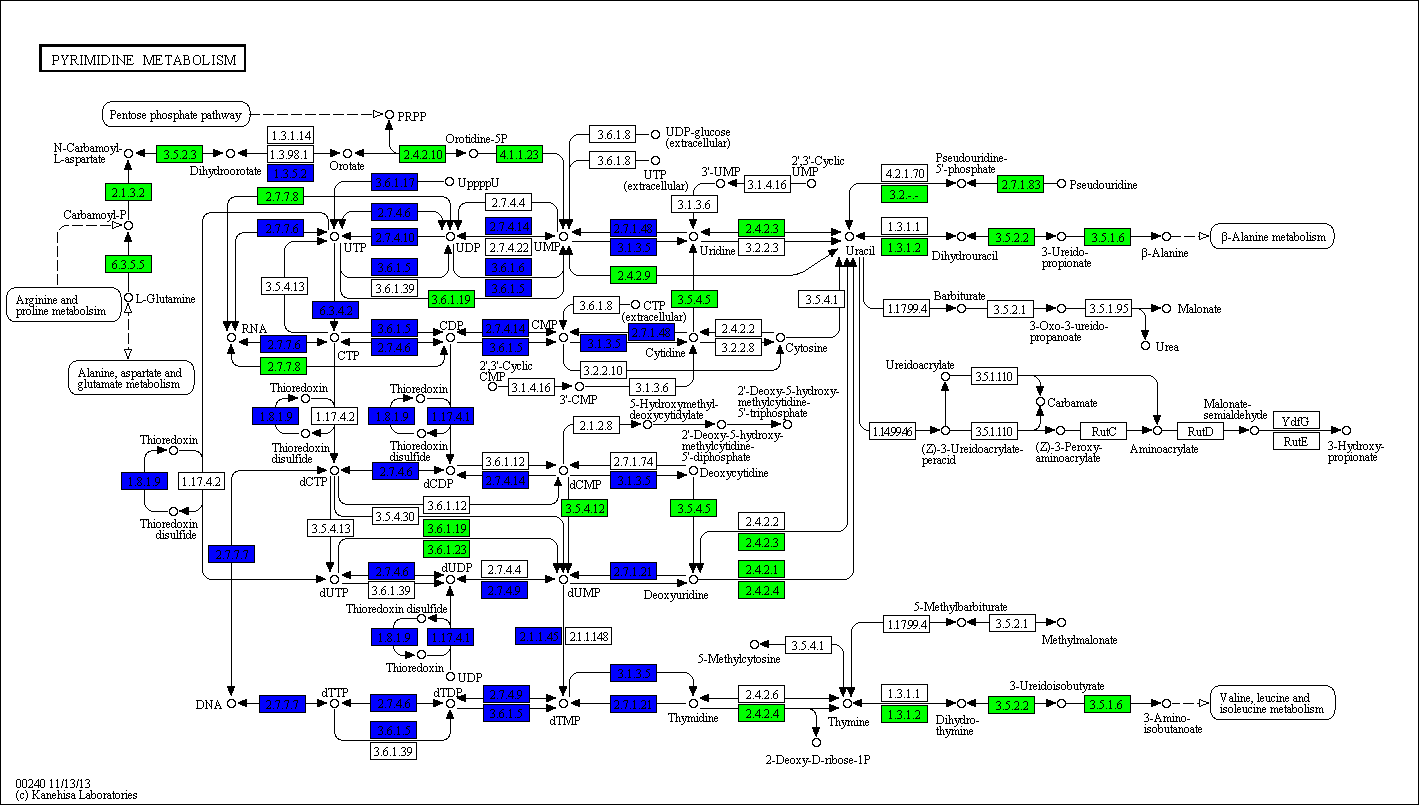


***G Metabolism of Cofactors and Vitamins***

**G1 Ubiquinone and other terpenoid-quinone biosynthesis:**

No major differences were seen between species. All three species can synthesize CoQ from polyprenyl-PP.


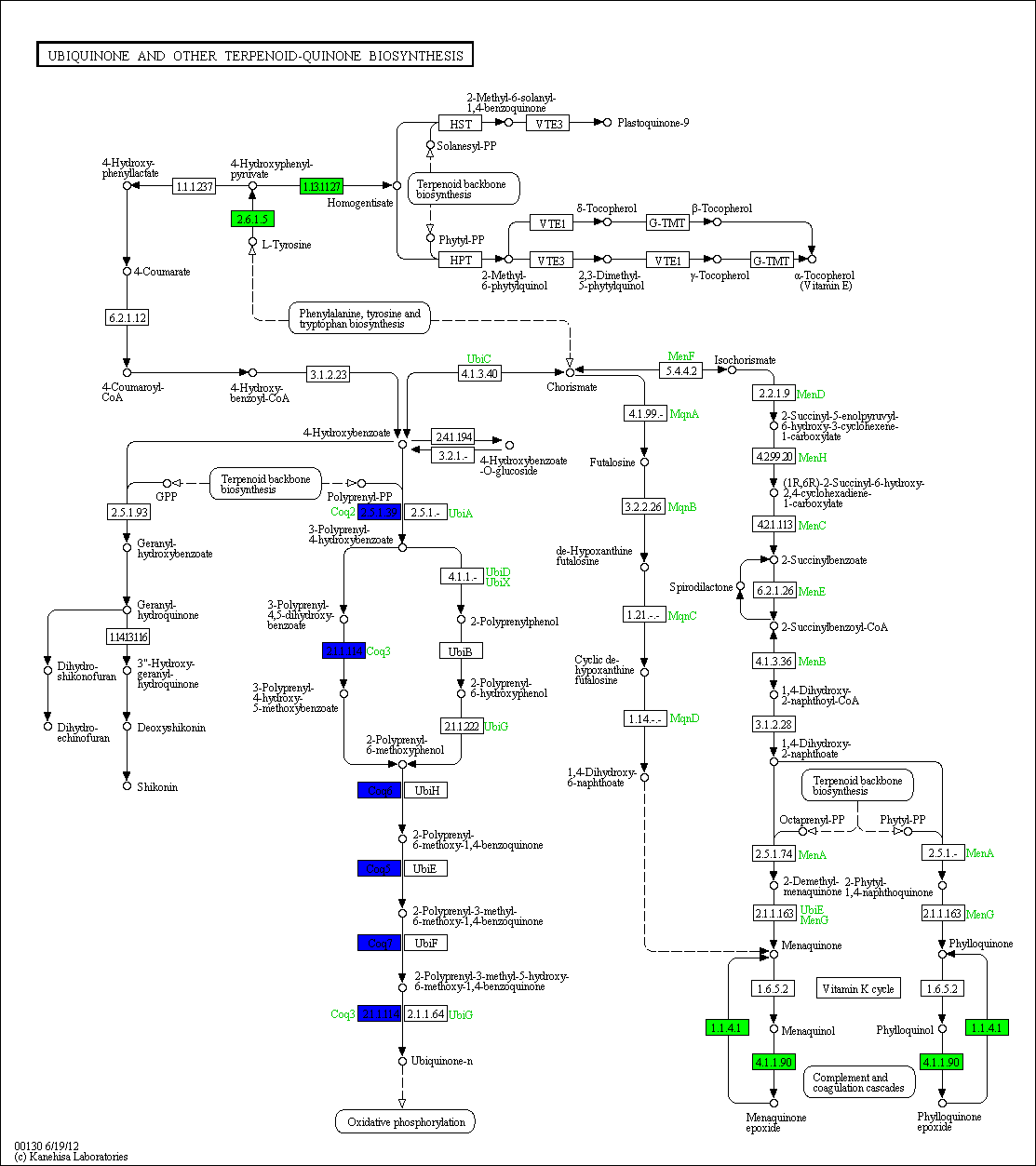


**G2 One-carbon pool by folate:**

All three species can transfer 1C units to tetrahydrofuran to generate 5,10-methylene-tetrahydrofuran, which is required for *de novo* thymidylate synthesis, and may also be reduced to 5-methyl-tetrahydrofuran (for methyl group biogenesis in the two free-living species) or oxidized to 10-formyl-tetrahydrofuran, which is required for purine biosynthesis. *T. kitauei* lacks dihydrofolate reductase, which reduces folic acid to 5,6,7,8-tetrahydrofolate.


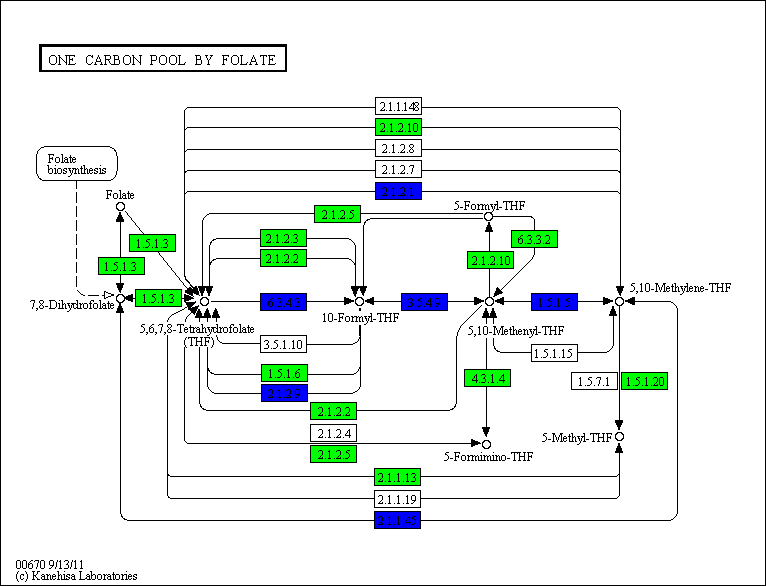


**G3 Pantothenate and CoA biosynthesis:**

*T. kitauei* can convert phosphopantothenoylcysteine or pantetheine to CoA. The two free-living cnidarians can convert pantothenate to CoA.

**
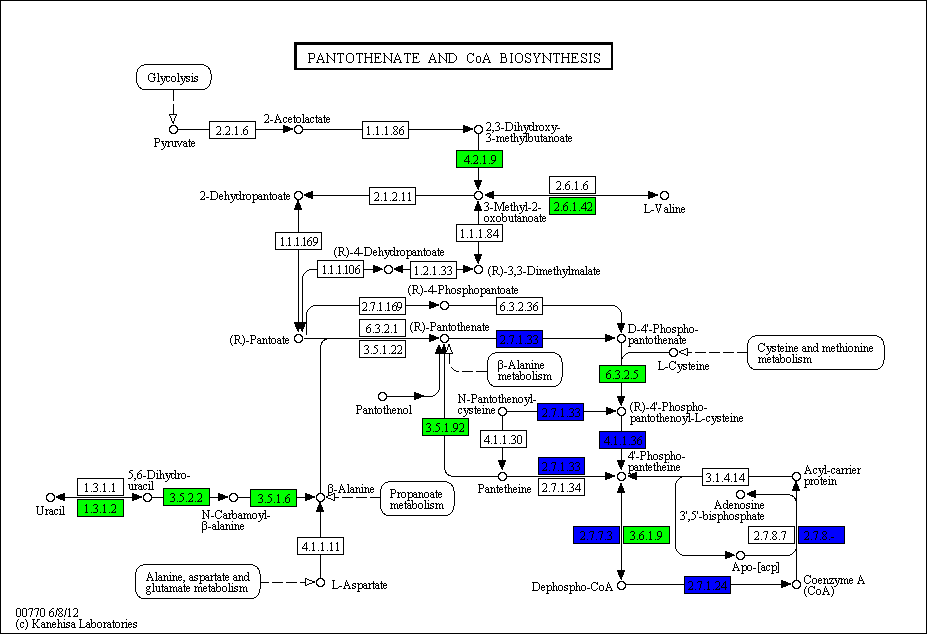
**

**G4 Lipoic acid metabolism:**

The three species have the lipoate synthesis pathway, which can produce protein-bound lipoate from an octanoylated precursor.


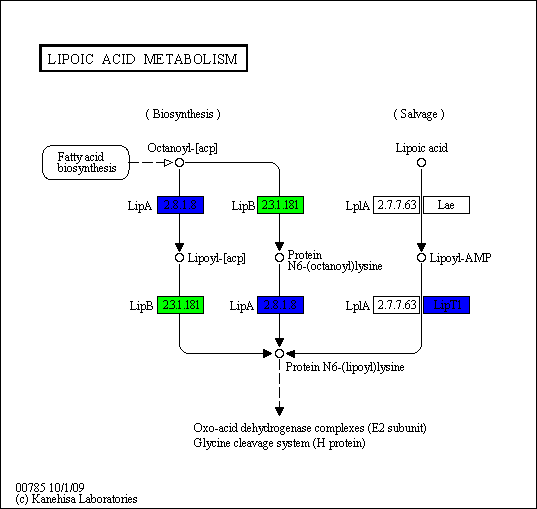


**G5 Retinol metabolism in animals:**

In the two free-living cnidarians, retinyl ester can be reversibly converted to retinol then to retinal, and retinal (retinaldehyde) can be irreversibly oxidized to produce retinoic acid.


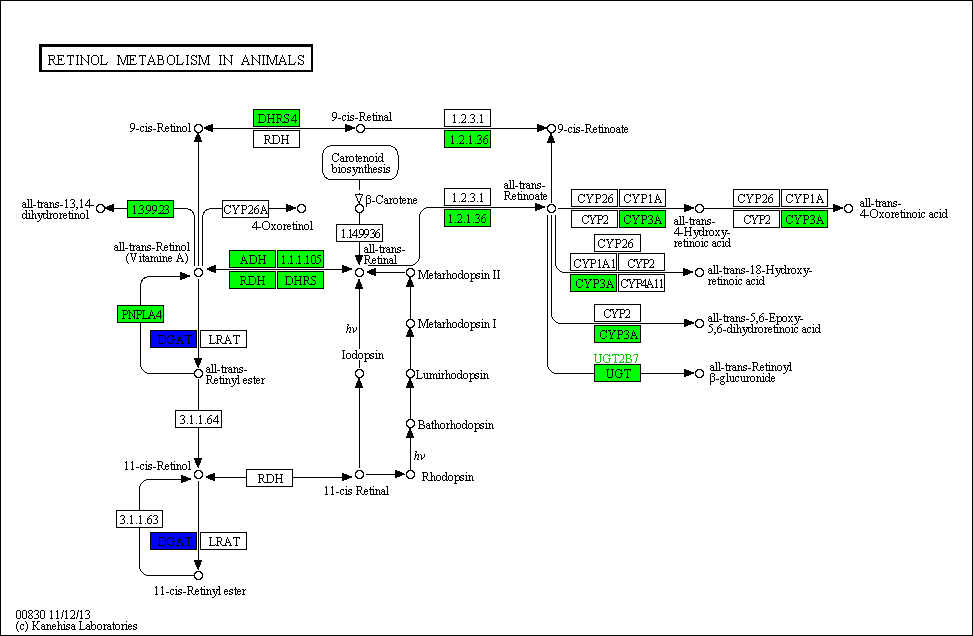


**G6 Porphyrin and chlorophyll metabolism:**

All three species use the animal/fungal type C4 pathway for heme biosynthesis.


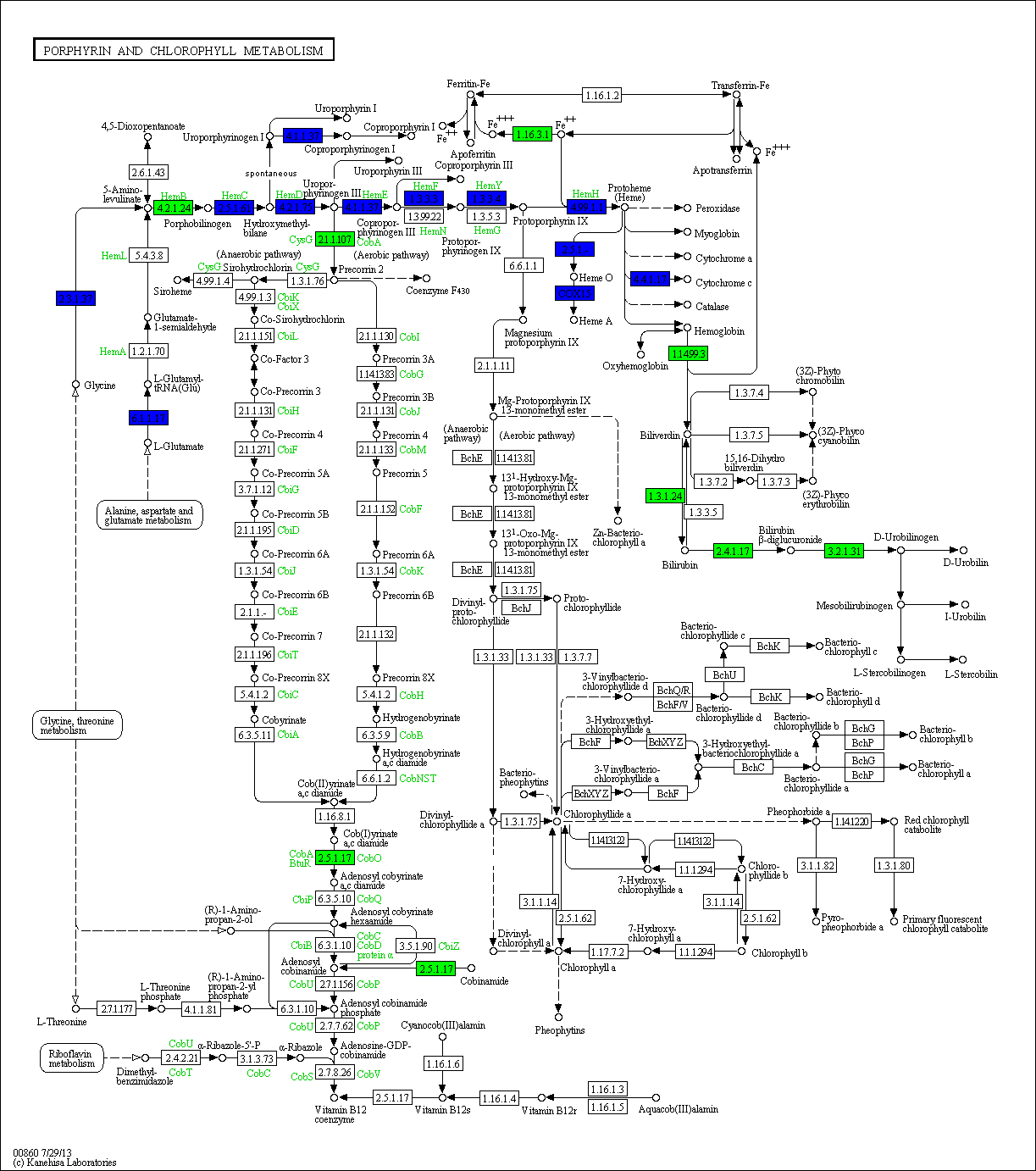


***H Glycan Biosynthesis and Metabolism***

**H1 N-Glycan biosynthesis:**

All three species are capable of N-glycan biosynthesis. The two free-living species have a more complete set of enzymes.


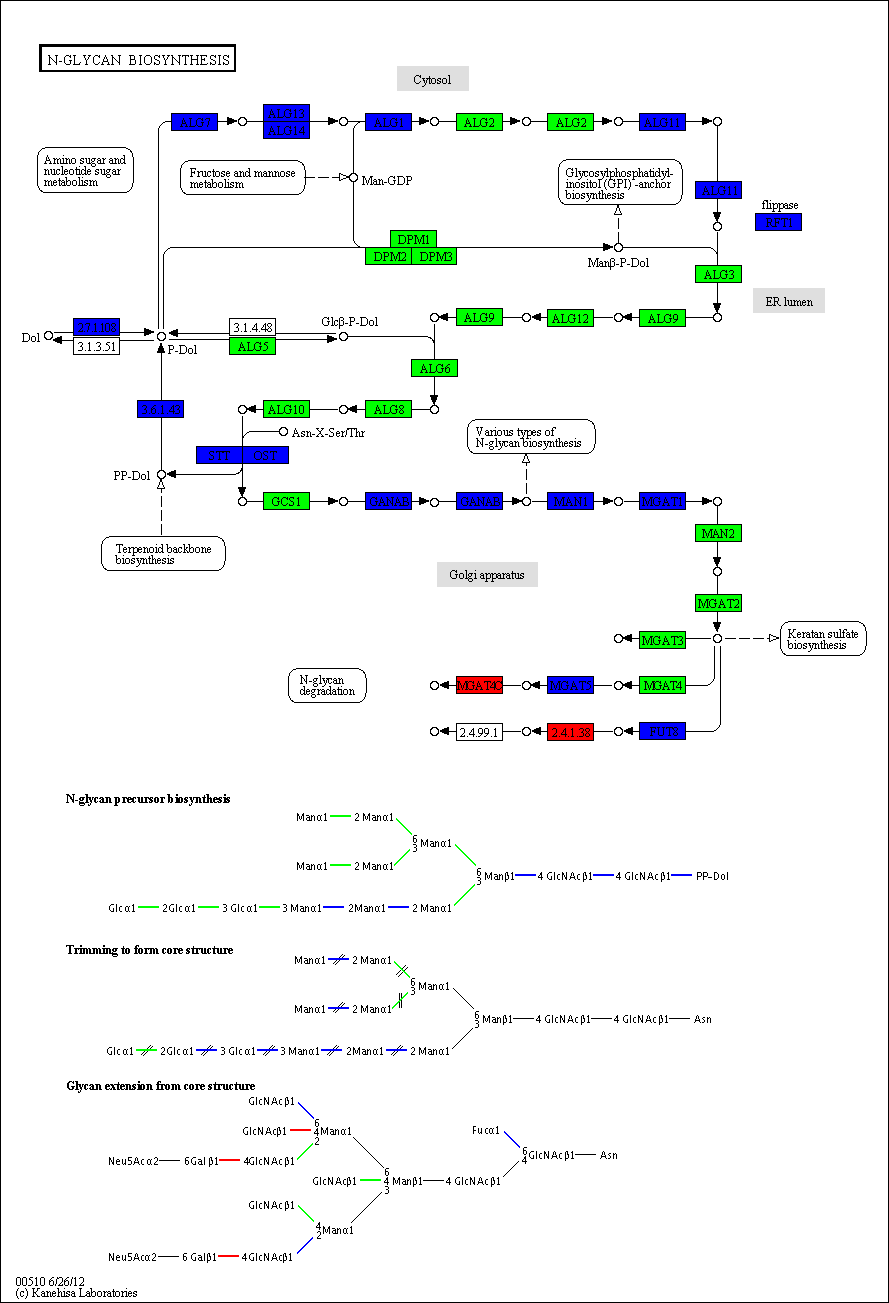


**H2 Other glycan degradation:**

The two free-living species contain all enzymes related to N-glycan and ganglioside degradation. *T. kitauei* has three enzymes that are involved in the catabolism of N-glycans of glycoproteins.


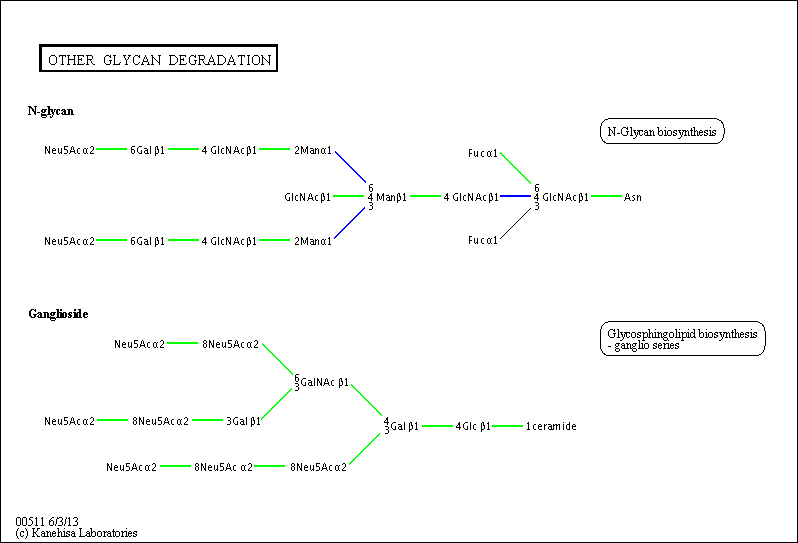


**H3 Mucin type O-glycan biosynthesis:**

All three species are capable of mucin type O-glycan biosynthesis. *T. kitauei* has only the enzyme that catalyzes the initial reaction in O-linked oligosaccharide biosynthesis, the transfer of an N-acetyl-D-galactosamine residue to a serine or threonine residue on the protein receptor.


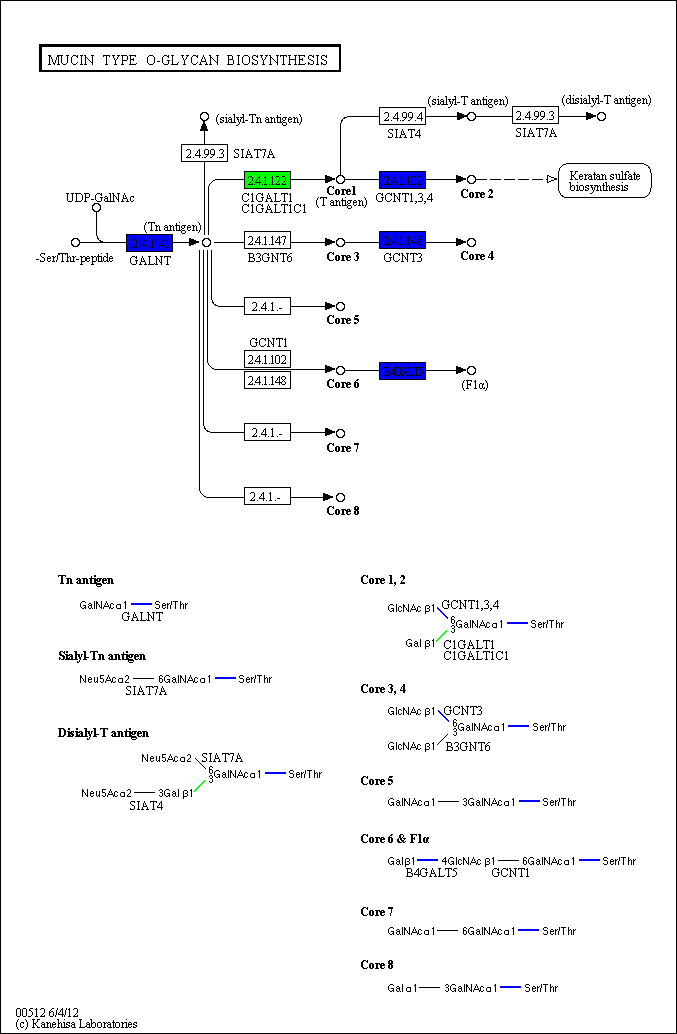


**H4 Various types of N-glycan biosynthesis:**

All three species are capable of synthesizing complex type, paucimannose type, and glycohormone N-glycans.


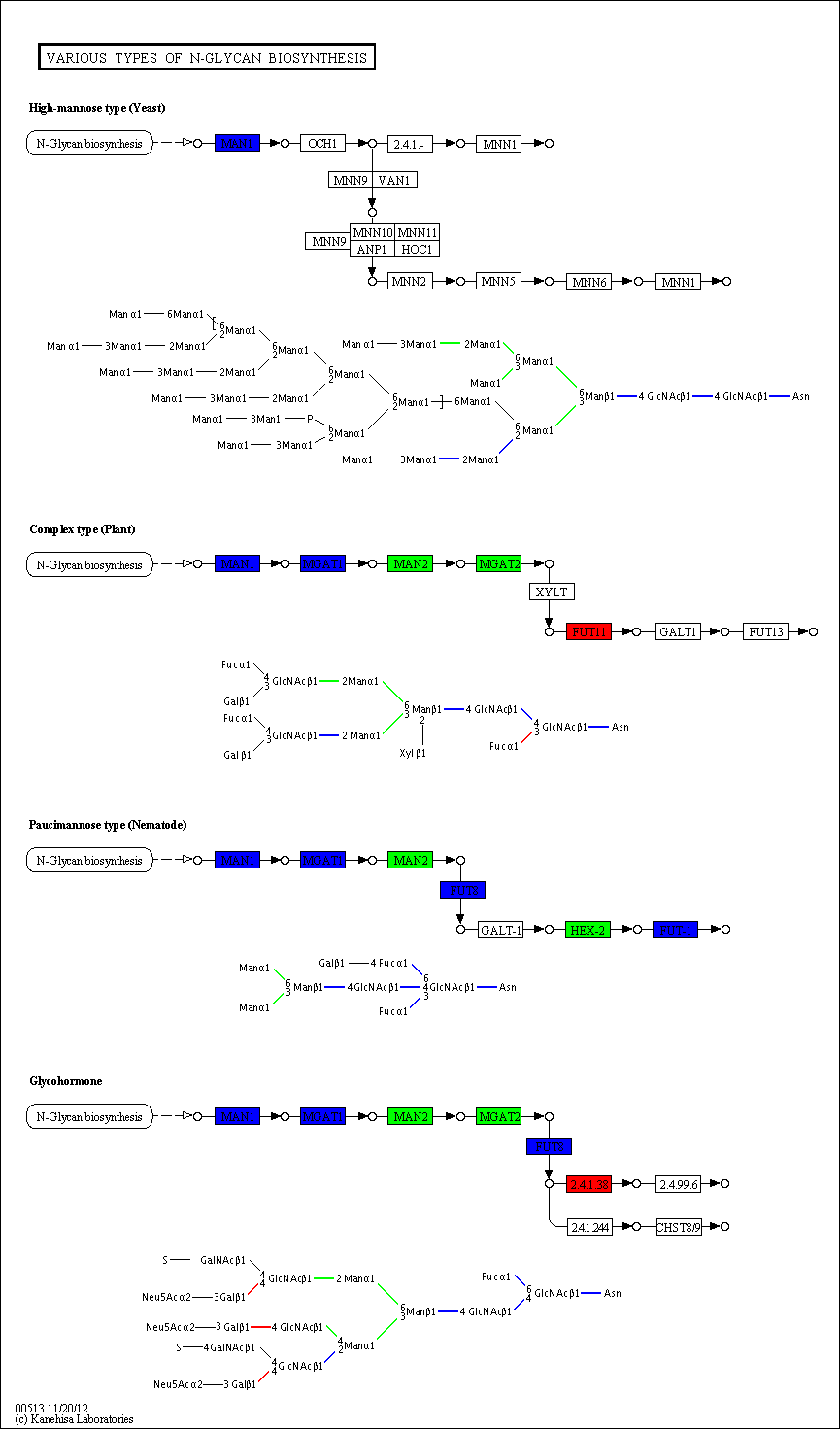


**H5 Glycosaminoglycan degradation:**

The two free-living species are capable of glycosaminoglycan degradation.


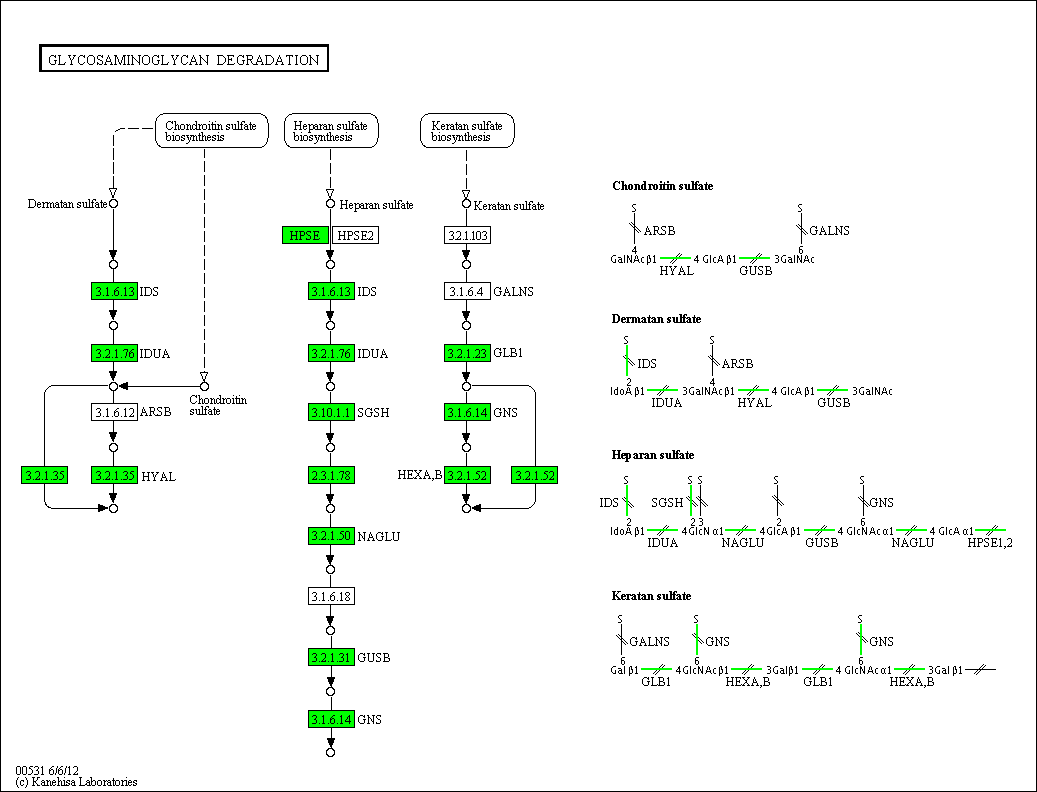


**H6 Glycosaminoglycan biosynthesis****—chondroitin sulfate/dermatan sulfate:**

All three species are partially capable of glycosaminoglycan biosynthesis—chondroitin sulfate/dermatan sulfate.


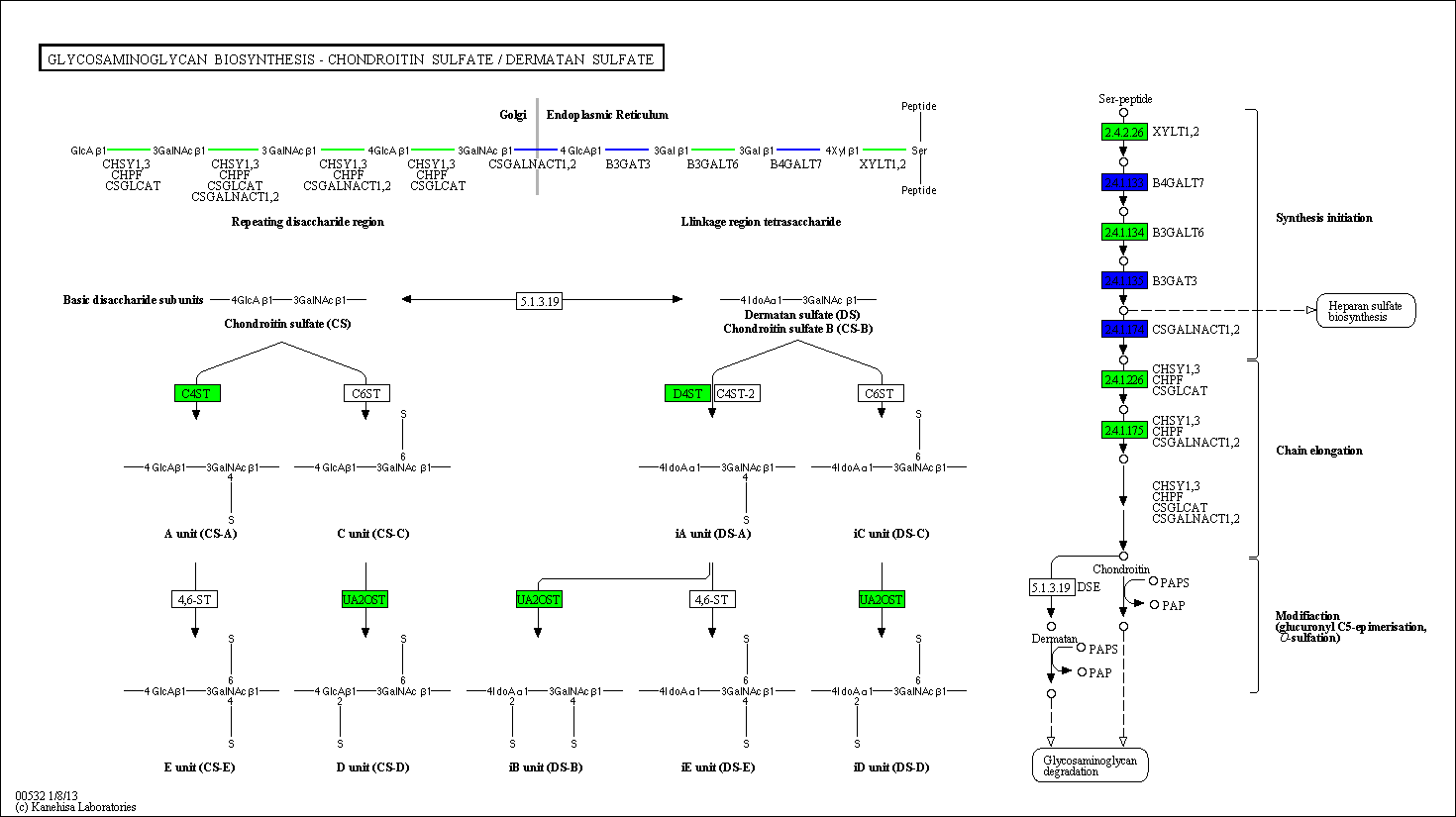


**H7 Glycosaminoglycan biosynthesis****—heparan sulfate/heparin:**

The two free-living species are capable of glycosaminoglycan biosynthesis—heparan sulfate/heparin.


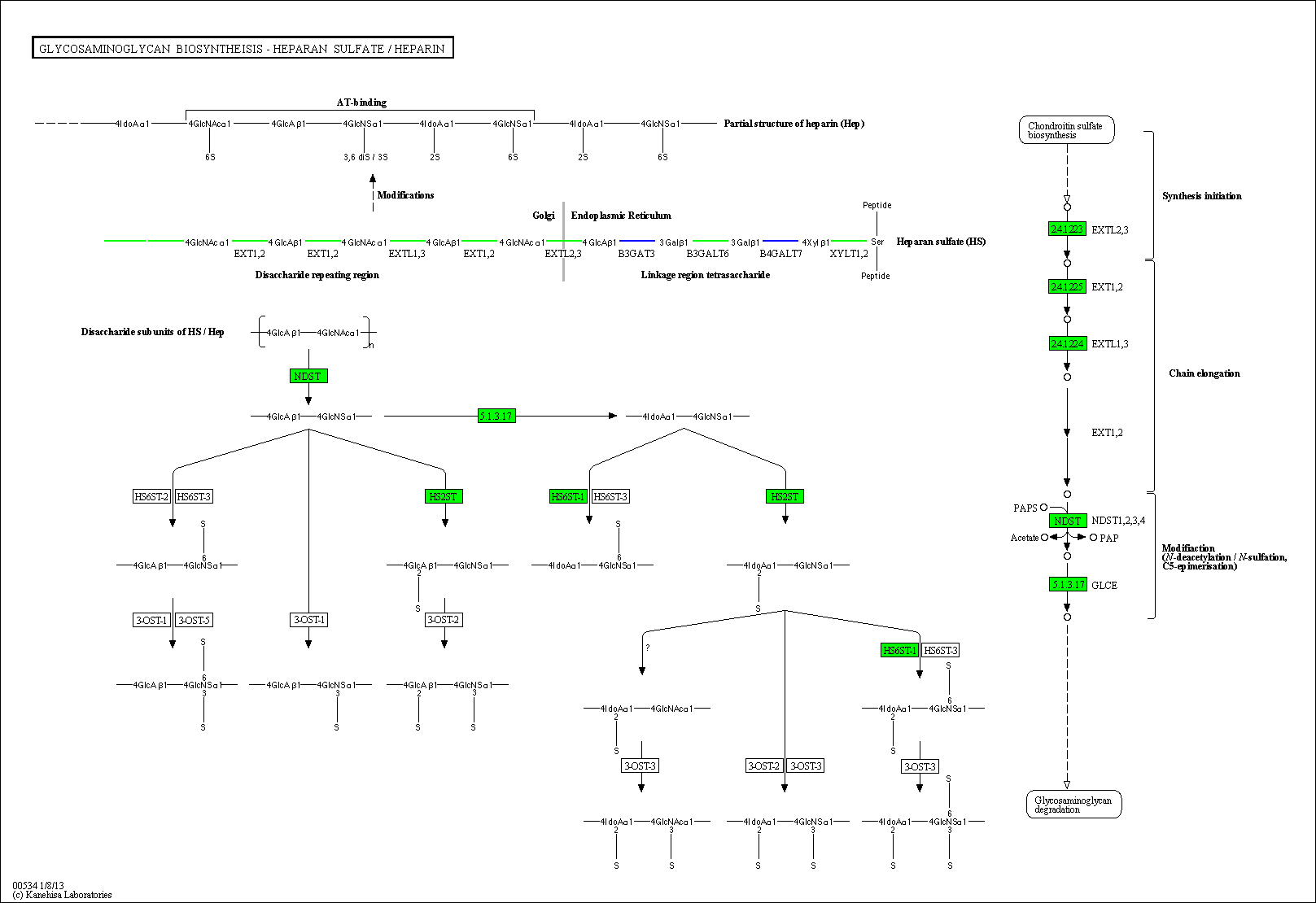


**H8 Glycosylphosphatidylinositol-anchor** **biosynthesis:**

The two free-living species are capable of glycosylphosphatidylinositol anchor biosynthesis.


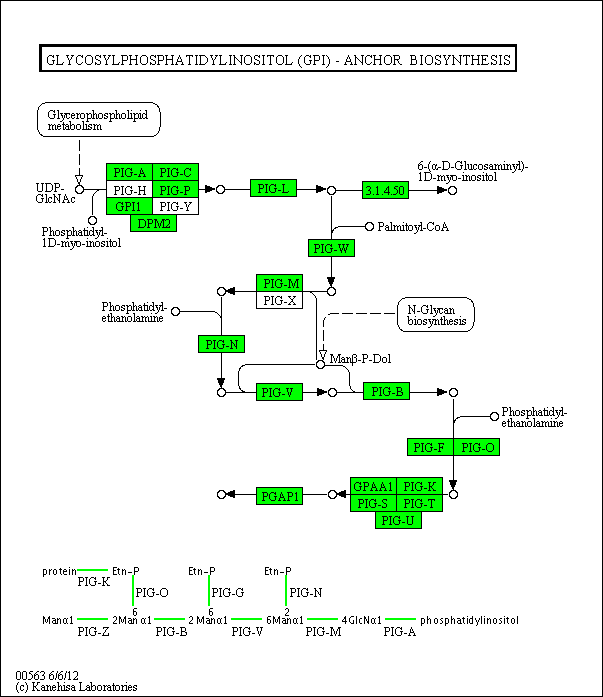


**H9 Glycosphingolipid biosynthesis—lacto and neolacto series:**

*T. kitauei* and *N. vectensis* are capable of glycosphingolipid biosynthesis. *T. kitauei* has enzymes that convert lactosylceramide to Lc3Cer (first step), then to paragloboside (first step of the neolacto series). *N. vectensis* and *H. magnipapillata* have enzymes for converting forssman to globoside, then to Pk antigen.


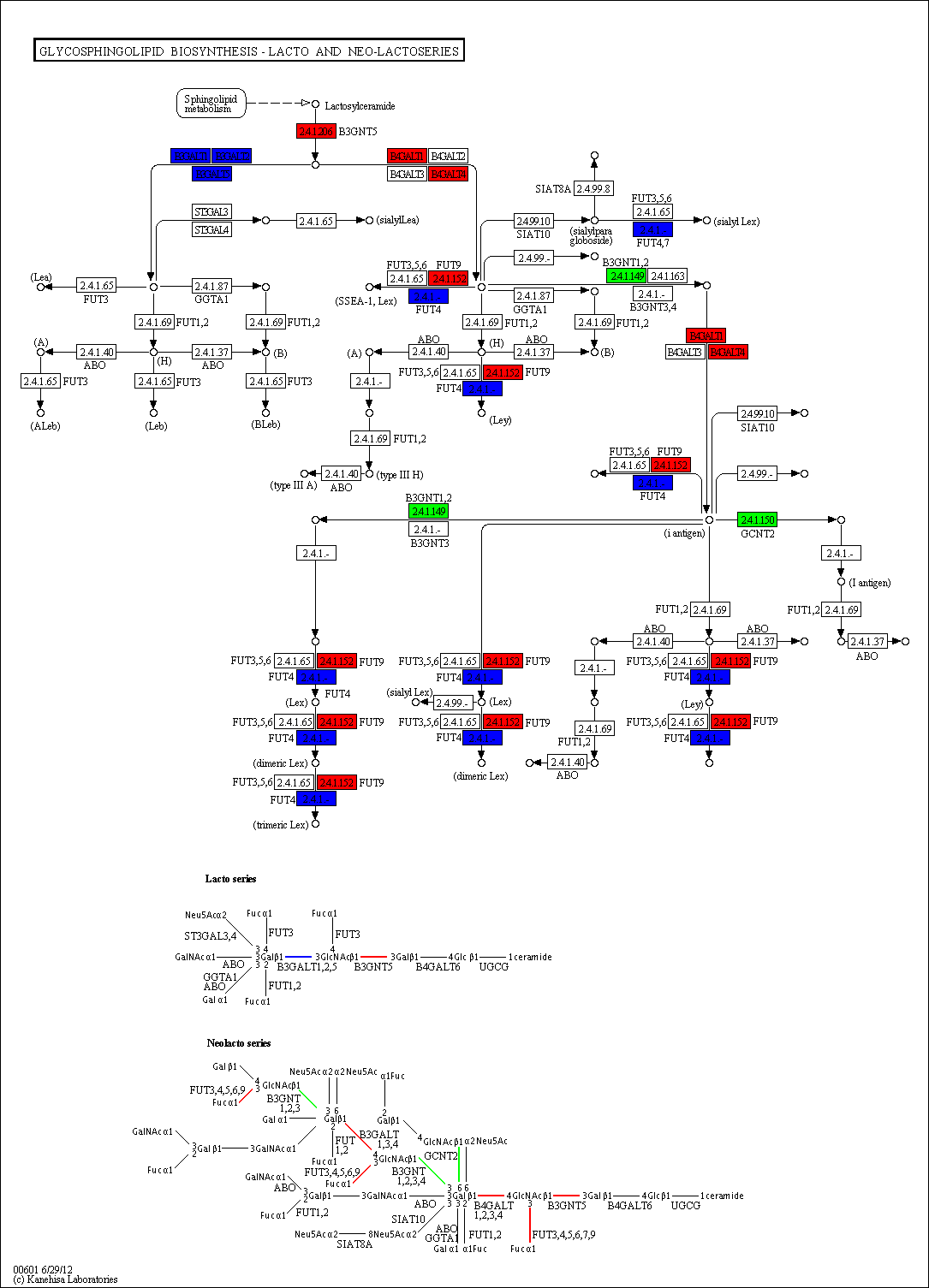


**H10 Glycosphingolipid biosynthesis—globoseries:**

*T. kitauei* can convert Pk antigen to globoside (P antigen) to (SSEA-3). *N. vectensis* and *H. magnipapillata* have enzymes to convert forssman to globoside, then to Pk antigen.


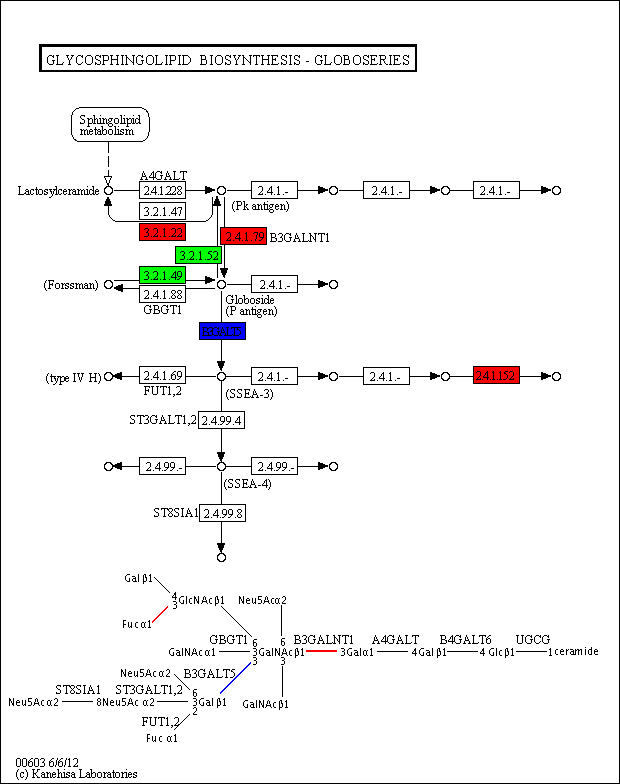


***I Metabolism of Other Amino Acids***

**I1 β-alanine metabolism:**

All three species can form β-alanine from carnosine and β-aminopropionaldehyde. The two free-living species form β-alanine from uracil.


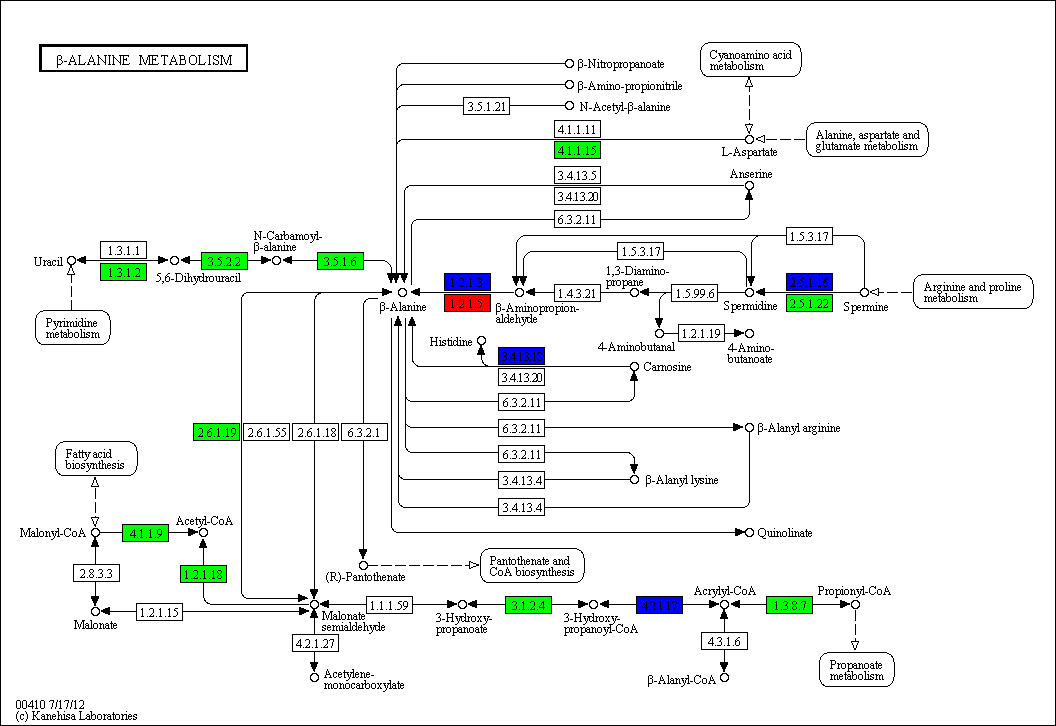


**I2 S****elenocysteine metabolism:**

All three species can form selenomethionine from selenohomocysteine. The two free-living species can generate selenocysteine from alanine and hydrogen selenide. *T. kitauei* has the enzyme cystathionine γ-synthase, which catalyzes O4-succinyl-L-homoserine and L-cysteine to form L-cystathionine and succinate


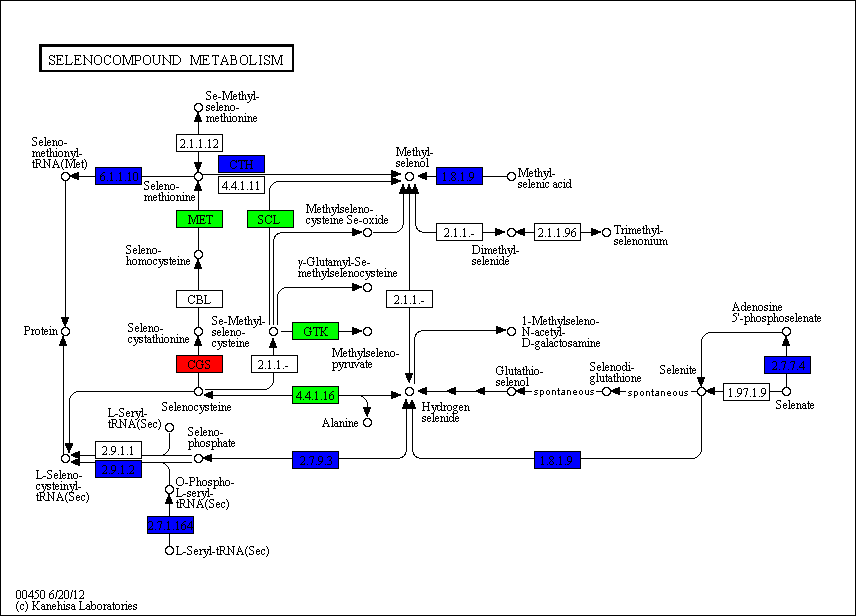


**I3 Glutathione metabolism:**

All three species have the same pathway for glutathione synthesis and utilization via the oxidation/reduction cycle.


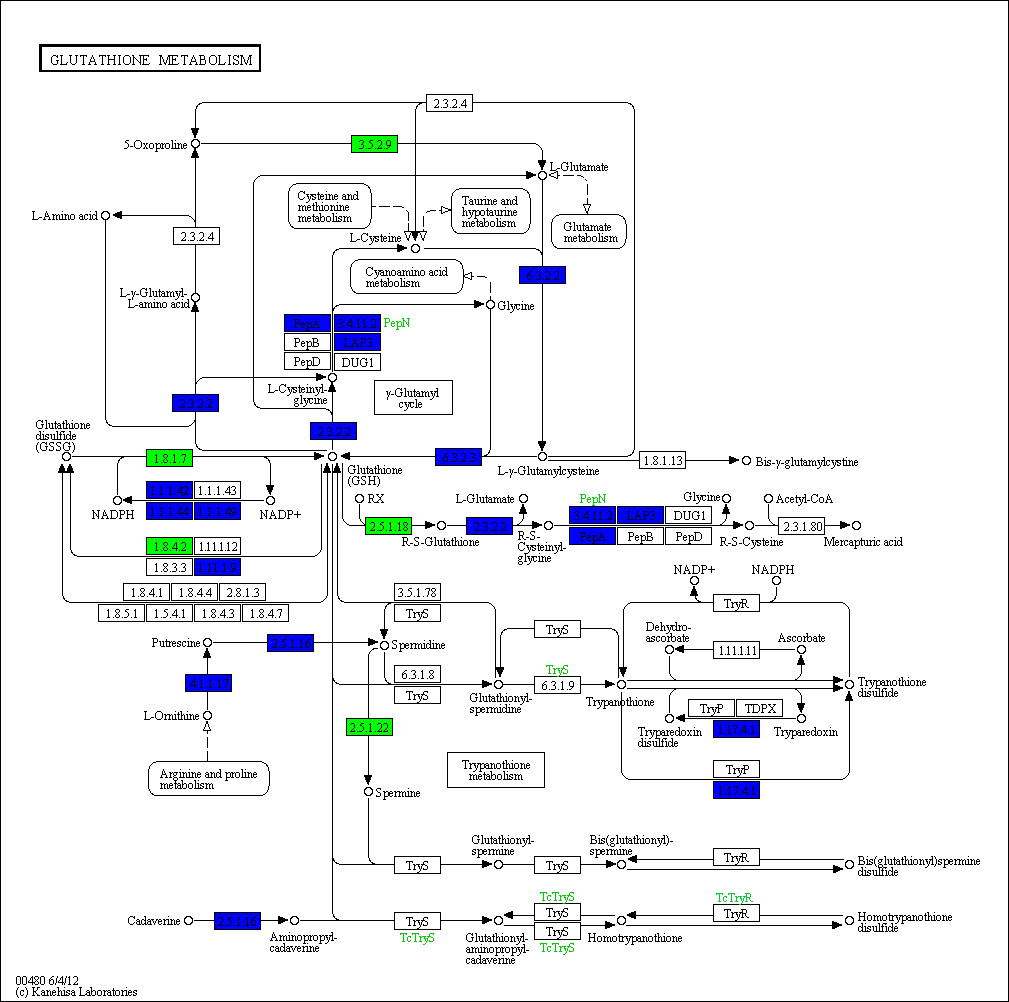


***J Metabolism of Terpenoids and Polyketides***

**J1 Polyketide sugar unit biosynthesis:**

*T. kitauei* has enzymes of the dTDP-l-rhamnose biosynthesis pathway. dTDP-l-rhamnose is a key intermediate in many pathogenic bacteria because it is the donor for l-rhamnose, which is found in the cell wall of many human pathogens such as *Mycobacteria tuberculosis* and *Salmonella typhimurium*.


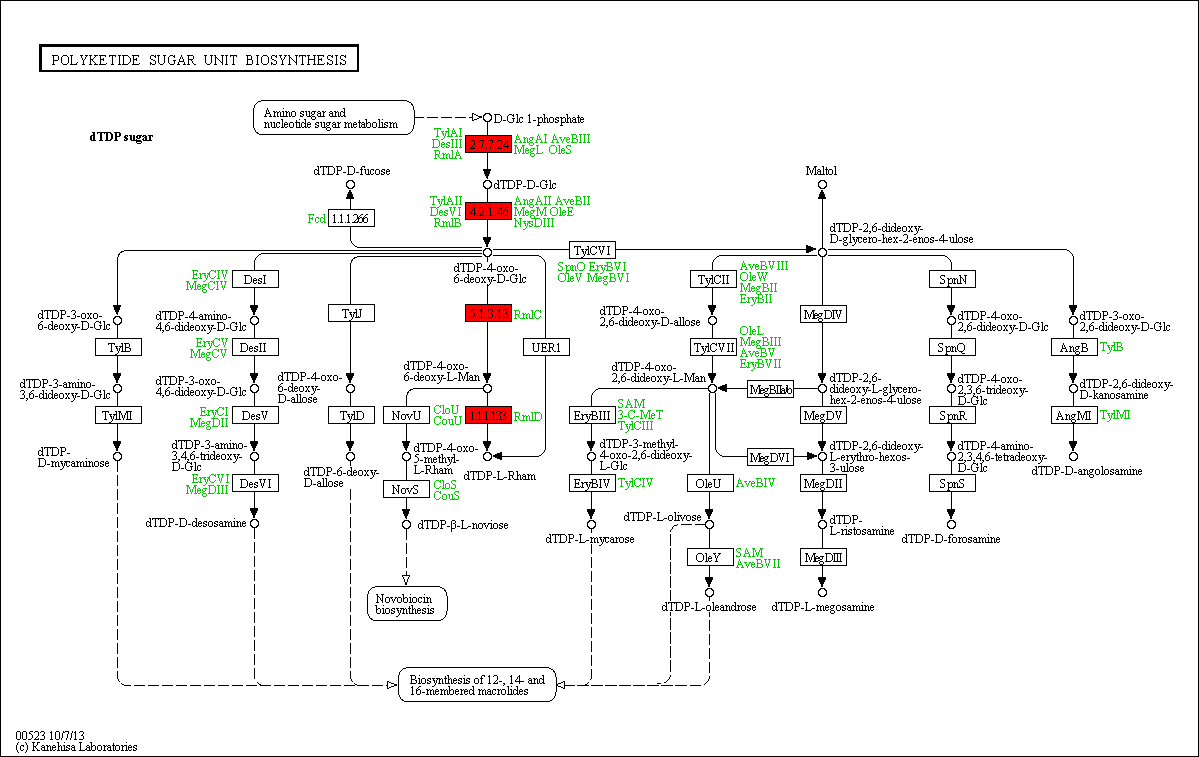


**J2 Terpenoid backbone biosynthesis:**

All three species make the terpenoid backbone using the mevalonate pathway.


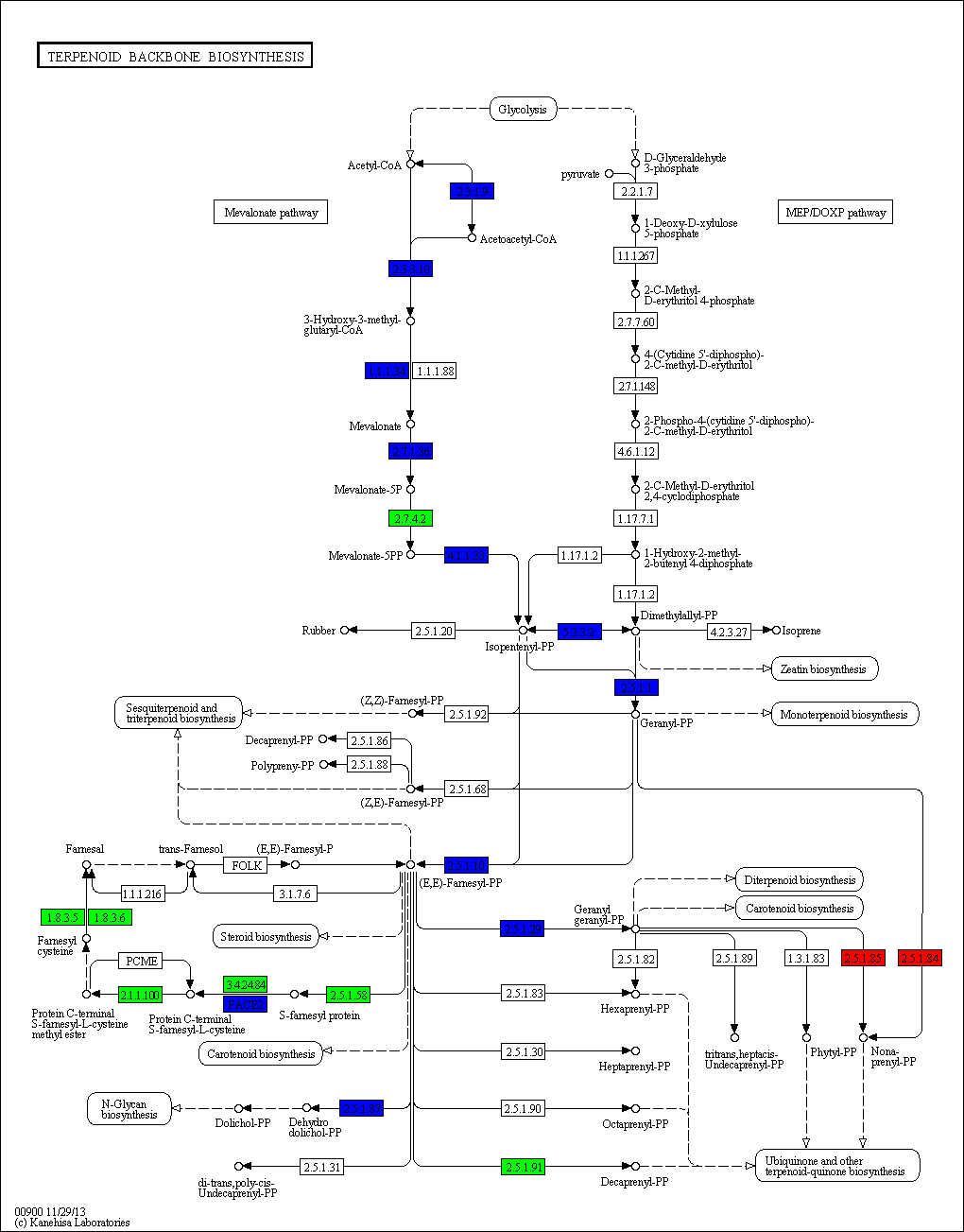


***K Xenobiotics Biodegradation and Metabolism***

**K1 Metabolism of xenobiotics by cytochrome P450:**

The two free-living species are capable of metabolizing xenobiotics by cytochrome P450.


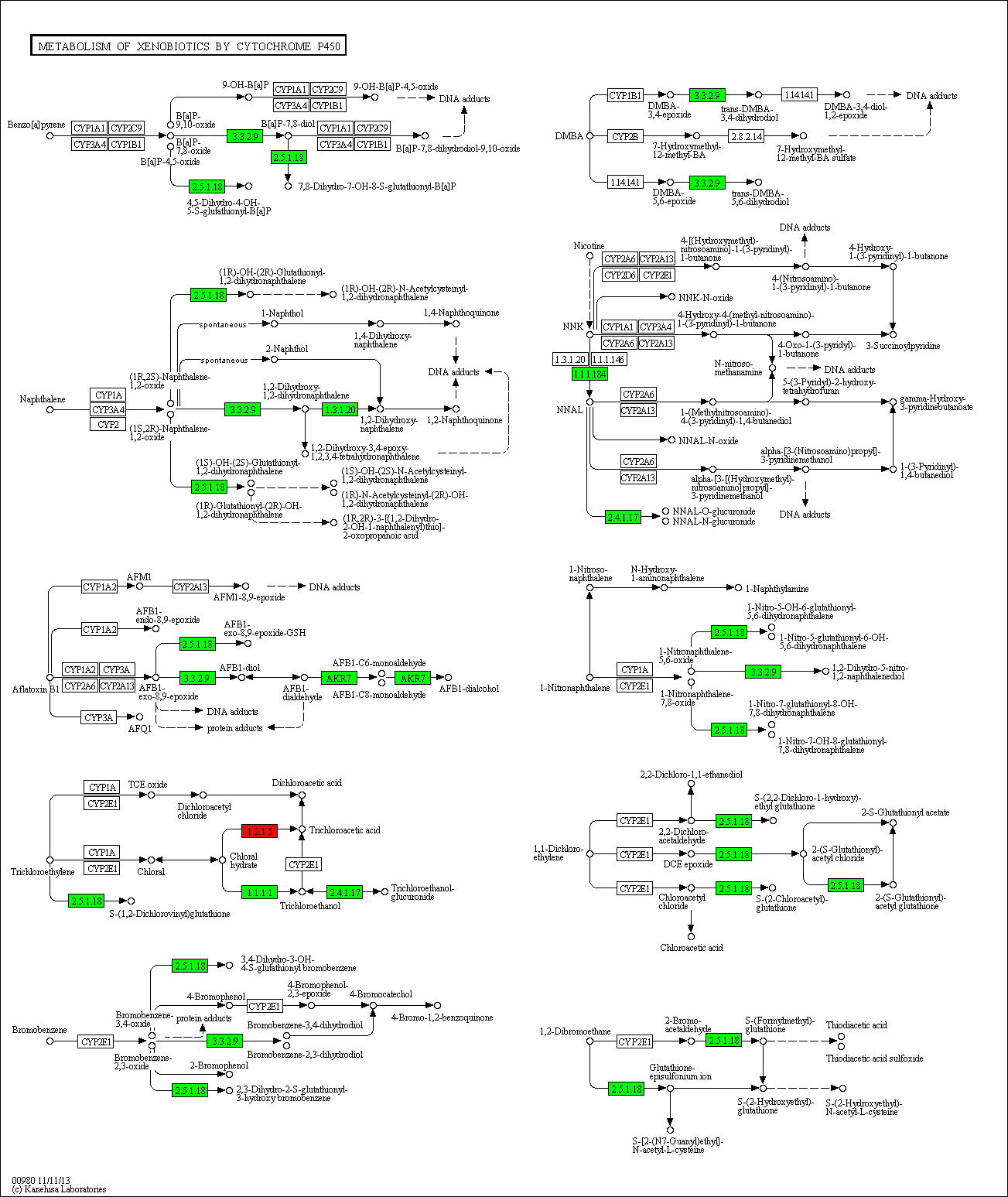


**K2 Drug metabolism—other enzymes:**

The two free-living species are capable of drug metabolism.


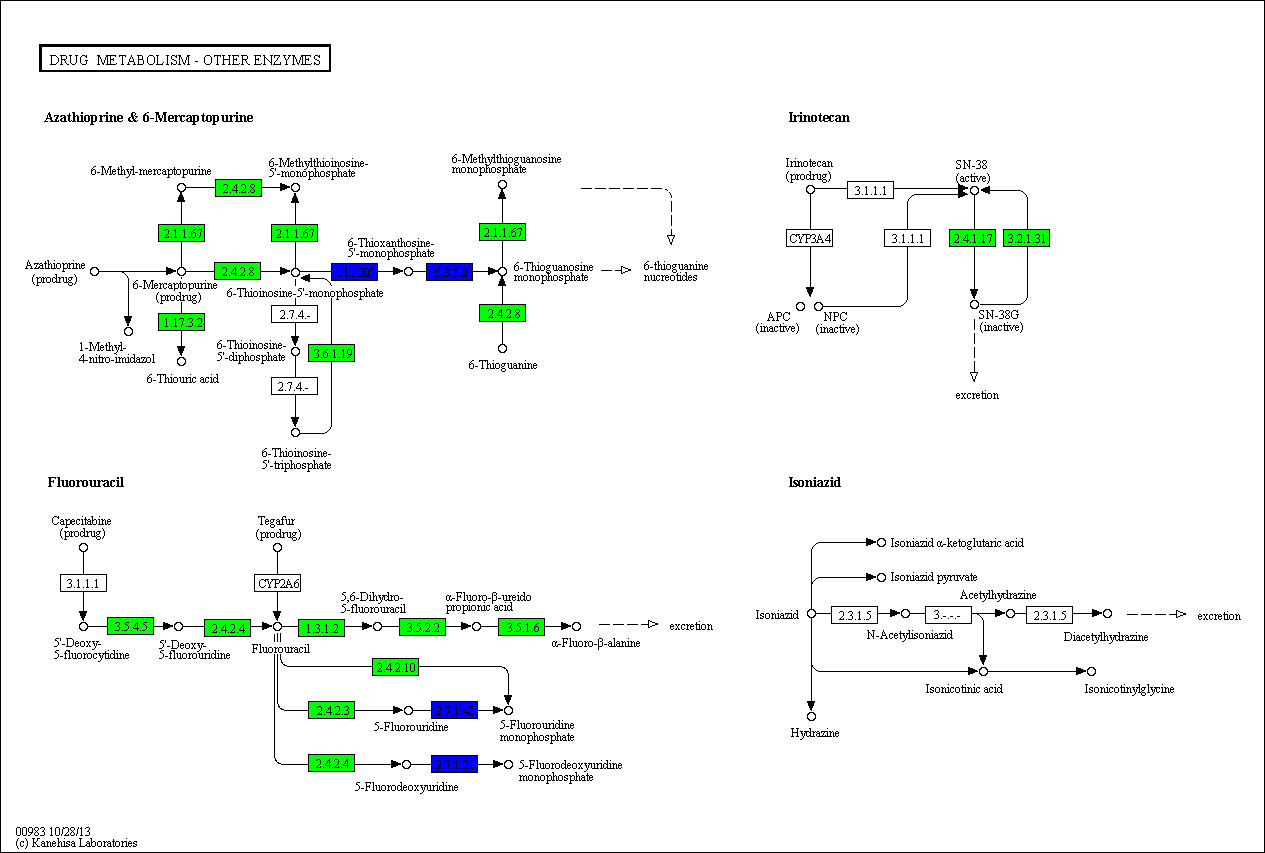

Supplement: Supplementary Data [file supp_evu247_Figures_S1-S5.docx]
