# Supplementary material for: The Genome of the Myxosporean Thelohanellus kitauei Shows Adaptations to Nutrient Acquisition within Its Fish Host
Source: Genome Biol Evol. 2014 Nov 8;6(12):3182–98. doi: 10.1093/gbe/evu247 (PMC4986447; doi:10.1093/gbe/evu247)
Supplement: Supplementary Data [file supp_evu247_Tables_S1-S15.docx]

**Table S1** Morphometric parameters of *T. kitauei* spores

| Parasite |  |  |  |  |  |
| --- | --- | --- | --- | --- | --- |
| Source | This study | Liu et al. 2011 | Xie et al. 2000 | Egusa and Nakajima 1981 | Zhou et al. 1998 |
| Spore length | 25.9 ± 1.0 (24.0–27.8) | 25.5 ± 1.1 (23.4–27.5) | 25.56 (23.0–26.5) | 26.3 (23–29) | 27.1 (25.9–28.4) |
| Spore width | 10.9 ± 1.2 (8.9–13.3) | 8.9 ± 0.8 (7.9–10.9) | 9.05 (8.0–10.0) | 9.2 (8–11) | 10.3 (9.4–12.5) |
| Spore thickness | 8.0 ± 0.3 (7.2–8.5) | 9.3 ± 0.5 (8.3–10.6) | - | - | - |
| Polar capsule length | 14.8 ± 0.9 (13.0–16.2) | 15.0 ± 1.2 (13.0–18.0) | 14.65 (11.2–16.0) | 16.8 (14–18) | 16.8 (15.5–18.1) |
| Polar capsule width | 7.2 ± 0.6 (6.4–8.8) | 7.3 ± 0.5 (6.3–8.5) | 7.22 (6.2–8.2) | 7.4 (6–9) | 8.0 (7.5–8.8) |
| Membrane sheath length | 36.0 ± 2.2 (31.2–40.5) | 34.0 ± 2.6 (30–40.5) | 33.54 (28.0–34.5) | 33.4 (31–35) | 32.8 (31.1–33.6) |
| Membrane sheath width | 17.1 ± 2.0 (14.8–22.0) | 14.5 ± 1.1 (12.2–17.0) | 13.71 (11.2–16.2) | 15.0 (12–17) | 13.6 (11.8–14.6) |

All measurements are in μm; mean ± standard deviation (if available) and range in parentheses.

**Table S2** Identity of 18S rRNA sequence of the parasite from commom carp in the present study to other *Thelohanellus* spp. 18S rRNA sequences available in GenBank

| Species | DNA sequence identity (%) to parasite from common carp in present study | GenBank No. |
| --- | --- | --- |
| *T. kitauei*-China | 995/1005 (99%) | HQ115585 |
| *T. kitauei*-Korea | 1437/1449 (99%) | HM624024 |
| *T. hovorkai*-Hungary | 1064/1113 (96%) | DQ231155 |
| *T. nikolskii*-Hungary | 990/1144 (87%) | DQ231156 |
| *T. nikolskii*-China | 1281/1444 (89%) | GU165832 |
| *T. sinensis*-China | 646/722 (89%) | DQ452013 |
| *T. wuhanensis*-China | 1359/1452 (94%) | HQ613410 |
| *T. wuhanensis*-China2 | 631/683 (92%) | AY165181 |
| *T. zahrahae*-Malaysia | 998/1164 (86%) | EU643622 |
| *T. toyamai-*USA | 639/723 (88%) | HQ338729.1 |

**Table S3** Interspersed repeat sequences in the *T. Kitauei* genome

| **Type of repeated sequence** | **Number of elements** | **Length occupied (bp)** | **Percentage of sequence** |
| --- | --- | --- | --- |
| SINE | 0 | 0 | 0.00 |
| LINE | 85 | 109,305 | 0.07 |
| LTR elements | 1021 | 976,534 | 0.65 |
| DNA elements | 22,963 | 10,478,724 | 6.96 |
| Unclassified | 29,304 | 9,264,231 | 6.15 |
| Total | 53,373 | 20,828,794 | 13.83 |

LINE: long interspersed nuclear element; LTR: long terminal repeat.

**Table S4** Classification of *T. kitauei* transposons

| **Pfam Protein family** | **Pfam ID** | **Gene number** | **Description** |
| --- | --- | --- | --- |
| rve | PF00665.21 | 290 | Integrase that mediates integration of a DNA copy |
| DDE_3 | PF13358.1 | 264 | Endonucleases of the DDE superfamily |
| RVT_1 | PF00078.22 | 249 | Reverse transcriptase |
| DDE_Tnp_IS1595 | PF12762.2 | 179 | Integrase |
| RVP | PF00077.15 | 129 | Aspartyl protease |
| DDE_1 | PF03184.14 | 52 | Endonucleases of the DDE superfamily |
| MULE | PF10551.4 | 49 | MULE transposase |
| Retrotrans_gag | PF03732.12 | 12 | Gag or capsid-like proteins from LTR retrotransposons |

Protein family assigned by the Pfam database, and only the families with more than 10 members are showed in here.

**Table S5** The signaling pathways present in multicellular Metazoa but not in fungi or choanoflagellates

| **Gene_ID** | **RPKM (myxospore stage)** | **KEGG ortholog (KO) number** | **KEGG pathway** |
| --- | --- | --- | --- |
| evm.model.scaffold04230.1 | 57.80 | K03083 | Hedgehog signaling pathway KEGG PATHWAY ko04340 |
| scaffold03844-g609.t1 | 0.00 | K06233 | Hedgehog signaling pathway KEGG PATHWAY ko04340 |
| evm.model.scaffold02630.26 | 0.00 | K06233 | Hedgehog signaling pathway KEGG PATHWAY ko04340 |
| evm.model.scaffold05371.6 | 0.00 | K06233 | Hedgehog signaling pathway KEGG PATHWAY ko04340 |
| evm.model.scaffold03895.9 | 27.82 | K03083 | Hedgehog signaling pathway KEGG PATHWAY ko04340 |
| evm.model.scaffold03257.1 | 0.00 | K06233 | Hedgehog signaling pathway KEGG PATHWAY ko04340 |
| evm.model.scaffold02484.2 | 22.91 | K03362 | Hedgehog signaling pathway KEGG PATHWAY ko04340 |
| evm.model.scaffold02577.74 | 2.83 | K03083 | Hedgehog signaling pathway KEGG PATHWAY ko04340 |
| 13915_t | 0.00 | K06233 | Hedgehog signaling pathway KEGG PATHWAY ko04340 |
| evm.model.scaffold00727.9 | 1.80 | K06233 | Hedgehog signaling pathway KEGG PATHWAY ko04340 |
| evm.model.scaffold03954.12 | 0.27 | K06233 | Hedgehog signaling pathway KEGG PATHWAY ko04340 |
| scaffold00786-g22.t1 | 0.00 | K06233 | Hedgehog signaling pathway KEGG PATHWAY ko04340 |
| evm.model.scaffold00661.16 | 0.00 | K06233 | Hedgehog signaling pathway KEGG PATHWAY ko04340 |
| evm.model.scaffold04180.10 | 6.25 | K06233 | Hedgehog signaling pathway KEGG PATHWAY ko04340 |
| evm.model.scaffold02167.3 | 0.00 | K06233 | Hedgehog signaling pathway KEGG PATHWAY ko04340 |
| evm.model.scaffold04704.6 | 59.27 | K03362 | Hedgehog signaling pathway KEGG PATHWAY ko04340 |
| evm.model.scaffold01473.36 | 0.08 | K06233 | Hedgehog signaling pathway KEGG PATHWAY ko04340 |
| scaffold02256-g661.t1 | 0.00 | K06233 | Hedgehog signaling pathway KEGG PATHWAY ko04340 |
| evm.model.scaffold02577.73 | 1.08 | K06233 | Hedgehog signaling pathway KEGG PATHWAY ko04340 |
| evm.model.scaffold01980.5 | 58.34 | K08958 | Hedgehog signaling pathway KEGG PATHWAY ko04340 |
| evm.model.scaffold05473.12 | 144.54 | K06233 | Hedgehog signaling pathway KEGG PATHWAY ko04340 |
| 19607_t | 0.00 | K06233 | Hedgehog signaling pathway KEGG PATHWAY ko04340 |
| evm.model.scaffold02750.44 | 6.07 | K06233 | Hedgehog signaling pathway KEGG PATHWAY ko04340 |
| evm.model.scaffold05633.24 | 72.58 | K06233 | Hedgehog signaling pathway KEGG PATHWAY ko04340 |
| evm.model.scaffold01396.1 | 0.00 | K06233 | Hedgehog signaling pathway KEGG PATHWAY ko04340 |
| evm.model.scaffold02793.15 | 0.00 | K06233 | Hedgehog signaling pathway KEGG PATHWAY ko04340 |
| evm.model.scaffold04473.10 | 396.67 | K02218 | Hedgehog signaling pathway KEGG PATHWAY ko04340 |
| evm.model.scaffold01358.1 | 87.38 | K04705 | Jak-STAT signaling pathway KEGG PATHWAY ko04630 |
| scaffold04913-g704.t1 | 0.00 | K07293 | Jak-STAT signaling pathway KEGG PATHWAY ko04630 |
| evm.model.scaffold02689.1 | 18.40 | K04498 | Jak-STAT signaling pathway KEGG PATHWAY ko04630 |
| evm.model.scaffold04423.48 | 11.72 | K00922 | Jak-STAT signaling pathway KEGG PATHWAY ko04630 |
| evm.model.scaffold04913.75 | 1.22 | K07293 | Jak-STAT signaling pathway KEGG PATHWAY ko04630 |
| scaffold04913-g675.t1 | 4.42 | K07293 | Jak-STAT signaling pathway KEGG PATHWAY ko04630 |
| scaffold00041-g413.t1 | 0.00 | K04706 | Jak-STAT signaling pathway KEGG PATHWAY ko04630 |
| evm.model.scaffold04423.50 | 2.69 | K00922 | Jak-STAT signaling pathway KEGG PATHWAY ko04630 |
| scaffold05380-g15.t1 | 2.59 | K04456 | Jak-STAT signaling pathway KEGG PATHWAY ko04630 |
| evm.model.scaffold04490.1 | 7.47 | K04456 | Jak-STAT signaling pathway KEGG PATHWAY ko04630 |
| evm.model.scaffold04165.3 | 5.23 | K04706 | Jak-STAT signaling pathway KEGG PATHWAY ko04630 |
| evm.model.scaffold04162.57 | 17.66 | K04497 | Notch signaling pathway KEGG PATHWAY ko04330 |
| evm.model.scaffold03528.1 | 14.74 | K04522 | Notch signaling pathway KEGG PATHWAY ko04330 |
| evm.model.scaffold03856.22 | 1.55 | K06059 | Notch signaling pathway KEGG PATHWAY ko04330 |
| evm.model.scaffold02689.1 | 18.40 | K04498 | Notch signaling pathway KEGG PATHWAY ko04330 |
| evm.model.scaffold00164.3 | 33.63 | K06171 | Notch signaling pathway KEGG PATHWAY ko04330 |
| evm.model.scaffold02750.56 | 1542.25 | K06170 | Notch signaling pathway KEGG PATHWAY ko04330 |
| 17320_t | 0.00 | K02599 | Notch signaling pathway KEGG PATHWAY ko04330 |
| evm.model.scaffold00485.11 | 102.46 | K06063 | Notch signaling pathway KEGG PATHWAY ko04330 |
| evm.model.scaffold01690.6 | 1.21 | K02599 | Notch signaling pathway KEGG PATHWAY ko04330 |
| evm.model.scaffold01045.57 | 0.91 | K06059 | Notch signaling pathway KEGG PATHWAY ko04330 |
| 1963_t | 0.00 | K06053 | Notch signaling pathway KEGG PATHWAY ko04330 |
| evm.model.scaffold00485.12 | 42.67 | K06063 | Notch signaling pathway KEGG PATHWAY ko04330 |
| evm.model.scaffold00766.62 | 116.64 | K06067 | Notch signaling pathway KEGG PATHWAY ko04330 |
| evm.model.scaffold00726.24 | 0.00 | K04658 | TGF-beta signaling pathway KEGG PATHWAY ko04350 |
| evm.model.scaffold05190.4 | 5.61 | K04667 | TGF-beta signaling pathway KEGG PATHWAY ko04350 |
| evm.model.scaffold02630.8 | 0.05 | K04501 | TGF-beta signaling pathway KEGG PATHWAY ko04350 |
| evm.model.scaffold02649.4 | 55.42 | K04682 | TGF-beta signaling pathway KEGG PATHWAY ko04350 |
| evm.model.scaffold05267.17 | 0.07 | K04676 | TGF-beta signaling pathway KEGG PATHWAY ko04350 |
| evm.model.scaffold04162.41 | 0.29 | K04676 | TGF-beta signaling pathway KEGG PATHWAY ko04350 |
| evm.model.scaffold02786.6 | 0.00 | K04676 | TGF-beta signaling pathway KEGG PATHWAY ko04350 |
| evm.model.scaffold02689.1 | 18.40 | K04498 | TGF-beta signaling pathway KEGG PATHWAY ko04350 |
| evm.model.scaffold01295.22 | 32.56 | K04371 | TGF-beta signaling pathway KEGG PATHWAY ko04350 |
| evm.model.scaffold02580.2 | 1608.73 | K04513 | TGF-beta signaling pathway KEGG PATHWAY ko04350 |
| evm.model.scaffold01828.18 | 0.11 | K04681 | TGF-beta signaling pathway KEGG PATHWAY ko04350 |
| evm.model.scaffold00726.17 | 0.10 | K04658 | TGF-beta signaling pathway KEGG PATHWAY ko04350 |
| evm.model.scaffold00644.5 | 24.18 | K04514 | TGF-beta signaling pathway KEGG PATHWAY ko04350 |
| evm.model.scaffold01674.9 | 6.64 | K03347 | TGF-beta signaling pathway KEGG PATHWAY ko04350 |
| evm.model.scaffold01004.2 | 17.39 | K04514 | TGF-beta signaling pathway KEGG PATHWAY ko04350 |
| evm.model.scaffold05190.15 | 71.44 | K04382 | TGF-beta signaling pathway KEGG PATHWAY ko04350 |
| evm.model.scaffold03590.10 | 0.00 | K08268 | mTOR signaling pathway KEGG PATHWAY ko04150 |
| evm.model.scaffold03460.19 | 2.65 | K08267 | mTOR signaling pathway KEGG PATHWAY ko04150 |
| evm.model.scaffold04423.48 | 11.72 | K00922 | mTOR signaling pathway KEGG PATHWAY ko04150 |
| evm.model.scaffold00152.12 | 0.04 | K04373 | mTOR signaling pathway KEGG PATHWAY ko04150 |
| evm.model.scaffold00100.3 | 2.85 | K07203 | mTOR signaling pathway KEGG PATHWAY ko04150 |
| evm.model.scaffold05533.7 | 0.00 | K08268 | mTOR signaling pathway KEGG PATHWAY ko04150 |
| scaffold03463-g595.t1 | 4.15 | K08266 | mTOR signaling pathway KEGG PATHWAY ko04150 |
| evm.model.scaffold04423.50 | 2.69 | K00922 | mTOR signaling pathway KEGG PATHWAY ko04150 |
| scaffold05380-g15.t1 | 2.59 | K04456 | mTOR signaling pathway KEGG PATHWAY ko04150 |
| evm.model.scaffold02899.37 | 14.13 | K02991 | mTOR signaling pathway KEGG PATHWAY ko04150 |
| evm.model.scaffold04490.1 | 7.47 | K04456 | mTOR signaling pathway KEGG PATHWAY ko04150 |
| evm.model.scaffold01295.22 | 32.56 | K04371 | mTOR signaling pathway KEGG PATHWAY ko04150 |
| evm.model.scaffold03128.1 | 176.40 | K02991 | mTOR signaling pathway KEGG PATHWAY ko04150 |
| evm.model.scaffold03372.1 | 7.82 | K06276 | mTOR signaling pathway KEGG PATHWAY ko04150 |
| evm.model.scaffold05380.2 | 18.82 | K03259 | mTOR signaling pathway KEGG PATHWAY ko04150 |
| evm.model.scaffold02448.22 | 35.86 | K04365 | mTOR signaling pathway KEGG PATHWAY ko04150 |
| evm.model.scaffold00795.1 | 50.74 | K03259 | mTOR signaling pathway KEGG PATHWAY ko04150 |
| 17797_t | 0.22 | K02991 | mTOR signaling pathway KEGG PATHWAY ko04150 |
| evm.model.scaffold00761.28 | 0.73 | K08271 | mTOR signaling pathway KEGG PATHWAY ko04150 |
| evm.model.scaffold04204.1 | 16.96 | K03259 | mTOR signaling pathway KEGG PATHWAY ko04150 |
| evm.model.scaffold01629.16 | 84.06 | K08272 | mTOR signaling pathway KEGG PATHWAY ko04150 |
| evm.model.scaffold03372.2 | 6.84 | K06276 | mTOR signaling pathway KEGG PATHWAY ko04150 |
| evm.model.scaffold04230.1 | 57.80 | K03083 | Wnt signaling pathway KEGG PATHWAY ko04310 |
| evm.model.scaffold04623.102 | 49.68 | K03097 | Wnt signaling pathway KEGG PATHWAY ko04310 |
| evm.model.scaffold05497.21 | 301.74 | K06268 | Wnt signaling pathway KEGG PATHWAY ko04310 |
| evm.model.scaffold03460.35 | 32.86 | K03115 | Wnt signaling pathway KEGG PATHWAY ko04310 |
| 824_t | 51.91 | K11584 | Wnt signaling pathway KEGG PATHWAY ko04310 |
| evm.model.scaffold04162.57 | 17.66 | K04497 | Wnt signaling pathway KEGG PATHWAY ko04310 |
| evm.model.scaffold03895.9 | 27.82 | K03083 | Wnt signaling pathway KEGG PATHWAY ko04310 |
| evm.model.scaffold02630.8 | 0.05 | K04501 | Wnt signaling pathway KEGG PATHWAY ko04310 |
| evm.model.scaffold04712.6 | 258.58 | K03068 | Wnt signaling pathway KEGG PATHWAY ko04310 |
| evm.model.scaffold03549.1 | 176.17 | K04515 | Wnt signaling pathway KEGG PATHWAY ko04310 |
| evm.model.scaffold02712.3 | 20.81 | K04494 | Wnt signaling pathway KEGG PATHWAY ko04310 |
| evm.model.scaffold02484.2 | 22.91 | K03362 | Wnt signaling pathway KEGG PATHWAY ko04310 |
| evm.model.scaffold05175.5 | 0.00 | K03068 | Wnt signaling pathway KEGG PATHWAY ko04310 |
| evm.model.scaffold02032.1 | 0.32 | K03115 | Wnt signaling pathway KEGG PATHWAY ko04310 |
| evm.model.scaffold02577.74 | 2.83 | K03083 | Wnt signaling pathway KEGG PATHWAY ko04310 |
| evm.model.scaffold02689.1 | 18.40 | K04498 | Wnt signaling pathway KEGG PATHWAY ko04310 |
| evm.model.scaffold03460.36 | 1.19 | K03115 | Wnt signaling pathway KEGG PATHWAY ko04310 |
| evm.model.scaffold04311.4 | 0.00 | K03068 | Wnt signaling pathway KEGG PATHWAY ko04310 |
| evm.model.scaffold03076.3 | 6.11 | K03068 | Wnt signaling pathway KEGG PATHWAY ko04310 |
| evm.model.scaffold05175.4 | 0.00 | K03068 | Wnt signaling pathway KEGG PATHWAY ko04310 |
| evm.model.scaffold03454.25 | 111.91 | K03068 | Wnt signaling pathway KEGG PATHWAY ko04310 |
| evm.model.scaffold00691.20 | 0.09 | K02105 | Wnt signaling pathway KEGG PATHWAY ko04310 |
| evm.model.scaffold02512.1 | 147.93 | K04348 | Wnt signaling pathway KEGG PATHWAY ko04310 |
| evm.model.scaffold02750.81 | 35.51 | K04499 | Wnt signaling pathway KEGG PATHWAY ko04310 |
| evm.model.scaffold04423.61 | 1150.89 | K04507 | Wnt signaling pathway KEGG PATHWAY ko04310 |
| evm.model.scaffold04704.6 | 59.27 | K03362 | Wnt signaling pathway KEGG PATHWAY ko04310 |
| evm.model.scaffold02580.2 | 1608.73 | K04513 | Wnt signaling pathway KEGG PATHWAY ko04310 |
| evm.model.scaffold05565.19 | 0.40 | K11584 | Wnt signaling pathway KEGG PATHWAY ko04310 |
| evm.model.scaffold02750.61 | 66.17 | K04348 | Wnt signaling pathway KEGG PATHWAY ko04310 |
| evm.model.scaffold03033.6 | 10.84 | K05858 | Wnt signaling pathway KEGG PATHWAY ko04310 |
| evm.model.scaffold00644.5 | 24.18 | K04514 | Wnt signaling pathway KEGG PATHWAY ko04310 |
| evm.model.scaffold01674.9 | 6.64 | K03347 | Wnt signaling pathway KEGG PATHWAY ko04310 |
| evm.model.scaffold03983.19 | 6.21 | K03068 | Wnt signaling pathway KEGG PATHWAY ko04310 |
| evm.model.scaffold02302.6 | 64.16 | K04508 | Wnt signaling pathway KEGG PATHWAY ko04310 |
| evm.model.scaffold03580.62 | 70.23 | K03068 | Wnt signaling pathway KEGG PATHWAY ko04310 |
| evm.model.scaffold01004.2 | 17.39 | K04514 | Wnt signaling pathway KEGG PATHWAY ko04310 |
| evm.model.scaffold05190.15 | 71.44 | K04382 | Wnt signaling pathway KEGG PATHWAY ko04310 |
| scaffold04423-g121.t1 | 0.06 | K03068 | Wnt signaling pathway KEGG PATHWAY ko04310 |
| evm.model.scaffold00041.27 | 6.69 | K04491 | Wnt signaling pathway KEGG PATHWAY ko04310 |
| evm.model.scaffold04262.8 | 175.99 | K04506 | Wnt signaling pathway KEGG PATHWAY ko04310 |
| evm.model.scaffold05401.4 | 29.94 | K06268 | Wnt signaling pathway KEGG PATHWAY ko04310 |

**Table S6** Overview of the genomes of obligate parasites and free-living organisms

| **Organism** | **Classification** | **Type** | **Number of predicted proteins** | **Genomic resources** |
| --- | --- | --- | --- | --- |
| *Saccharomyces cerevisiae* | Fungi, Ascomycota | F | 6750 | http://downloads.yeastgenome.org/sequence/S288C_reference/orf_protein/ |
| *Enterocytozoon bieneusi* | Fungi | OP | 3633 | http://microsporidiadb.org/common/downloads/Current_Release/EbieneusiH348/fasta/data/ |
| *Tetrahymena thermophila* | Ciliophora | F | 24,725 | http://www.ciliate.org/system/downloads/T_thermophila_oct2008_proteins.fasta |
| *Trypanosoma brucei* | Euglenozoa | OP | 10,574 | http://tritrypdb.org/common/downloads/Current_Release/TbruceiTREU927/fasta/data/ |
| *Entamoeba histolytica* | Sarcomastigophora | OP | 8113 | ftp://ftp.ensemblgenomes.org/pub/release-20/protists/fasta/entamoeba_histolytica/pep/ |
| *Toxoplasma gondii* | Apicomplexa | OP | 8322 | http://toxodb.org/common/downloads/Current_Release/TgondiiME49/ |
| *Plasmodium falciparum* | Apicomplexa | OP | 5542 | http://plasmodb.org/common/downloads/Current_Release/Pfalciparum3D7/fasta/ |
| *Cryptosporidium hominis* | Apicomplexa | OP | 3934 | http://www.hominis.mic.vcu.edu/g_bulk.html |
| *Hydra magnipapillata* | Cnidaria | F | 17,398 | ftp://ftp.ncbi.nlm.nih.gov/genomes/Hydra_magnipapillata/ |
| *Nematostella vectensis* | Cnidaria | F | 27,273 | ftp://ftp.ensemblgenomes.org/pub/metazoa/release-20/fasta/nematostella_vectensis/pep/ |
| *Thelohanellus kitauei* | Cnidaria | OP | 16,638 | This study http://www.ncbi.nlm.nih.gov/genome/16943 |
| *Echinococcus multilocularis* | Platyhelminthes | OP | 10,780 | ftp://ftp.sanger.ac.uk/pub/pathogens/Echinococcus/multilocularis/genome/ |
| *Schistosoma mansoni* | Platyhelminthes | OP | 11,843 | ftp://ftp.sanger.ac.uk/pub/pathogens/Schistosoma/mansoni/genome/Gene_models/ |

OP, obligate parasite; F, free-living.

**Table S7** Putative proteases in *T. kitauei* and other organisms

| **Family** | **Number of predicted proteases (% of total predicted proteases )** | | | | | |
| --- | --- | --- | --- | --- | --- | --- |
|  | ***T. kitauei*** | ***H. magnipapillata*** | | ***N. vectensis*** | ***S. mansoni*** | ***E. multilocularis*** |
| A01A | 2 (0.5) | | 5 (0.6) | 7 (0.7) | 14 (3.9) | 1 (0.4) |
| A02A | 0 (0.0) | | 1 (0.1) | 1 (0.1) | 1 (0.3) | 0 (0.0) |
| A02D | 1 (0.2) | | 0 (0.0) | 0 (0.0) | 0 (0.0) | 0 (0.0) |
| A08 | 0 (0.0) | | 0 (0.0) | 1 (0.1) | 0 (0.0) | 0 (0.0) |
| A11X | 165 (39.1) | | 25 (3.1) | 0 (0.0) | 7 (2.0) | 0 (0.0) |
| A22A | 1 (0.2) | | 2 (0.2) | 1 (0.1) | 1 (0.3) | 1 (0.4) |
| A22B | 1 (0.2) | | 3 (0.4) | 4 (0.4) | 2 (0.6) | 2 (0.7) |
| A33 | 23 (5.5) | | 1 (0.1) | 2 (0.2) | 0 (0.0) | 1 (0.4) |
| C01A | 25 (5.9) | | 36 (4.5) | 50 (5.1) | 18 (5.0) | 10 (3.7) |
| C01B | 0 (0.0) | | 0 (0.0) | 1 (0.1) | 0 (0.0) | 0 (0.0) |
| C02A | 0 (0.0) | | 6 (0.7) | 8 (0.8) | 9 (2.5) | 7 (2.6) |
| C12 | 0 (0.0) | | 3 (0.4) | 3 (0.3) | 6 (1.7) | 4 (1.5) |
| C13 | 0 (0.0) | | 2 (0.2) | 2 (0.2) | 3 (0.8) | 1 (0.4) |
| C14A | 0 (0.0) | | 24 (3.0) | 9 (0.9) | 2 (0.6) | 5 (1.9) |
| C14B | 0 (0.0) | | 2 (0.2) | 4 (0.4) | 0 (0.0) | 0 (0.0) |
| C15 | 0 (0.0) | | 1 (0.1) | 1 (0.1) | 1 (0.3) | 1 (0.4) |
| C19 | 8 (1.9) | | 42 (5.2) | 49 (5.0) | 22 (6.2) | 25 (9.3) |
| C26 | 3 (0.7) | | 16 (2.0) | 16 (1.6) | 1 (0.3) | 1 (0.4) |
| C44 | 9 (2.1) | | 7 (0.9) | 7 (0.7) | 3 (0.8) | 3 (1.1) |
| C46 | 0 (0.0) | | 4 (0.5) | 7 (0.7) | 0 (0.0) | 1 (0.4) |
| C48 | 3 (0.7) | | 13 (1.6) | 8 (0.8) | 4 (1.1) | 3 (1.1) |
| C50 | 1 (0.2) | | 0 (0.0) | 1 (0.1) | 1 (0.3) | 1 (0.4) |
| C54 | 2 (0.5) | | 3 (0.4) | 2 (0.2) | 2 (0.6) | 2 (0.7) |
| C56 | 1 (0.2) | | 8 (1.0) | 6 (0.6) | 1 (0.3) | 2 (0.7) |
| C59 | 0 (0.0) | | 1 (0.1) | 0 (0.0) | 0 (0.0) | 0 (0.0) |
| C64 | 0 (0.0) | | 4 (0.5) | 4 (0.4) | 1 (0.3) | 1 (0.4) |
| C65 | 0 (0.0) | | 1 (0.1) | 3 (0.3) | 1 (0.3) | 1 (0.4) |
| C67 | 0 (0.0) | | 1 (0.1) | 1 (0.1) | 0 (0.0) | 0 (0.0) |
| C78 | 1 (0.2) | | 4 (0.5) | 2 (0.2) | 1 (0.3) | 2 (0.7) |
| C83 | 0 (0.0) | | 2 (0.2) | 4 (0.4) | 1 (0.3) | 1 (0.4) |
| C85 | 1 (0.2) | | 5 (0.6) | 10 (1.0) | 3 (0.8) | 2 (0.7) |
| C86 | 1 (0.2) | | 2 (0.2) | 3 (0.3) | 2 (0.6) | 2 (0.7) |
| C88 | 0 (0.0) | | 1 (0.1) | 1 (0.1) | 1 (0.3) | 1 (0.4) |
| C89 | 0 (0.0) | | 3 (0.4) | 3 (0.3) | 0 (0.0) | 0 (0.0) |
| M01 | 14 (3.3) | | 14 (1.7) | 18 (1.8) | 5 (1.4) | 9 (3.3) |
| M02 | 0 (0.0) | | 4 (0.5) | 9 (0.9) | 0 (0.0) | 0 (0.0) |
| M03A | 3 (0.7) | | 3 (0.4) | 3 (0.3) | 5 (1.4) | 2 (0.7) |
| M03B | 0 (0.0) | | 0 (0.0) | 1 (0.1) | 0 (0.0) | 0 (0.0) |
| M08 | 0 (0.0) | | 1 (0.1) | 3 (0.3) | 12 (3.4) | 1 (0.4) |
| M10A | 0 (0.0) | | 21 (2.6) | 23 (2.3) | 1 (0.3) | 1 (0.4) |
| M12A | 1 (0.2) | | 68 (8.5) | 88 (8.9) | 2 (0.6) | 2 (0.7) |
| M12B | 5 (1.2) | | 20 (2.5) | 27 (2.7) | 6 (1.7) | 5 (1.9) |
| M13 | 1 (0.2) | | 15 (1.9) | 23 (2.3) | 9 (2.5) | 1 (0.4) |
| M14A | 0 (0.0) | | 7 (0.9) | 9 (0.9) | 1 (0.3) | 1 (0.4) |
| M14B | 6 (1.4) | | 8 (1.0) | 13 (1.3) | 8 (2.2) | 3 (1.1) |
| M14X | 0 (0.0) | | 0 (0.0) | 2 (0.2) | 1 (0.3) | 0 (0.0) |
| M16A | 0 (0.0) | | 4 (0.5) | 3 (0.3) | 6 (1.7) | 4 (1.5) |
| M16B | 3 (0.7) | | 5 (0.6) | 5 (0.5) | 5 (1.4) | 3 (1.1) |
| M16C | 1 (0.2) | | 2 (0.2) | 3 (0.3) | 1 (0.3) | 1 (0.4) |
| M17 | 3 (0.7) | | 7 (0.9) | 4 (0.4) | 2 (0.6) | 4 (1.5) |
| M18 | 1 (0.2) | | 1 (0.1) | 1 (0.1) | 1 (0.3) | 1 (0.4) |
| M19 | 0 (0.0) | | 0 (0.0) | 2 (0.2) | 0 (0.0) | 0 (0.0) |
| M20A | 1 (0.2) | | 3 (0.4) | 3 (0.3) | 1 (0.3) | 3 (1.1) |
| M20D | 0 (0.0) | | 5 (0.6) | 4 (0.4) | 0 (0.0) | 0 (0.0) |
| M20X | 0 (0.0) | | 0 (0.0) | 1 (0.1) | 0 (0.0) | 0 (0.0) |
| M22 | 2 (0.5) | | 3 (0.4) | 4 (0.4) | 5 (1.4) | 4 (1.5) |
| M23B | 11 (2.6) | | 49 (6.1) | 32 (3.2) | 47 (13.2) | 27 (10.0) |
| M24A | 3 (0.7) | | 6 (0.7) | 3 (0.3) | 4 (1.1) | 4 (1.5) |
| M24B | 1 (0.2) | | 6 (0.7) | 10 (1.0) | 4 (1.1) | 4 (1.5) |
| M24X | 1 (0.2) | | 2 (0.2) | 2 (0.2) | 2 (0.6) | 2 (0.7) |
| M28A | 1 (0.2) | | 1 (0.1) | 1 (0.1) | 1 (0.3) | 1 (0.4) |
| M28B | 0 (0.0) | | 5 (0.6) | 19 (1.9) | 1 (0.3) | 1 (0.4) |
| M28X | 0 (0.0) | | 3 (0.4) | 2 (0.2) | 2 (0.6) | 3 (1.1) |
| M38 | 0 (0.0) | | 8 (1.0) | 10 (1.0) | 8 (2.2) | 4 (1.5) |
| M41 | 3 (0.7) | | 9 (1.1) | 3 (0.3) | 3 (0.8) | 3 (1.1) |
| M43A | 0 (0.0) | | 0 (0.0) | 1 (0.1) | 0 (0.0) | 0 (0.0) |
| M43B | 0 (0.0) | | 0 (0.0) | 2 (0.2) | 0 (0.0) | 0 (0.0) |
| M48A | 0 (0.0) | | 1 (0.1) | 2 (0.2) | 1 (0.3) | 1 (0.4) |
| M48X | 0 (0.0) | | 1 (0.1) | 2 (0.2) | 0 (0.0) | 0 (0.0) |
| M49 | 0 (0.0) | | 2 (0.2) | 2 (0.2) | 1 (0.3) | 1 (0.4) |
| M50A | 0 (0.0) | | 1 (0.1) | 1 (0.1) | 1 (0.3) | 1 (0.4) |
| M54 | 0 (0.0) | | 1 (0.1) | 3 (0.3) | 0 (0.0) | 0 (0.0) |
| M67A | 2 (0.5) | | 9 (1.1) | 7 (0.7) | 4 (1.1) | 5 (1.9) |
| M67C | 1 (0.2) | | 2 (0.2) | 1 (0.1) | 1 (0.3) | 1 (0.4) |
| M67X | 2 (0.5) | | 3 (0.4) | 2 (0.2) | 2 (0.6) | 2 (0.7) |
| M76 | 0 (0.0) | | 1 (0.1) | 2 (0.2) | 0 (0.0) | 0 (0.0) |
| S01A | 50 (11.8) | | 41 (5.1) | 132 (13.4) | 17 (4.8) | 6 (2.2) |
| S01B | 0 (0.0) | | 4 (0.5) | 4 (0.4) | 0 (0.0) | 2 (0.7) |
| S01X | 0 (0.0) | | 1 (0.1) | 1 (0.1) | 0 (0.0) | 0 (0.0) |
| S06 | 0 (0.0) | | 0 (0.0) | 1 (0.1) | 0 (0.0) | 0 (0.0) |
| S08A | 0 (0.0) | | 16 (2.0) | 41 (4.2) | 7 (2.0) | 5 (1.9) |
| S08B | 11 (2.6) | | 8 (1.0) | 11 (1.1) | 4 (1.1) | 5 (1.9) |
| S08X | 0 (0.0) | | 0 (0.0) | 1 (0.1) | 0 (0.0) | 0 (0.0) |
| S09A | 0 (0.0) | | 2 (0.2) | 2 (0.2) | 2 (0.6) | 1 (0.4) |
| S09B | 1 (0.2) | | 1 (0.1) | 5 (0.5) | 2 (0.6) | 1 (0.4) |
| S09C | 7 (1.7) | | 4 (0.5) | 3 (0.3) | 1 (0.3) | 1 (0.4) |
| S09X | 7 (1.7) | | 11 (1.4) | 36 (3.7) | 15 (4.2) | 14 (5.2) |
| S10 | 0 (0.0) | | 1 (0.1) | 1 (0.1) | 2 (0.6) | 1 (0.4) |
| S11 | 0 (0.0) | | 1 (0.1) | 0 (0.0) | 0 (0.0) | 0 (0.0) |
| S12 | 0 (0.0) | | 2 (0.2) | 7 (0.7) | 2 (0.6) | 1 (0.4) |
| S14 | 1 (0.2) | | 2 (0.2) | 2 (0.2) | 1 (0.3) | 1 (0.4) |
| S15 | 0 (0.0) | | 0 (0.0) | 5 (0.5) | 0 (0.0) | 0 (0.0) |
| S16 | 2 (0.5) | | 3 (0.4) | 2 (0.2) | 1 (0.3) | 1 (0.4) |
| S24 | 0 (0.0) | | 2 (0.2) | 2 (0.2) | 0 (0.0) | 0 (0.0) |
| S26A | 2 (0.5) | | 3 (0.4) | 4 (0.4) | 1 (0.3) | 1 (0.4) |
| S26B | 1 (0.2) | | 1 (0.1) | 1 (0.1) | 1 (0.3) | 1 (0.4) |
| S28 | 0 (0.0) | | 4 (0.5) | 5 (0.5) | 2 (0.6) | 2 (0.7) |
| S33 | 3 (0.7) | | 12 (1.5) | 20 (2.0) | 6 (1.7) | 7 (2.6) |
| S41A | 1 (0.2) | | 2 (0.2) | 8 (0.8) | 0 (0.0) | 2 (0.7) |
| S41B | 0 (0.0) | | 0 (0.0) | 1 (0.1) | 0 (0.0) | 0 (0.0) |
| S49 | 0 (0.0) | | 0 (0.0) | 2 (0.2) | 0 (0.0) | 0 (0.0) |
| S53 | 0 (0.0) | | 1 (0.1) | 1 (0.1) | 0 (0.0) | 0 (0.0) |
| S54 | 1 (0.2) | | 6 (0.7) | 7 (0.7) | 4 (1.1) | 4 (1.5) |
| S59 | 1 (0.2) | | 1 (0.1) | 1 (0.1) | 1 (0.3) | 1 (0.4) |
| S60 | 0 (0.0) | | 0 (0.0) | 1 (0.1) | 1 (0.3) | 1 (0.4) |
| S63 | 0 (0.0) | | 11 (1.4) | 24 (2.4) | 1 (0.3) | 1 (0.4) |
| S72 | 0 (0.0) | | 1 (0.1) | 3 (0.3) | 0 (0.0) | 0 (0.0) |
| T01A | 13 (3.1) | | 24 (3.0) | 16 (1.6) | 18 (5.0) | 16 (5.9) |
| T02 | 1 (0.2) | | 7 (0.9) | 11 (1.1) | 2 (0.6) | 2 (0.7) |
| T03 | 6 (1.4) | | 80 (10.0) | 20 (2.0) | 4 (1.1) | 2 (0.7) |
| T05 | 0 (0.0) | | 1 (0.1) | 0 (0.0) | 0 (0.0) | 0 (0.0) |
| T06 | 0 (0.0) | | 21 (2.6) | 28 (2.8) | 1 (0.3) | 4 (1.5) |
| Total sum | 422 | | 803 | 968 | 357 | 269 |

**Table** **S8** Proteases and protease inhibitors with predicted signal peptides in the myxospore stage of *T. kitauei*

| **Gene_ID** | **Protease and protease inhibitor subfamily (MEROPS)** | **Expression level*** |
| --- | --- | --- |
| evm.model.scaffold00832.72 | Pepsin (A1A) | 4.45 |
|  |  |  |
| evm.model.scaffold03922.1 | Papain (C1A) | 4977.52 |
| evm.model.scaffold00538.6 | Papain (C1A) | 2408.94 |
| evm.model.scaffold05557.15 | Papain (C1A) | 1621.39 |
| evm.model.scaffold03581.52 | Papain (C1A) | 341.13 |
| evm.model.scaffold03922.2 | Papain (C1A) | 113.45 |
| evm.model.scaffold02902.23 | Papain (C1A) | 22.97 |
| 5496_t | Papain (C1A) | 2.72 |
| evm.model.scaffold04409.9 | Papain (C1A) | 2.25 |
| evm.model.scaffold00766.19 | Papain (C1A) | 2.11 |
| evm.model.scaffold01140.5 | Papain (C1A) | 1.86 |
| evm.model.scaffold01629.14 | Papain (C1A) | 1.22 |
| evm.model.scaffold05371.22 | Papain (C1A) | 0.23 |
| evm.model.scaffold02602.3 | Papain (C1A) | 0.14 |
| evm.model.scaffold00244.2 | Papain (C1A) | 0.07 |
| evm.model.scaffold00661.32 | Papain (C1A) | 0.05 |
| evm.model.scaffold01629.13 | Papain (C1A) | 0.03 |
| evm.model.scaffold00361.4 | Papain (C1A) | 0.03 |
| evm.model.scaffold00361.5 | Papain (C1A) | 0.00 |
| evm.model.scaffold05371.19 | Papain (C1A) | 0.00 |
|  |  |  |
| evm.model.scaffold04907.4 | Aminopeptidase (M1) | 0.48 |
| evm.model.scaffold04050.14 | Aminopeptidase (M1) | 0.00 |
| evm.model.scaffold01045.57 | Adamalysin (M12B) | 0.91 |
| evm.model.scaffold00661.19 | Adamalysin (M12B) | 0.00 |
| evm.model.scaffold02564.8 | Neprilysin (M13) | 0.48 |
|  |  |  |
| evm.model.scaffold04746.6 | Chymotrypsin (S1A) | 9.42 |
| evm.model.scaffold02256.5 | Chymotrypsin (S1A) | 0.00 |
| evm.model.scaffold01295.9 | Kexin (S8B) | 550.25 |
| evm.model.scaffold05189.36 | Kexin (S8B) | 100.68 |
| evm.model.scaffold01965.12 | Kexin (S8B) | 44.42 |
| evm.model.scaffold02360.9 | Kexin (S8B) | 3.72 |
| evm.model.scaffold05183.2 | Kexin (S8B) | 0.00 |
|  |  |  |
| evm.model.scaffold04176.44 | Gamma-glutamyltransferase (T3) | 50.33 |
| evm.model.scaffold00889.1 | Gamma-glutamyltransferase (T3) | 0.48 |
|  |  |  |
| evm.model.scaffold03380.33 | Kazal (I1) | 0.12 |
| evm.model.scaffold04162.20 | Kunitz-BPTI (I2) | 8.08 |
| evm.model.scaffold04527.1 | Kunitz-BPTI (I02) | 0.43 |
| evm.model.scaffold05009.24 | Kunitz-BPTI (I02) | 0.31 |
| 2632_t | Kunitz-BPTI (I02) | 0.00 |
| evm.model.scaffold03129.2 | Serpin (I4) | 1.95 |
| evm.model.scaffold04979.7 | Serpin (I4) | 0.57 |
| evm.model.scaffold02335.2 | Serpin (I4) | 0.51 |
| evm.model.scaffold04527.41 | Serpin (I4) | 0.18 |
| evm.model.scaffold03756.1 | Serpin (I4) | 0.00 |
| evm.model.scaffold05193.11 | Serpin (I4) | 0.00 |
| evm.model.scaffold05481.16 | Cystatin (I25A) | 241.03 |
| evm.model.scaffold00041.17 | CTLA (I29) | 2.06 |
| evm.model.scaffold05371.18 | CTLA (I29) | 0.31 |
| evm.model.scaffold03055.4 | IC (I51) | 297.10 |
| evm.model.scaffold04913.47 | IC (I51) | 8.80 |
| evm.model.scaffold03055.5 | IC (I51) | 0.99 |
| scaffold03928-g175.t1 | IC (I51) | 0.00 |
| evm.model.scaffold00177.1 | Pro-eosinophil major basic protein (I63) | 1.90 |

*Expression level was determined using the mapped reads per kilobase of exon per million mapped reads in the spore cells.

**Table S9** Putative α/β-hydrolase fold proteins in *T. kitauei* and the two free-living cnidarians

| **Class** | **Superfamily** | **Number of predicted** **α/β-hydrolase fold proteins (% of total predicted α/β-hydrolase fold proteins )** | | |
| --- | --- | --- | --- | --- |
|  |  | ***T. kitauei*** | ***H. magnipapillata*** | ***N. vectensis*** |
| GGGX | Carboxylesterases | 16 (10.2) | 17 (3.4) | 52 (7.7) |
|  | Moraxella lipase 2 like | 5 (3.2) | 12 (2.4) | 19 (2.8) |
|  | Hormone sensitive lipases | 1 (0.6) | 18 (3.6) | 77 (11.5) |
|  | Sum | 22 (14.0) | 47 (9.4) | 148 (22.0) |
| GX | Moraxella lipase 3 like | 2 (1.3) | 8 (1.6) | 3 (0.4) |
|  | Cytosolic hydrolases | 5 (3.2) | 36 (7.2) | 47 (7.0) |
|  | Microsomal hydrolases | 100 (63.7) | 353 (70.9) | 389 (57.9) |
|  | Carboxylesterases | 3 (1.9) | 2 (0.4) | 6 (0.9) |
|  | Hydroxynitrile lyases | 0 (0.0) | 0 (0.0) | 1 (0.1) |
|  | Bacterial esterases | 0 (0.0) | 1 (0.2) | 0 (0.0) |
|  | Gastric lipases | 2 (1.3) | 6 (1.2) | 6 (0.9) |
|  | Burkholderia lipases | 1 (0.6) | 0 (0.0) | 1 (0.1) |
|  | Chloroflexus aurantiacus lipase like | 0 (0.0) | 0 (0.0) | 2 (0.3) |
|  | Thioesterases | 0 (0.0) | 2 (0.4) | 3 (0.4) |
|  | Lipoprotein lipases | 4 (2.5) | 6 (1.2) | 10 (1.5) |
|  | Bacterial esterases | 3 (1.9) | 4 (0.8) | 1 (0.1) |
|  | Lysophospholipase | 4 (2.5) | 4 (0.8) | 6 (0.9) |
|  | Filamentous fungi lipases | 3 (1.9) | 4 (0.8) | 9 (1.3) |
|  | Moraxella lipase 1 like | 0 (0.0) | 1 (0.2) | 0 (0.0) |
|  | Deacetylases | 0 (0.0) | 2 (0.4) | 1 (0.1) |
|  | Dienlactone hydrolases | 6 (3.8) | 6 (1.2) | 11 (1.6) |
|  | Xylanase esterases | 0 (0.0) | 2 (0.4) | 3 (0.4) |
|  | Antigen 85 | 0 (0.0) | 2 (0.4) | 1 (0.1) |
|  | Lysosomal protective protein like | 0 (0.0) | 2 (0.4) | 2 (0.3) |
|  | Acyl-transferase | 0 (0.0) | 3 (0.6) | 0 (0.0) |
|  | Cutinases | 0 (0.0) | 2 (0.4) | 2 (0.3) |
|  | Sum | 133 (84.7) | 446 (89.6) | 504 (75.0) |
| Y | Dipeptidyl peptidase IV like | 2 (1.3) | 3 (0.6) | 10 (1.5) |
|  | Prolyl endopeptidases | 0 (0.0) | 2 (0.4) | 4 (0.6) |
|  | Cocaine esterases | 0 (0.0) | 0 (0.0) | 5 (0.7) |
|  | Candida antarctica lipase A like | 0 (0.0) | 0 (0.0) | 1 (0.1) |
|  | Sum | 2 (1.3) | 5 (1.0) | 20 (3.0) |
|  | Total sum | 157 (100.0) | 498 (100.0) | 672 (100.0) |

**Table S10** Predicted secreted α/β hydrolases in *T. kitauei*

| **Gene_ID** | **Class** | **Superfamily** | **Family** | **Expression in myxospores of *T. kitauei**** |
| --- | --- | --- | --- | --- |
| evm.model.scaffold01482.1 | GX | Microsomal hydrolases | Proline iminopeptidases | 3675.42 |
| evm.model.scaffold03704.25 |  |  | Proline iminopeptidases | 868.32 |
| evm.model.scaffold05565.11 |  |  | Proline iminopeptidases | 374.12 |
| evm.model.scaffold04834.12 |  |  | Proline iminopeptidases | 184.78 |
| evm.model.scaffold04607.1 |  |  | Proline iminopeptidases | 113.48 |
| evm.model.scaffold03932.2 |  |  | Proline iminopeptidases | 104.86 |
| evm.model.scaffold04942.3 |  |  | Proline iminopeptidases | 97.37 |
| evm.model.scaffold03946.20 |  |  | Proline iminopeptidases | 66.47 |
| evm.model.scaffold04887.6 |  |  | Proline iminopeptidases | 66.06 |
| evm.model.scaffold03891.24 |  |  | Proline iminopeptidases | 64.17 |
| evm.model.scaffold00832.43 |  |  | Proline iminopeptidases | 8.42 |
| 18475_t |  |  | Proline iminopeptidases | 1.41 |
| evm.model.scaffold00629.8 |  |  | Proline iminopeptidases | 0.33 |
| evm.model.scaffold01172.20 |  |  | Proline iminopeptidases | 0.00 |
| evm.model.scaffold02355.3 |  |  | Proline iminopeptidases | 0.00 |
| evm.model.scaffold03736.1 |  |  | Proline iminopeptidases | 0.00 |
| evm.model.scaffold05473.15 |  |  | Proline iminopeptidases | 0.00 |
| scaffold02355-g79.t1 |  |  | Proline iminopeptidases | 0.00 |
| scaffold02916-g509.t1 |  |  | Proline iminopeptidases | 0.00 |
| evm.model.scaffold01113.21 |  | Lysophospholipase | Lysophospholipase | 3.46 |
|  |  |  |  |  |
| evm.model.scaffold04623.106 | Y | Dipeptidyl peptidase IV like | Dipeptidyl peptidase IV like | 5.58 |

*Expression level was determined using the mapped reads per kilobase of exon per million mapped reads in the spore cells.

**Table S11** Predicted carbohydrate-active enzymes in three cnidarians

| **Family** | **Potential enzyme activity** | **Number of predicted carbohydrate-active enzymes (%** **of total predicted carbohydrate-active enzymes)** | | |
| --- | --- | --- | --- | --- |
|  |  | ***T. kitauei*** | ***H. magnipapillata*** | ***N. vectensis*** |
| CBM42 | arabinofuranose-binding function | 0 (0) | 15 (4.1) | 3 (0.4) |
| CBM57 | glycosidases-binding function | 0 (0) | 1 (0.3) | 1 (0.1) |
| CBM44 | cellulose and xyloglucan-binding function | 0 (0) | 0 (0) | 1 (0.1) |
| CBM9 | cellulose-binding function | 0 (0) | 0 (0) | 2 (0.3) |
| CBM14 | chitin-binding function | 2 (1.3) | 14 (3.8) | 15 (2.1) |
| CBM50 | chitopentaose--binding function | 1 (0.7) | 2 (0.5) | 1 (0.1) |
| CBM66 | fructans-binding function | 0 (0) | 0 (0) | 1 (0.1) |
| CBM47 | fucose-binding function | 0 (0) | 5 (1.4) | 25 (3.5) |
| CBM13 | galactose or mannose-binding function | 1 (0.7) | 2 (0.5) | 3 (0.4) |
| CBM32 | galactose, lactose-binding function | 2 (1.3) | 22 (6.0) | 252 (34.9) |
| CBM48 | glycogen-binding function | 0 (0) | 0 (0) | 2 (0.3) |
| CBM38 | inulin-binding function | 0 (0) | 1 (0.3) | 5 (0.7) |
| CBM67 | L-rhamnose binding activity | 0 (0) | 0 (0) | 1 (0.1) |
| CBM23 | mannan-binding function | 0 (0) | 0 (0) | 1 (0.1) |
| CBM40 | sialic acid-binding function | 1 (0.7) | 5 (1.4) | 23 (3.2) |
| CBM20 | starch-binding function | 0 (0) | 1 (0.3) | 1 (0.1) |
| CBM21 | starch-binding function | 1 (0.7) | 0 (0) | 9 (1.2) |
| CBM37 | xylan and chitin-binding function | 0 (0) | 1 (0.3) | 1 (0.1) |
| CBM35 | xylan, mannans, manno-oligosaccharides and β-galactan-binding function | 0 (0) | 1 (0.3) | 0 (0) |
|  | Sum | 8 (5.3) | 70 (19.0) | 347 (48.0) |
|  |  |  |  |  |
| CE3 | acetyl xylan esterase | 0 (0) | 9 (2.4) | 12 (1.7) |
| CE6 | acetyl xylan esterase | 0 (0) | 1 (0.3) | 0 (0) |
| CE7 | acetyl xylan esterase | 0 (0) | 1 (0.3) | 0 (0) |
| CE1 | acetyl xylan esterase | 2 (1.3) | 8 (2.2) | 15 (2.1) |
| CE4 | acetyl xylan esterase, chitin deacetylase | 0 (0) | 13 (3.5) | 4 (0.6) |
| CE10 | arylesterase | 9 (6.0) | 6 (1.6) | 29 (4.0) |
| CE14 | N-acetyl-1-D-myo-inosityl-2-amino-2-deoxy-α -D-glucopyranoside deacetylase | 0 (0) | 1 (0.3) | 2 (0.3) |
| CE9 | N-acetylglucosamine 6-phosphate deacetylase | 0 (0) | 1 (0.3) | 1 (0.1) |
| CE12 | pectin acetylesterase | 0 (0) | 1 (0.3) | 1 (0.1) |
| CE13 | pectin acetylesterase | 0 (0) | 4 (1.1) | 4 (0.6) |
| CE8 | pectin methylesterase | 0 (0) | 0 (0) | 1 (0.1) |
|  | Sum | 11 (7.3) | 45 (12.2) | 69 (9.5) |
|  |  |  |  |  |
| GH109 | α-N-acetylgalactosaminidase | 0 (0) | 8 (2.2) | 5 (0.7) |
| GH89 | α-N-acetylglucosaminidase | 0 (0) | 2 (0.5) | 1 (0.1) |
| GH85 | endo-β-N-acetylglucosaminidase | 2 (1.3) | 2 (0.5) | 1 (0.1) |
| GH13 | α-amylase | 1 (0.7) | 3 (0.8) | 4 (0.6) |
| GH18 | chitinase | 0 (0) | 24 (6.5) | 10 (1.4) |
| GH19 | chitinase | 0 (0) | 4 (1.1) | 0 (0) |
| GH5 | chitosanase | 0 (0) | 5 (1.4) | 5 (0.7) |
| GH9 | endoglucanase | 0 (0) | 0 (0) | 1 (0.1) |
| GH74 | endoglucanase | 11 (7.3) | 0 (0) | 5 (0.7) |
| GH29 | α-L-fucosidase | 0 (0) | 4 (1.1) | 10 (1.4) |
| GH27 | α-galactosidase | 0 (0) | 0 (0) | 3 (0.4) |
| GH2 | β-galactosidase | 0 (0) | 2 (0.5) | 2 (0.3) |
| GH35 | β-galactosidase | 0 (0) | 3 (0.8) | 7 (1.0) |
| GH15 | glucoamylase | 0 (0) | 0 (0) | 1 (0.1) |
| GH84 | N-acetyl β-glucosaminidase | 1 (0.7) | 2 (0.5) | 0 (0) |
| GH116 | acid β-glucosidase | 0 (0) | 4 (1.1) | 4 (0.6) |
| GH31 | α-glucosidase | 1 (0.7) | 8 (2.2) | 3 (0.4) |
| GH1 | β-glucosidase | 1 (0.7) | 0 (0) | 2 (0.3) |
| GH3 | β-glucosidase | 0 (0) | 1 (0.3) | 0 (0) |
| GH17 | glucan endo-1,3-β-glucosidase | 0 (0) | 5 (1.4) | 0 (0) |
| GH4 | maltose-6-phosphate glucosidase | 1 (0.7) | 0 (0) | 0 (0) |
| GH63 | processing α-glucosidase | 0 (0) | 1 (0.3) | 4 (0.6) |
| GH30 | glucosylceramidase | 0 (0) | 0 (0) | 2 (0.3) |
| GH79 | β-glucuronidase | 0 (0) | 8 (2.2) | 2 (0.3) |
| GH20 | β-hexosaminidase | 0 (0) | 10 (2.7) | 7 (1.0) |
| GH56 | hyaluronidase | 0 (0) | 1 (0.3) | 3 (0.4) |
| GH73 | peptidoglycan hydrolase | 0 (0) | 0 (0) | 1 (0.1) |
| GH39 | α-L-iduronidase | 0 (0) | 0 (0) | 2 (0.3) |
| GH24 | lysozyme | 1 (0.7) | 0 (0) | 1 (0.1) |
| GH23 | lysozyme type G | 1 (0.7) | 0 (0) | 0 (0) |
| GH47 | α-mannosidase | 1 (0.7) | 6 (1.6) | 4 (0.6) |
| GH99 | glycoprotein endo-α-1,2-mannosidase | 0 (0) | 1 (0.3) | 1 (0.1) |
| GH92 | mannosyl-oligosaccharide α-1,2-mannosidase | 0 (0) | 0 (0) | 1 (0.1) |
| GH38 | α-mannosidase | 1 (0.7) | 5 (1.4) | 3 (0.4) |
| GH94 | cellobiose phosphorylase | 1 (0.7) | 0 (0) | 0 (0) |
| GH33 | sialidase or neuraminidase | 2 (1.3) | 0 (0) | 1 (0.1) |
| GH65 | α,α-trehalase | 0 (0) | 1 (0.3) | 4 (0.6) |
| GH10 | endo-1,4-β-xylanase | 0 (0) | 0 (0) | 1 (0.1) |
| GH16 | xyloglucan:xyloglucosyltransferase | 0 (0) | 2 (0.5) | 0 (0) |
|  | Sum | 25 (16.6) | 112 (30.4) | 101 (14.0) |
|  |  |  |  |  |
| GT12 | [N-acetylneuraminyl]-galactosylglucosylceramide N-acetylgalactosaminyltransferase | 9 (6.0) | 0 (0) | 1 (0.1) |
| GT27 | polypeptide α-*N*-acetylgalactosaminyltransferase | 2 (1.3) | 11 (3.0) | 10 (1.4) |
| GT13 | α-1,3-mannosyl-glycoprotein β-1,2-*N*-acetylglucosaminyltransferase | 15 (9.9) | 3 (0.8) | 3 (0.4) |
| GT16 | α-1,6-mannosyl-glycoprotein β-1,2-*N*-acetylglucosaminyltransferase | 0 (0) | 1 (0.3) | 1 (0.1) |
| GT14 | β-1,3-galactosyl-O-glycosyl-glycoprotein β-1,6-*N*-acetylglucosaminyltransferase | 0 (0) | 0 (0) | 3 (0.4) |
| GT49 | β-1,3-*N*-acetylglucosaminyltransferase | 0 (0) | 1 (0.3) | 2 (0.3) |
| GT17 | β-1,4-mannosyl-glycoprotein β-1,4-*N*-acetylglucosaminyltransferase | 0 (0) | 0 (0) | 3 (0.4) |
| GT9 | lipopolysaccharide N-acetylglucosaminyltransferase | 1 (0.7) | 0 (0) | 0 (0) |
| GT31 | N-acetyllactosaminide β-1,3-*N*-acetylglucosaminyltransferase | 22 (14.6) | 26 (7.1) | 42 (5.8) |
| GT54 | UDP-GlcNAc: α-1,3-D-mannoside β-1,4-*N*-acetylglucosaminyltransferase | 2 (1.3) | 4 (1.1) | 9 (1.2) |
| GT41 | UDP-GlcNAc: peptide β-N-acetylglucosaminyltransferase | 1 (0.7) | 1 (0.3) | 1 (0.1) |
| GT64 | UDP-GlcNAc: heparan α-N-acetylhexosaminyltransferase | 0 (0) | 2 (0.5) | 3 (0.4) |
| GT2 | cellulose/chitin synthase | 10 (6.6) | 7 (1.9) | 9 (1.2) |
| GT10 | galactoside α-1,3/1,4-L-fucosyltransferase | 14 (9.3) | 28 (7.6) | 18 (2.5) |
| GT65 | GDP-Fuc: protein O-α-fucosyltransferase | 0 (0) | 2 (0.5) | 2 (0.3) |
| GT68 | GDP-Fuc: protein O-α-fucosyltransferase | 0 (0) | 1 (0.3) | 3 (0.4) |
| GT23 | N-acetyl-β-D-glucosaminide α-1,6-L-fucosyltransferase | 13 (8.6) | 3 (0.8) | 6 (0.8) |
| GT28 | 1,2-diacylglycerol 3-β-galactosyltransferase | 0 (0) | 1 (0.3) | 1 (0.1) |
| GT8 | lipopolysaccharide α-1,3-galactosyltransferase | 1 (0.7) | 4 (1.1) | 10 (1.4) |
| GT92 | UDP-Gal: N-glycan core α-1,6-fucoside β-1,4-galactosyltransferase | 0 (0) | 0 (0) | 3 (0.4) |
| GT25 | lipopolysaccharide β-1,4-galactosyltransferase | 1 (0.7) | 1 (0.3) | 1 (0.1) |
| GT18 | α-1,3(6)-mannosylglycoprotein β-1,6-*N*-acetyl-glucosaminyltransferase | 0 (0) | 2 (0.5) | 6 (0.8) |
| GT75 | UDP-Glc: self-glucosylating β-glucosyltransferase | 0 (0) | 1 (0.3) | 3 (0.4) |
| GT57 | Dol-P-Glc: α-1,3-glucosyltransferase | 0 (0) | 2 (0.5) | 2 (0.3) |
| GT59 | Dol-P-Glc: Glc_2_Man_9_GlcNAc_2_-PP-Dol α-1,2-glucosyltransferase | 0 (0) | 1 (0.3) | 0 (0) |
| GT21 | UDP-Glc: ceramide β-glucosyltransferase | 1 (0.7) | 0 (0) | 2 (0.3) |
| GT24 | UDP-Glc: glycoprotein α-glucosyltransferase | 1 (0.7) | 2 (0.5) | 3 (0.4) |
| GT1 | UDP-glucuronosyltransferase | 0 (0) | 1 (0.3) | 9 (1.2) |
| GT43 | β-glucuronyltransferase | 1 (0.7) | 2 (0.5) | 4 (0.6) |
| GT47 | heparan β-glucuronyltransferase | 0 (0) | 1 (0.3) | 0 (0) |
| GT35 | glycogen or starch phosphorylase | 1 (0.7) | 2 (0.5) | 1 (0.1) |
| GT3 | glycogen synthase | 1 (0.7) | 1 (0.3) | 1 (0.1) |
| GT7 | lactose synthase | 4 (2.6) | 6 (1.6) | 6 (0.8) |
| GT50 | Dol-P-Man α-1,4-mannosyltransferase | 0 (0) | 1 (0.3) | 1 (0.1) |
| GT22 | Dol-P-Man α-mannosyltransferase | 0 (0) | 4 (1.1) | 4 (0.6) |
| GT76 | Dol-P-Man: α-1,6-mannosyltransferase | 0 (0) | 0 (0) | 1 (0.1) |
| GT58 | Dol-P-Man: dolichol pyrophosphate-mannose α-1,3-mannosyltransferase | 0 (0) | 1 (0.3) | 1 (0.1) |
| GT39 | Dol-P-Man: protein α-mannosyltransferase | 1 (0.7) | 3 (0.8) | 4 (0.6) |
| GT33 | GDP-Man: chitobiosyldiphosphodolichol β-mannosyltransferase | 1 (0.7) | 1 (0.3) | 1 (0.1) |
| GT51 | murein polymerase | 0 (0) | 0 (0) | 1 (0.1) |
| GT30 | CMP-β-KDO: α-3-deoxy-D-manno-octulosonic-acid (KDO) transferase | 1 (0.7) | 0 (0) | 0 (0) |
| GT66 | Dol-PP-α-oligosaccharide: protein β-oligosaccharyltransferase | 1 (0.7) | 3 (0.8) | 3 (0.4) |
| GT4 | sucrose synthase | 3 (2.0) | 5 (1.4) | 10 (1.4) |
| GT61 | β-1,2-xylosyltransferase | 0 (0) | 1 (0.3) | 3 (0.4) |
| GT90 | UDP-Xyl: (mannosyl) glucuronoxylomannan/galactoxylomannan β-1,2-xylosyltransferase | 0 (0) | 3 (0.8) | 2 (0.3) |
|  | Sum | 107 (70.9) | 139 (37.8) | 199 (27.5) |
|  |  |  |  |  |
| PL12 | heparin-sulfate lyase | 0 (0) | 1 (0.3) | 1 (0.1) |
| PL8 | hyaluronate lyase | 0 (0) | 1 (0.3) | 5 (0.7) |
| PL9 | pectate lyase | 0 (0) | 0 (0) | 1 (0.1) |
|  | Sum | 0 (0) | 2 (0.5) | 7 (1.0) |
|  | Total sum | 151 (100.0) | 368 (100.0) | 723 (100.0) |

**Table S12** Predicted secreted carbohydrate-active enzymes in *T. kitauei*

| **Gene_ID** | **Family** | **Potential activity** | **Expression in myxospores of *T. kitauei**** |
| --- | --- | --- | --- |
| evm.model.scaffold05009.24 | CBM14 | chitin-binding function | 0.31 |
| evm.model.scaffold00177.1 | CBM32 | galactose, lactose-binding function | 1.90 |
| evm.model.scaffold03426.32 | GH31 | α-glucosidase | 11.06 |
| evm.model.scaffold00266.1 | GH38 | α-mannosidase | 0 |
| evm.model.scaffold01140.7 | GT13 | α-1,3-mannosyl-glycoprotein β-1,2-*N*-acetylglucosaminyltransferase | 0.06 |
| evm.model.scaffold05001.1 | GT13 | α-1,3-mannosyl-glycoprotein β-1,2-*N*-acetylglucosaminyltransferase | 0.06 |
| evm.model.scaffold03704.50 | GT13 | α-1,3-mannosyl-glycoprotein β-1,2-*N*-acetylglucosaminyltransferase | 0.21 |
| evm.model.scaffold01087.6 | GT13 | α-1,3-mannosyl-glycoprotein β-1,2-*N*-acetylglucosaminyltransferase | 3.23 |
| evm.model.scaffold01087.1 | GT13 | α-1,3-mannosyl-glycoprotein β-1,2-*N*-acetylglucosaminyltransferase | 4.45 |
| evm.model.scaffold03631.5 | GT23 | N-acetyl-β-D-glucosaminide α-1,6-L-fucosyltransferase | 0 |
| evm.model.scaffold05284.3 | GT23 | N-acetyl-β-D-glucosaminide α-1,6-L-fucosyltransferase | 0.39 |
| evm.model.scaffold03631.4 | GT23 | N-acetyl-β-D-glucosaminide α-1,6-L-fucosyltransferase | 0.49 |
| evm.model.scaffold03631.8 | GT23 | N-acetyl-β-D-glucosaminide α-1,6-L-fucosyltransferase | 0.50 |
| evm.model.scaffold05284.4 | GT23 | N-acetyl-β-D-glucosaminide α-1,6-L-fucosyltransferase | 0.87 |
| evm.model.scaffold03631.6 | GT23 | N-acetyl-β-D-glucosaminide α-1,6-L-fucosyltransferase | 1.31 |
| evm.model.scaffold05284.5 | GT23 | N-acetyl-β-D-glucosaminide α-1,6-L-fucosyltransferase | 1.38 |
| scaffold04448-g450.t1 | GT27 | polypeptide α-N-acetylgalactosaminyltransferase | 0.18 |
| evm.model.scaffold02707.12 | GT31 | N-acetyllactosaminide β-1,3-*N*-acetylglucosaminyltransferase | 0 |
| evm.model.scaffold03463.16 | GT31 | N-acetyllactosaminide β-1,3-*N*-acetylglucosaminyltransferase | 0.12 |
| evm.model.scaffold00726.6 | GT31 | N-acetyllactosaminide β-1,3-*N*-acetylglucosaminyltransferase | 25.79 |
| evm.model.scaffold01103.2 | GT33 | GDP-Man: chitobiosyldiphosphodolichol β-mannosyltransferase | 0.13 |
| scaffold03453-g319.t1 | GT43 | β-glucuronyltransferase | 0.19 |
| evm.model.scaffold00051.1 | GT54 | UDP-GlcNAc: α-1,3-D-mannoside β-1,4-*N*-acetylglucosaminyltransferase | 0 |

*The expression level was determined using the mapped reads per kilobase of exon per million mapped reads in the spore cells.

**Table S13** Putative protease inhibitors in *T. kitauei* and other organisms

| **Family** | **Number of predicted proteases inhibitors (% of total predicted proteases inhibitors )** | | | | | |
| --- | --- | --- | --- | --- | --- | --- |
|  | ***T. kitauei*** | ***H. magnipapillata*** | | ***N. vectensis*** | ***S. mansoni*** | ***E. multilocularis*** |
| I01 | 4 (1.9) | | 24 (25.5) | 45 (27.4) | 4 (12.5) | 3 (7.5) |
| I02 | 5 (2.4) | | 7 (7.4) | 23 (14.0) | 7 (21.9) | 22 (55.0) |
| I04 | 182 (88.3) | | 3 (3.2) | 5 (3.0) | 9 (28.1) | 5 (12.5) |
| I08 | 0 (0.0) | | 5 (5.3) | 4 (2.4) | 0 (0.0) | 0 (0.0) |
| I12 | 0 (0.0) | | 0 (0.0) | 1 (0.6) | 0 (0.0) | 0 (0.0) |
| I15 | 0 (0.0) | | 7 (7.4) | 4 (2.4) | 0 (0.0) | 1 (2.5) |
| I17 | 0 (0.0) | | 2 (2.1) | 5 (3.0) | 0 (0.0) | 0 (0.0) |
| I19 | 0 (0.0) | | 1 (1.1) | 0 (0.0) | 0 (0.0) | 0 (0.0) |
| I21 | 0 (0.0) | | 0 (0.0) | 0 (0.0) | 0 (0.0) | 1 (2.5) |
| I25A | 1 (0.5) | | 1 (1.1) | 2 (1.2) | 1 (3.1) | 1 (2.5) |
| I25B | 0 (0.0) | | 5 (5.3) | 1 (0.6) | 3 (9.4) | 1 (2.5) |
| I29 | 2 (1.0) | | 0 (0.0) | 2 (1.2) | 0 (0.0) | 0 (0.0) |
| I31 | 0 (0.0) | | 2 (2.1) | 19 (11.6) | 0 (0.0) | 0 (0.0) |
| I32 | 0 (0.0) | | 1 (1.1) | 3 (1.8) | 3 (9.4) | 2 (5.0) |
| I35 | 0 (0.0) | | 3 (3.2) | 4 (2.4) | 0 (0.0) | 0 (0.0) |
| I39 | 0 (0.0) | | 11 (11.7) | 9 (5.5) | 2 (6.3) | 2 (5.0) |
| I50B | 0 (0.0) | | 0 (0.0) | 2 (1.2) | 0 (0.0) | 0 (0.0) |
| I51 | 10 (4.9) | | 0 (0.0) | 3 (1.8) | 1 (3.1) | 1 (2.5) |
| I52 | 1 (0.5) | | 0 (0.0) | 0 (0.0) | 0 (0.0) | 0 (0.0) |
| I63 | 1 (0.5) | | 21 (22.3) | 31 (18.9) | 2 (6.3) | 1 (2.5) |
| I71 | 0 (0.0) | | 1 (1.1) | 1 (0.6) | 0 (0.0) | 0 (0.0) |
| Total sum | 206 (100.0) | | 94 (100.0) | 164 (100.0) | 32 (100.0) | 40 (100.0) |

**Table S14**Predicted low-density lipoprotein receptors in *T. kitauei*

| **Gene_ID** | **Expression level in the myxospore stage of *T. kitauei**** |
| --- | --- |
| evm.model.scaffold03983.19 | 6.21 |
| evm.model.scaffold05175.5 | 0 |
| scaffold04423-g121.t1 | 0.06 |
| evm.model.scaffold05175.4 | 0 |
| evm.model.scaffold04712.6 | 258.58 |
| evm.model.scaffold04311.4 | 0 |
| evm.model.scaffold03454.25 | 111.91 |
| evm.model.scaffold03076.3 | 6.11 |
| evm.model.scaffold04402.1 | 0 |
| 147_t | 0.16 |
| scaffold05430-g559.t1 | 0.02 |
| evm.model.scaffold01473.27 | 0.00 |
| evm.model.scaffold01473.33 | 0.37 |
| evm.model.scaffold01473.17 | 0 |
| evm.model.scaffold00727.9 | 1.80 |
| evm.model.scaffold05371.6 | 0 |
| evm.model.scaffold00661.16 | 0 |
| 13915_t | 0 |
| evm.model.scaffold02577.73 | 1.08 |
| evm.model.scaffold01473.36 | 0.08 |
| evm.model.scaffold01396.1 | 0 |
| evm.model.scaffold02630.26 | 0 |
| 19607_t | 0 |
| evm.model.scaffold04180.10 | 6.25 |
| scaffold00786-g22.t1 | 0 |
| evm.model.scaffold02793.15 | 0 |
| evm.model.scaffold05473.12 | 144.54 |
| evm.model.scaffold03257.1 | 0 |
| evm.model.scaffold05633.24 | 72.58 |
| evm.model.scaffold03954.12 | 0.27 |
| scaffold03844-g609.t1 | 0 |
| scaffold02256-g661.t1 | 0 |
| evm.model.scaffold02750.44 | 6.07 |
| evm.model.scaffold01473.21 | 0 |
| evm.model.scaffold02417.1 | 8.97 |
| evm.model.scaffold05258.10 | 2.73 |
| evm.model.scaffold01493.21 | 65.03 |
| evm.model.scaffold02793.10 | 0 |
| 17335_t | 0.04 |
| evm.model.scaffold00249.1 | 0 |
| evm.model.scaffold01724.2 | 0 |
| evm.model.scaffold04876.5 | 45.03 |
| evm.model.scaffold00656.4 | 0 |
| evm.model.scaffold04998.13 | 22.73 |
| evm.model.scaffold03946.34 | 1.96 |
| evm.model.scaffold01473.37 | 0 |
| evm.model.scaffold05633.21 | 0 |
| evm.model.scaffold00005.38 | 0 |
| evm.model.scaffold04845.21 | 0 |
| evm.model.scaffold04607.9 | 3.30 |
| evm.model.scaffold05633.17 | 0 |
| scaffold02793-g41.t1 | 0 |
| evm.model.scaffold05189.2 | 24.80 |
| scaffold05299-g420.t1 | 0 |
| 3314_t | 0 |
| evm.model.scaffold03453.17 | 1.40 |
| evm.model.scaffold04527.55 | 16.63 |
| evm.model.scaffold04527.49 | 40.51 |
| evm.model.scaffold02356.2 | 0 |
| scaffold05595-g565.t1 | 0.28 |
| scaffold05258-g28.t1 | 0.17 |
| evm.model.scaffold02615.11 | 0 |
| evm.model.scaffold00727.6 | 0.09 |
| evm.model.scaffold03454.23 | 50.03 |
| evm.model.scaffold00864.10 | 0 |
| scaffold02335-g275.t1 | 0 |
| scaffold03484-g495.t1 | 0 |
| evm.model.scaffold00656.2 | 0 |
| scaffold01419-g8.t1 | 0.04 |
| evm.model.scaffold03971.3 | 0.61 |
| evm.model.scaffold03253.2 | 0.34 |
| evm.model.scaffold04607.5 | 8.09 |
| scaffold02256-g663.t1 | 0 |
| evm.model.scaffold04423.69 | 134.07 |
| scaffold01944-g326.t1 | 0 |
| evm.model.scaffold02817.1 | 0 |
| scaffold02174-g476.t1 | 0 |
| evm.model.scaffold03287.3 | 0 |
| evm.model.scaffold04527.56 | 7.42 |
| evm.model.scaffold04712.1 | 26.99 |
| scaffold04754-g176.t1 | 0.01 |
| evm.model.scaffold01724.5 | 0.25 |
| evm.model.scaffold03015.12 | 30.79 |
| 5155_t | 0 |
| evm.model.scaffold00491.10 | 0.45 |
| scaffold03946-g86.t1 | 0 |
| evm.model.scaffold01983.4 | 0 |
| evm.model.scaffold02256.10 | 0 |
| evm.model.scaffold05189.22 | 4.33 |
| scaffold01419-snap.31 | 0.20 |
| evm.model.scaffold01724.1 | 0.40 |
| evm.model.scaffold02481.33 | 0.53 |
| scaffold03748-g185.t1 | 0.02 |
| scaffold02481.path1.gene43 | 0.29 |
| 6055_t | 0 |
| evm.model.scaffold00872.9 | 14.07 |
| scaffold02967-g76.t1 | 0 |
| 15371_t | 0 |
| evm.model.scaffold04746.6 | 9.42 |
| evm.model.scaffold01673.6 | 0 |
| evm.model.scaffold01762.1 | 0 |
| 2151_t | 0.08 |
| evm.model.scaffold03051.3 | 0 |
| scaffold02659-g264.t1 | 0.08 |
| evm.model.scaffold01673.7 | 0 |
| evm.model.scaffold02256.5 | 0 |
| evm.model.scaffold02793.14 | 0 |
| evm.model.scaffold04809.1 | 0 |
| evm.model.scaffold04766.21 | 0 |
| evm.model.scaffold05197.3 | 1.68 |
| evm.model.scaffold03900.1 | 0 |
| evm.model.scaffold01493.6 | 20.07 |
| evm.model.scaffold05616.9 | 0 |
| evm.model.scaffold00167.2 | 0 |
| evm.model.scaffold01755.2 | 190.33 |
| evm.model.scaffold00727.3 | 0.08 |
| evm.model.scaffold04425.2 | 1.28 |
| evm.model.scaffold05189.27 | 0 |
| evm.model.scaffold03804.1 | 0 |
| evm.model.scaffold04913.24 | 47.04 |
| evm.model.scaffold02253.3 | 0 |
| evm.model.scaffold00491.20 | 13.56 |

*The expression level was determined using the mapped reads per kilobase of exon per million mapped reads in the spore cells.

**Table S15** Comparison of putative *T. kitauei* and *H. magnipapillata* transporters according to TC class, superfamily and substrate

|  |  | ***T. kitauei*** | ***H. magnipapillata*** | ***T. kitauei* / *H. magnipapillata*** |
| --- | --- | --- | --- | --- |
| **TC class** | **Superfamily** |  |  |  |
| Channels | ENaC/P2X |  | 18 (2) |  |
|  | [Major intrinsic protein](http://www.tcdb.org/superfamily.php?id=43) (MIP) |  | 8 (1) |  |
|  | VIC | 15 (6) | 206 (25) | 7% |
|  | General bacterial porin (GBP) | 3 (1) |  |  |
| Secondary carriers | APC | 24 (10) | 93 (11) | 26% |
|  | [Cation diffusion Facilitator](http://www.tcdb.org/superfamily.php?id=29)(CDF) | 6 (3) | 17 (2) | 35% |
|  | [Drug/metabolite transporter](http://www.tcdb.org/superfamily.php?id=54) (DMT) | 9 (4) | 18 (2) | 50% |
|  | IT | 4 (2) | 1 (0) | 400% |
|  | [Mitochondrial carrier](http://www.tcdb.org/superfamily.php?id=26) (MC) | 13 (6) | 43 (5) | 30% |
|  | MFS | 56 (24) | 136 (16) | 41% |
| Primary active transporters | ABC | 33 (14) | 87 (10) | 38% |
|  | P-ATPase | 20 (9) | 51 (6) | 35% |
|  |  |  |  |  |
| **Substrate category** | **Substrate subcategory** |  |  |  |
| Organic |  | 102 (44) | 249 (30) | 41% |
|  | Amino acids/peptides | 23 (10) | 55 (7) | 42% |
|  | Drugs/toxic compounds | 28 (12) | 31 (4) | 90% |
|  | Mono-, di-, tri-carboxylates | 1 (0) | 13 (2) | 8% |
|  | Nucleotides/nucleosides | 6 (3) | 14 (2) | 43% |
|  | Sugars and derivative | 24 (10) | 54 (6) | 44% |
|  | Vitamins and cofactors | 7 (3) | 26 (3) | 27% |
|  | Others | 13 (6) | 56 (7) | 23% |
| Inorganic |  | 86 (37) | 441 (53) | 20% |
|  | Anions | 34 (15) | 67 (8) | 51% |
|  | Cations | 52 (23) | 374 (45) | 14% |
| Macromolecule |  | 25 (11) | 35 (4) | 71% |
|  | Proteins/Lipoproteins | 5 (2) | 8 (1) | 63% |
|  | Lipids | 20 (9) | 27 (3) | 74% |
| unknown |  | 18 (8) | 112 (13) | 16% |
|  | Total | 231 (100) | 837 (100) | 28% |

Numbers in parentheses are % of total transporters
